# Investigation on Novel *E/Z* 2-Benzylideneindan-1-One-Based Photoswitches with AChE and MAO-B Dual Inhibitory Activity

**DOI:** 10.3390/molecules28155857

**Published:** 2023-08-03

**Authors:** Marco Paolino, Modesto de Candia, Rosa Purgatorio, Marco Catto, Mario Saletti, Anna Rita Tondo, Orazio Nicolotti, Andrea Cappelli, Antonella Brizzi, Claudia Mugnaini, Federico Corelli, Cosimo D. Altomare

**Affiliations:** 1 Department of Biotechnology, Chemistry and Pharmacy, University of Siena, Via A. Moro 2, I-53100 Siena, Italy; paolino3@unisi.it (M.P.); mario.saletti2@unisi.it (M.S.); andrea.cappelli@unisi.it (A.C.); antonella.brizzi@unisi.it (A.B.); claudia.mugnaini@unisi.it (C.M.); federico.corelli@unisi.it (F.C.); 2 Department of Pharmacy-Pharmaceutical Sciences, University of Bari Aldo Moro, Via E. Orabona 4, I-70125 Bari, Italy; modesto.decandia@uniba.it (M.d.C.); rosa.purgatorio@uniba.it (R.P.); marco.catto@uniba.it (M.C.); anna.tondo@uniba.it (A.R.T.); orazio.nicolotti@uniba.it (O.N.)

**Keywords:** neurodegenerative diseases, cholinesterase inhibitors, monoaminoxidase inhibitors, photopharmacology

## Abstract

The multitarget therapeutic strategy, as opposed to the more traditional ‘one disease-one target-one drug’, may hold promise in treating multifactorial neurodegenerative syndromes, such as Alzheimer’s disease (AD) and related dementias. Recently, combining a photopharmacology approach with the multitarget-directed ligand (MTDL) design strategy, we disclosed a novel donepezil-like compound, namely 2-(4-((diethylamino)methyl)benzylidene)-5-methoxy-2,3-dihydro-1*H*-inden-1-one (**1a**), which in the *E* isomeric form (and about tenfold less in the UV-B photo-induced isomer *Z*) showed the best activity as dual inhibitor of the AD-related targets acetylcholinesterase (AChE) and monoamine oxidase B (MAO-B). Herein, we investigated further photoisomerizable 2-benzylideneindan-1-one analogs **1b**–**h** with the unconjugated tertiary amino moiety bearing alkyls of different bulkiness and lipophilicity. For each compound, the thermal stable *E* geometric isomer, along with the *E/Z* mixture as produced by UV-B light irradiation in the photostationary state (PSS, 75% *Z*), was investigated for the inhibition of human ChEs and MAOs. The pure *E*-isomer of the N-benzyl(ethyl)amino analog **1h** achieved low nanomolar AChE and high nanomolar MAO-B inhibition potencies (IC_50_s 39 and 355 nM, respectively), whereas photoisomerization to the *Z* isomer (75% *Z* in the PSS mixture) resulted in a decrease (about 30%) of AChE inhibitory potency, and not in the MAO-B one. Molecular docking studies were performed to rationalize the different *E*/*Z* selectivity of **1h** toward the two target enzymes.

## 1. Introduction

The incidence of neurodegenerative diseases in the aging world population, particularly Alzheimer’s disease (AD) and Parkinson’s disease (PD), is influenced by the increase in average life expectancy and unregulated lifestyle, as highlighted in the recent World Alzheimer Reports [1]. In the absence of disease-modifying drugs, AD and PD are considered incurable, and this serious health threat is expected to increase in the near future [2]. By limiting oxidative stress and the associated inflammatory state, as well as by fine-tuning the concentration of neurotransmitters, it is possible to apply some palliative pharmacological therapies to slow down the progression of neurodegenerative diseases and control their symptoms. Among the various pharmacological targets identified, monoamine oxidases (MAOs) and cholinesterases (ChEs) are of particular interest.

**Figure 1:**
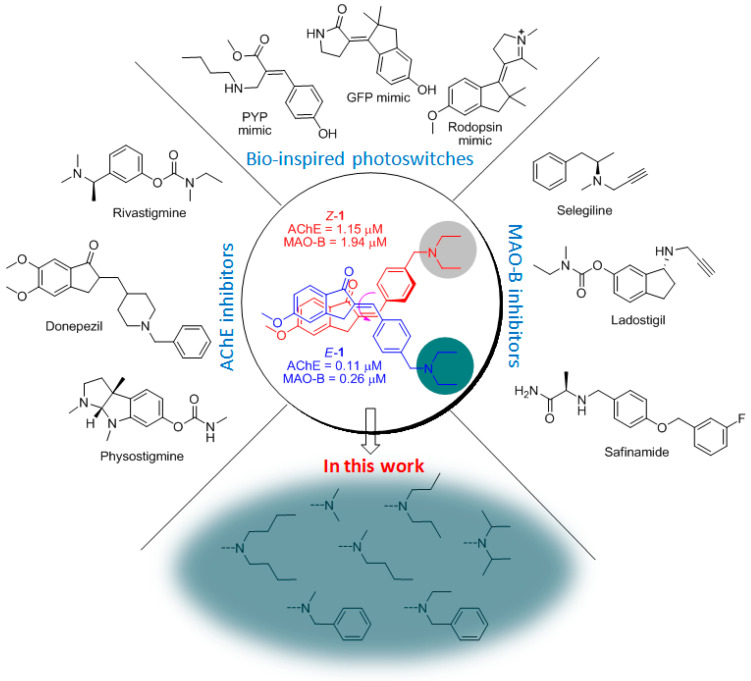
Photoisomerizable dual AChE/MAO-B inhibitors synthesized in this study aim at optimizing the tertiary amino head of the hit compound **1a**; the *E/Z* isomers of **1a** are surrounded by known AChE-Is, MAO-B-Is, and synthetic light-gated molecular switches and rotors.

MAOs are mitochondrial enzymes responsible for the oxidative catabolism of endogenous monoamines and xenobiotics. Their levels increase with age, resulting in increased production of reactive oxygen species (ROS) as by-products of the oxidation process, which ultimately leads to neuroinflammation and subsequent brain tissue damage. In humans, two enzyme isoforms, MAOs A and B, have been identified [3], both playing roles in the inactivation of endogenous monoaminergic neurotransmitters, albeit with different specificities. MAO-A is mainly responsible for the catabolism of norepinephrine, serotonin, and epinephrine, whereas MAO-B is more selective for tyramine and dopamine [4,5]. Selective MAO-B inhibitors (MAO-B Is) showed neuroprotective effects due to a reduction in ROS formation and improved cognitive abilities in patients with neurodegenerative disorders by enhancing the level of monoamine neurotransmitters in the CNS [6]. The MAO-B Is selegiline, safinamide, and rasagiline, whose structure is incorporated into that of ladostigil (Figure 1), are used for the treatment of PD [7].

Cholinesterase exists in two isoforms, acetylcholinesterase (AChE) and butyrylcholinesterase (BChE), which catalyze the hydrolysis of acetylcholine (ACh), that activates cholinergic synapses and receptors [8]. The impairment of cholinergic neurotransmission is a downstream process in the cognitive deficit associated with AD. To date, cholinesterase inhibitors (ChE Is) are among the few approved drugs for the symptomatic treatment of AD (Figure 1) that are able to restore cortical cholinergic neurotransmission with consequent beneficial effects on cognition in both AD and PD-related dementia [9]. Among them, rivastigmine, donepezil, and galantamine are currently indicated in patients with mild-to-moderate AD [10,11].

Due to the multifactorial nature of AD, as well as the frequent comorbidity of various neurodegenerative diseases, the design and optimization of multitarget-directed ligands (MTDLs) represents a noteworthy pharmaceutical development strategy [12,13,14,15,16]. In particular, compounds targeting AChE and MAO-B could be useful tools for the treatment of neurodegenerative diseases. The first concrete result was achieved with ladostigil (Figure 1), a dual inhibitor of AChE (reversible) and MAO-B (irreversible), currently in clinical trials (NCT01354691) as an anti-AD drug [17].

Because of the extreme individual variability of the neurodegenerative processes during the different stages of the disease, the search for innovative pharmacological tools that can be used in precision medicine is a real challenge. Controlling the activity of a drug by appropriate external stimuli could represent an effective strategy in the development of “individualizable molecules”. Among the external stimuli, there is growing interest in light, which can affect a tissue with high spatial and temporal precision in a noninvasive and easily controlled manner [18].

In the last decade, the concept of photopharmacology has been widely developed using bioactive molecules that can change their biological effect as a result of light irradiation [18]. In the initial phase, the development of photoactive molecules was based on photolabile functional groups that irreversibly detached from the photodrug and released the bioactive molecule into the irradiated area. Later, with the advent of light-controlled molecular switches (notably azobenzene) aimed at developing more adaptable molecules, photodrug design shifted to the conjugation of bioactive molecules with molecular photoswitches that could change their geometry in response to an appropriate light stimulus and thus, reversibly switch the pharmacological activity on or off [19,20,21].

Several case studies have been proposed for molecules with photomodulable activity against targets such as ion channels [22,23,24], enzymes [25,26], and G-protein-coupled receptors (GPCRs) [19,21,27]. However, despite the crucial role of AChE and MAO-B in the neuronal network, which proceeds with extremely fast kinetics, there are few examples of photomodulable inhibitors for these enzymes [28,29].

In this context, we have combined the expertise in the synthesis and characterization of biomimetic light-controlled molecular switches and motors [30,31,32,33,34,35,36] and in the design of ChE-Is [37,38,39], which have recently led to a small series of photoisomerizable donepezil-like compounds with on-off inhibitory activity towards AChE and MAO-B [40]. These molecules consist of a diversely decorated indanone moiety linked to an N-benzyl-N-ethylethanamine group via an isomerizable carbon-carbon double bond. Their irradiation leads to the spatial repositioning of the key pharmacophore features responsible for the interactions in the binding sites of the target enzymes.

Among these molecules, **1a** (Figure 1), bearing the 5-methoxyindan-1-one moiety, showed the most interesting combination of photophysical and biological profiles. The geometric isomer *E*-**1a** achieved noteworthy inhibitory activity in the high nanomolar range against both enzymes (AChE IC_50_ = 0.113 μM, MAO-B IC_50_ = 0.260 μM), showing selectivity over the BChE and MAO-A isoforms of two orders of magnitude. Irradiation of *E*-**1a** with UV-B light produced an *E/Z* mixture composed of 75% of the *Z*-isomer in the photostationary state (PSS), and the inhibitory effect of *Z*-**1a** on both AChE and MAO-B decreased by one order of magnitude (AChE IC_50_ = 1.15 μM, MAO-B IC_50_ = 1.94 μM), demonstrating off-activity behavior.

Based on these findings, we expand herein the series of 5-methoxyindanone derivatives by synthesizing new compounds in which the tertiary amino residue of 1a bears alkyls with different bulkiness and lipophilicity (Figure 1). A major aim of this study is the investigation of the effect of the amino/ammonium head on the diastereoisomerism-dependent activity by evaluating the difference in the inhibitory capacity of the mixtures at the PSS, which mainly consists of the photoinduced isomer *Z* (75%), on AChE and MAO-B.

## 2. Design and Synthesis

**Figure 2:**
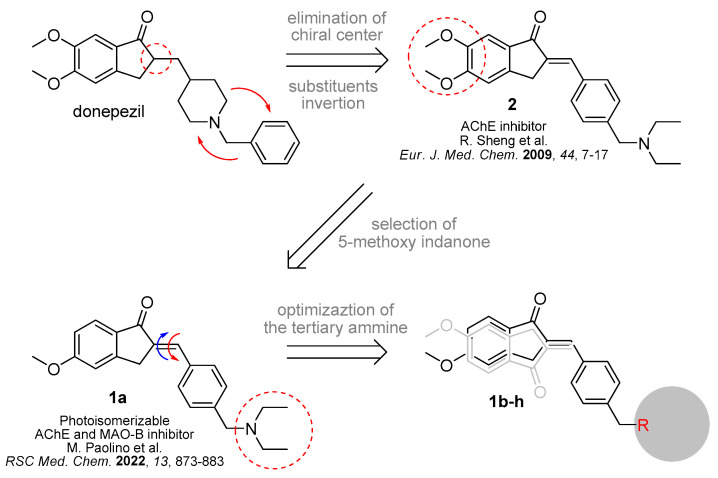
Design of the new photoisomerizable dual AChE/MAO-B inhibitors. Compounds **2** and **1a** have been reported in ref [41] and [40] respectively.

From a pharmacological point of view, the structure of parent compound **1a** can be traced back to the well-known anti-Alzheimer drug donepezil. This molecule has a 5,6-methoxyindanone unit linked to an N-benzylpiperidine moiety via a chiral center. By manipulating this structure, Sheng and coworkers discovered non-chiral compounds with AChE-inhibitory activity comparable to donepezil [41]. Specifically, they replaced the chiral center with an exocyclic double bond and reversed the position of the benzyl ring with the basic center (e.g., compound **2**, Figure 2). In our previous study, focusing on dual AChE/MAO-B inhibitors, the effect of methoxy groups on the interaction with the two enzymes was investigated. We found that removing the 6-OMe group of the indanone moiety resulted in a molecule with submicromolar inhibitory activity for both target enzymes [40]. Thanks to the presence of the hemiindigoid chromophore [42,43], this molecule is able to reversibly photoisomerize by the application of UV light and spatially shift the functional groups responsible for the interaction in the binding sites of the two enzymes. Among the key pharmacophore features, the tertiary amino residue plays a first-rate role by interacting at the catalytic anionic site (CAS) site of AChE, anchoring the inhibitor in the enzyme gorge. Photoisomerization of the exocyclic double bond leads to the displacement of the indanone carbonyl, and the 5-OMe group alters their interactions in the middle of the binding gorge and at the peripheral anionic site (PAS).

In this study, the ethyl residues on the tertiary amine were replaced by various alkyl chains (linear or branched) of different lipophilicity and bulkiness. These substitutions could have favorable or unfavorable effects on the interaction of the tertiary amine in the CAS region of the AChE gorge and, conversely, on other interactions in the middle gorge and in PAS. On the other hand, the increase in lipophilicity around the basic head could improve the inhibition potency against the MAO-B enzyme.

**Scheme 1:**
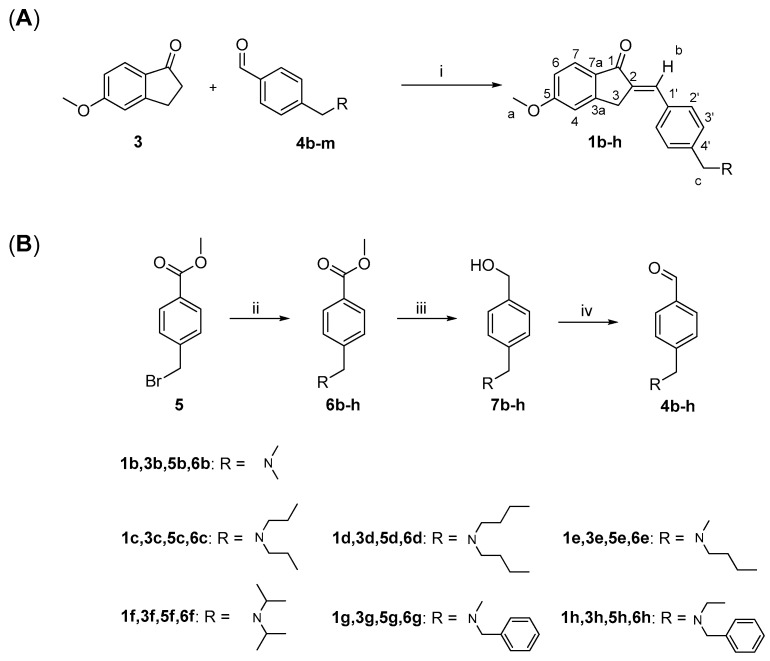
Synthesis of compounds *E*-**1b**–**h** (**A**) and aldehyde derivatives **4b**–**h** (**B**). **Reagents and conditions:** (i) CH_3_OH, KOH, r.t., 2–6 h; (ii) appropriate secondary amine, CH_2_Cl_2_, reflux, 4–5 h; (iii) LiAlH_4_, dry THF, 12 h; (iv) MnO_2_, 1,4 dioxane, reflux, 2–4 h.

The aldehyde derivatives **4b**–**h** were synthesized according to the reactions shown in Scheme 1B. Bromide derivative **5** was reacted with the corresponding secondary amine in DCM at a refluxing temperature. The ester function of the resulting tertiary amines **6b–h** was reduced with LiAlH_4_ in anhydrous THF to afford the primary alcohol derivatives **7b**–**h**, which were oxidized to the corresponding aldehydes **4b–h** with MnO_2_ in refluxing 1,4-dioxane.

Compounds **1b**–**h** were prepared by aldol condensation of 5-methoxyindan-1-one (**3**) with the corresponding aldehyde derivative **4b**–**h** using KOH in methanol, as shown in Scheme 1A. In this reaction, only the *E* diastereoisomer was obtained for each compound, according to the assignment by ^1^H NMR analysis. In the ^1^H NMR spectra of compounds **1b**–**h**, the vinyl proton “b” (see Scheme 1A for numbering) shows a chemical shift of 7.5 ppm, consistent with the deshielding effect of the carbonyl group, as already observed in the spectrum of **1a**.

## 3. Photophysical and Photochemical Characterization

**Figure 3:**
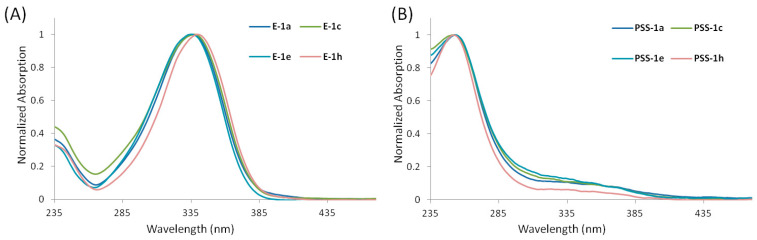
(**A**) Normalized UV-Vis absorption spectra of methanolic solutions of compounds *E-***1a,c,e,h**. (**B**) Normalized UV-Vis absorption spectra of methanolic solution of compound **1a,c,e,h** at PSS generated by UV-B irradiation.

The substitutions at the amino group in compounds **1b**–**h** do not change the structure of the chromophore compared to their precursor **1a**. The absorption spectra registered in methanol at concentrations of about 1 × 10^−5^ M (Figure 3A and Figure S40A in Supplementary Information) are indeed very similar to the UV-vis spectrum of **1a** [40]. For each compound, the absorption spectrum is dominated by an intense peak at about 334–340 nm (with an absorption tail up to about 390 nm) that largely overlaps with the emission spectrum from UV-B lamps. Therefore, this light emission was used for the photoisomerization of the new compounds in ^1^H NMR and UV-Vis absorption experiments.

Using ^1^H NMR spectroscopy, the formation of the *Z* diastereoisomer can be determined with confidence. Specifically, as previously done for **1a**, methanol-d_4_ solutions of compounds **1b**–**e** at a concentration of about 10 mM were irradiated with UV-B light in a Pyrex NMR tube and observed until the photostationary state (PSS) was reached.

**Figure 4:**
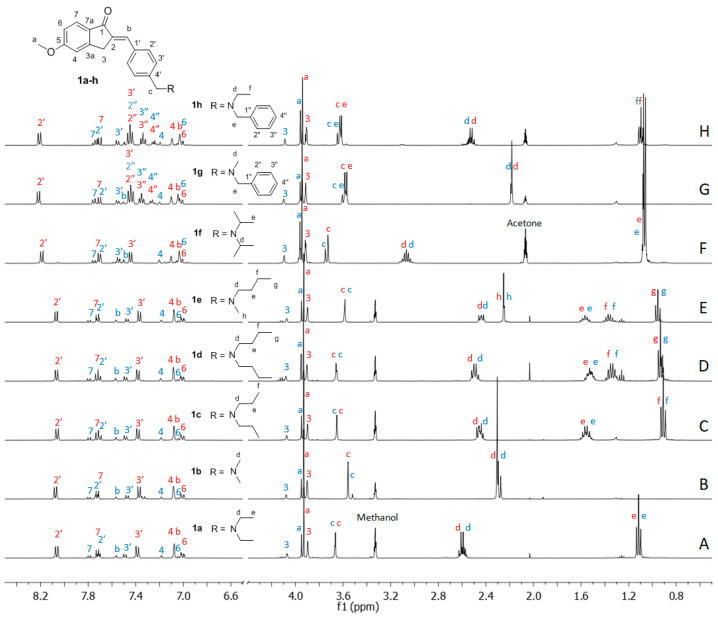
^1^H NMR spectra (400 MHz) of the *E/Z* mixtures obtained at the PSSs after irradiation with UV-B light of compounds **1a**–**e** (spectra A–E) in methanol-d_4_ and of compounds **1f**–**h** (spectra F–H) in acetone-d_6_. Blue numbering was used for assignment of the *E* diastereomers, while red numbering was used for the *Z* isomers.

The same procedure was followed for compounds **1f**–**h**. However, due to the poor solubility in methanol at the concentrations required for ^1^H NMR analysis, **1f**–**h** was irradiated and monitored in an acetone-d_6_ solution. The diastereomeric *E/Z* ratio of the obtained PSS-**1b**–**h** was determined by calculating the area of the easily distinguishable signals assigned to the *E* and *Z* isomers (Figure 4).

In all experiments, the PSS resulting from UV-B irradiation consisted mainly of the *Z* geometric isomer (75%) together with the *E* isomer (25%), confirming that the replacement of the nonconjugated amino group by the hemiindigoid chromophore did not affect the photochemical properties of the new compounds.

**Figure 5:**
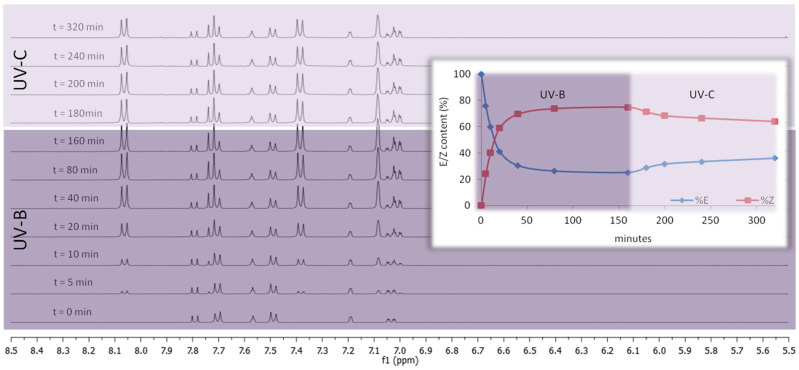
Comparison of the ^1^H NMR spectra (400 MHz, CD_3_OD, range 8.5–5.5 ppm) recorded for monitoring the photoconversion kinetics of compound **1c** (concentration 10 mM). The methanolic solution was irradiated in a Pyrex NMR tube with UV-B light using a multi*-*ray chamber equipped with two tubular lamps *(*15 Watt GT15T8 Hg UV*-*B tube) at a temperature of 24 °C in continuous rotation for 160 min and, then for 160 min with UV*-*C light generated using two tubular lamps (15 Watt GT15T8 Hg UV*-C* tube).

As an example, Figure 5 shows the photoconversion kinetics obtained by irradiating **1c** (10 mM in methanol-d_6_) in a Pyrex NMR tube using a multi-ray chamber equipped with two lamps (15 Watt GT15T8 Hg UV-B tube) at a temperature of 24 °C in continuous rotation. The solution was irradiated with UV-B light until reaching the PSS_UV-B_ (almost completed in 50–80 min). Subsequently, the PSS_UV-B_ solution was irradiated under the same conditions with UV-C light generated using two lamps (15 Watt GT15T8 Hg UV-C tube), observing the photoconversion of the *cis* isomer into the *trans* isomer without the appearance of photodegradation.

All PSS-**1b**–**h** solutions were stored at room temperature in the dark (or even in visible ambient light) for 2 days and reexamined by ^1^H NMR. As with *Z*-**1a**, for which a half-life in MeOH at room temperature of 47 days was calculated [40], no significant change in *E/Z* composition was observed.

The effect of UV-B irradiation was also monitored by UV-Vis absorption spectroscopy (Figure 3B and Figure S40B in Supplementary Information). Methanolic solutions (1 × 10^−5^ M) of **1b**–**h** at the PSS showed a drastic decrease in the absorption bands attributed to the *E* diastereomers and the appearance of new blue-shifted absorption bands attributed to the presence of the *Z* diastereoisomers, consistent with a decrease in π-π electron conjugation [44].

## 4. Biological Evaluation

The pure *E* isomers **1b**–**h**, along with *E*-**1a** retested in this study, and the related *E/Z* mixtures at the PSS (75% *Z* isomer) were evaluated for their inhibitory activity against AChE and BChE, and MAO-A and B. The inhibition data summarized in Table 1 show that all compounds, without any noteworthy structure-dependent difference, are selective toward AChE and MAO-B over BChE and MAO-A, respectively.

ChE inhibitory activities were determined by applying Ellman’s assay with slight modifications, using the drugs donepezil and tacrine as positive controls of AChE and BChE selective inhibition, respectively [45,46]. All the pure geometric isomers *E*-**1a**–**h** selectively inhibit AChE, with IC_50_ values falling in the submicromolar concentration range. Weaker inhibition of BChE was observed for all the compounds, with only *E*-**1f**–**h** achieving finite IC_50_s ≤ 10 μM. *E*-**1h** (IC_50_ = 39 nM) turned out almost equipotent with donepezil (IC_50_ = 21 nM), taken as positive control together with the dual AChE/BChE inhibitor tacrine. All the *E/Z* mixtures (75% *Z*) were found to be less active toward AChE than the corresponding pure *E* isomers, suggesting that the *Z* isomers have a lower binding affinity compared to the *E* isomers, as previously observed for the pure *Z*-**1a** [40].

A pairwise comparison of the AChE inhibition data with the physicochemical features of the tertiary amino head would suggest that the hydrophobicity/bulkiness (not sharply distinguishable within this series) of the -NR_2_ may affect the AChE inhibitory potency of the examined compounds. However, the pure *E* isomers cover an IC_50_ range of just 1.4 log units, and with the exception of the most active (**1h**, R = −N(Et)Bn) and the least active (**1d**, R = −N^n^Bu_2_) inhibitors, the activity data for the other six compounds span a range of only 0.5 log units. Nevertheless, within the limits of the biological and lipophilicity space explored, a nonlinear (likely parabolic) correlation trend (not shown) between −logIC_50_ and the calculated log P for the −NR_2_ fragment appears to hold for six of eight compounds. Two apparent outliers with respect to the parabolic relationship trend were the less-active-than-predicted **1f** (likely, higher steric hindrance of ^i^Pr compared to ^n^Pr groups in **1c**) and the more-active-than-predicted **1h** (additional aromatic interactions of the Bn group compared to Et in **1a**).

**Figure 6:**
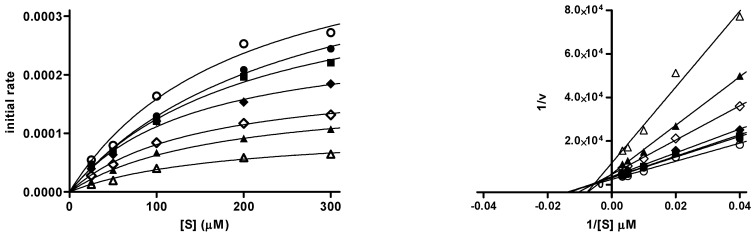
Inhibition kinetics (left) and Lineweaver–Burk plot (right), r^2^ = 0.983–0.999 for hAChE (0.2 U/mL) and *E*-**1h** (0–500 nM), using different substrate (acetylthiocholine iodide) concentrations (50–300 μM). The replot (r^2^ = 0.997) of the slopes versus [I] determined the K_i_ (100 nM) as the x-axis intercept (see Supplementary Information). (○) no inhibitor, (●) 10 nM, (■) 25 nM, (◆) 50 nM, (◇) 100 nM, (▲) 200 nM, (△) 500 nM.

The mechanism of AChE inhibition by the most potent inhibitor *E*-**1h,** was investigated. Lineweaver–Burk curves were generated with a fixed amount of AChE and substrate concentrations ranging between 25 and 300 mM in the absence or presence of the inhibitor at different concentrations (ranging from 5 to 500 nM). The binding of *E*-**1h** to AChE changed both *V_max_* and *K_m_* values, a trend generally attributed to the mixed-type inhibition (Figure 6). The replot of the slopes versus the corresponding inhibitor concentrations provided a *K_i_* value of 100 nM.

Taking into account our previous findings [40] and literature data on structurally similar compounds [47,48], MAO-A/B inhibition was evaluated for pure *E* isomers and *E/Z* mixtures. All the compounds in the pure *E* form proved to be MAO-B-selective inhibitors with all submicromolar IC_50_ values. Only in two cases, namely *E/Z* **1c** (R = −N^n^Pr_2_) and **1f** (R = −N^i^Pr_2_), finite IC_50_ values around 3 μM were achieved against MAO-A, with the *Z* isomers being slightly more potent than the *E* isomers.

The IC_50_ values of MAO-B are in a narrow concentration range (just 0.6 log units) with no apparent lipophilicity-dependent effect. Even in the case of MAO-B inhibition, the *Z* isomers were found to be less potent than the *E* isomers, with the notable exception of **1h** (R = −N(Et)Bn) whose *E/Z* mixture in the PSS (75% *Z*) achieving the same IC_50_ as the pure *E*-**1h**.

Combining the inhibition data on the two enzymes, it is interesting to note that the replacement of the -NEt_2_ group of compound **1a** with a more hindered dissymmetrical substituent as in the case of compound **1e** (R = −N(Me)Bu) led to an improvement in the MAO-B IC_50_ of the diastereoisomer *E* (IC_50_ *E*-**1a** = 0.260 μM vs. *E*-**1e** = 0.178 μM) at the expense of the diastereoisomeric difference (IC_50_ PSS *E/Z*-**1a** = 1.59 μM vs. PSS *E/Z*-**1e** 0.651 μM). In contrast, the inhibitory capacity of *E*-**1e** towards AChE remained almost unchanged with respect to **1a** while significantly increasing the deactivating effect of light (IC_50_ PSS *E/Z*-**1a** = 0.78 μM vs. PSS *E/Z*-**1e** 1.36 μM).

Regarding the potent AChE/MAO-B dual inhibitor **1h**, the *E* isomer is slightly more potent than the *Z* isomer as an AChE inhibitor, whereas no diastereoselectivity was observed for MAO-B inhibition.

## 5. Molecular Docking Analysis

To explain the observed inhibition data of the isomers *E*-**1h** and *Z*-**1h** against human AChE and MAO-B at the molecular level, docking calculations were performed on crystal structures of *h*AChE complexed with donepezil (PDB code: 4EY7) and *h*MAO-B in complex with safinamide (PDB code: 2V5Z). Considering the top-scored docking pose, both *E*-**1h** and *Z*-**1h** diastereoisomers accommodate in the gorge of the active site, in the middle of the PAS and the CAS [49].

**Figure 7:**
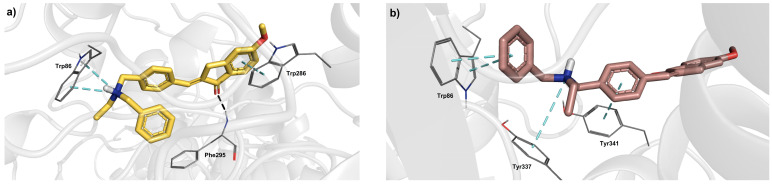
Glide top-ranked docking poses of (**a**) *E*-**1h** (docking score = −13.172 kcal/mol, ligand efficiency = −0.439) and (**b**) *Z*-**1h** (docking score = −11.494 kcal/mol, ligand efficiency = −0.383) in complex with *h*AChE (PDB 4EY7; donepezil: docking score = −13.117 kcal/mol, ligand efficiency = −0.468). The diastereoisomer ligands are depicted as sticks; the interacting residues are rendered as lines, while protein is represented as a cartoon. Dashed black lines represent H-bond; meanwhile, π-π and π-cation interactions are indicated by dashed cyan lines.

**Figure 8:**
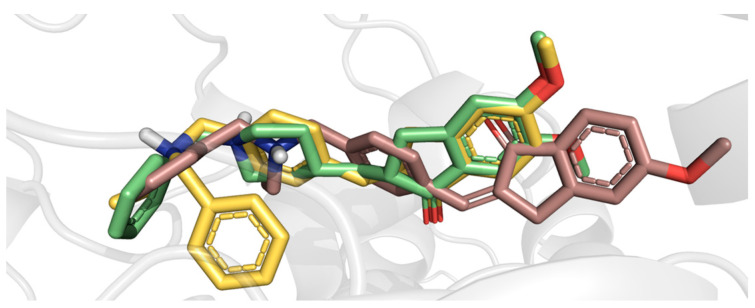
Superimposition of *E*-**1h** (yellow sticks), *Z*-**1h** (violet sticks), and donepezil (green sticks) as docked in the top-scored poses (*E/Z*-**1h**) or established by the X-ray crystal structure (donepezil) within the AChE binding site.

In particular, the indanone group of *E*-**1h**, in its top-scored pose (Figure 7), occupies the PAS by establishing a π–π interaction with W286 and the gorge through a backbone H-bond with F295 mediated by the carbonyl group; additionally, it interacts inside the CAS forming a cation-π interaction, which involves W86 and the protonated tertiary amino group. Otherwise, the **1h** in *Z* geometry locates the indanone core away from the PAS and contacts the CAS through π–π interactions with W86; moreover, the *Z*-isomer engages the aromatic residues of the gorge, and, more specifically, Y341 and Y337 by π–π stacking and cation-π interactions, respectively. More importantly, the docking-based comparison of *E* and *Z* geometries shows that the former best complies with the X-ray donepezil bioactive conformation (Figure 8). The indanone cores of donepezil and *E*-**1h** are well superimposed, and both make interactions with F295 and W286. Furthermore, although lacking the piperidine nitrogen as donepezil, which forms cation-π interaction with Y337, *E*-**1h** has a protonable benzylamino group which may engage double cation-π interactions with W86, as the cation-π interaction and π–π stacking found out in the AChE inhibitor drug. Noteworthy, the higher inhibitor potency of the *E*-**1h** diastereoisomer is in agreement with its higher docking score and ligand efficiency (LE) values.

**Figure 9:**
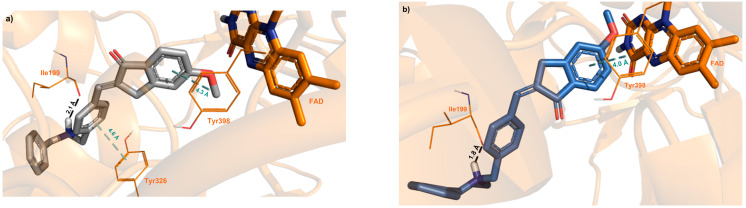
Glide top-ranked docking poses of (**a**) *E*-**1h** (grey stick, docking score = −10.129 kcal/mol, ligand efficiency = −0.338) and (**b**) *Z*-**1h** (blue stick, docking score = −12.256 kcal/mol, ligand efficiency = −0.409) in complex with hMAO-B (PDB 2V5Z; safinamide: docking score = −10.428 kcal/mol, ligand efficiency = −0.474). The diastereoisomer ligands are depicted as sticks; the interacting residues are rendered as lines, while protein is represented as cartoons. Dashed black lines represent H-bonds; meanwhile, π-π and π-cation interactions are represented as dashed cyan lines. FAD coenzyme is shown in orange sticks.

As far as docking calculation on hMAO-B is concerned, the top-scored poses of both *E*-**1h** and *Z*-**1h** diastereoisomers show the indanone moiety facing the FAD moiety (Figure 9). As experienced by the cognate ligand, an H-bond is established between the carbonyl backbone of I199 and the protonated amino, as well as π–π stacking interactions.

Unlike safinamide, the indanone scaffold achieves π–π interaction with Y398 for both the *E* and *Z* isomers, while the conjugated benzyl group engages Y326, an MAO-B selective residue [50] only in the *E* form. In the case of the top-scored docking poses of **1h** complexed with MAO-B, the Glide docking score and the calculated ligand efficiency slightly favors the *Z* isomer over the *E* isomer, likely agreeing with the experimental IC_50_ values, considering that even the PSS *E/Z* mixture containing 75% *Z* isomer showed a similar inhibition potency of the pure *E* isomer.

## 6. Materials and Methods

Synthesis. All chemicals used were of reagent grade. Yields refer to purified products and are not optimized. Merck silica gel 60 (230−400 mesh) was used for column chromatography. Merck TLC plates and silica gel 60 F254 were used for TLC. NMR spectra were obtained with a Bruker 400 AVANCE spectrometer in the indicated solvents. Melting points were determined in open capillaries in a Gallenkamp apparatus and were uncorrected. The chemical shifts are referenced to the residual not deuterated solvent signal (CHD_2_OD: δ (^1^H) = 3.31 ppm, δ (^13^C) = 49.86 ppm). The values of the chemical shifts are expressed in ppm, and the coupling constants (*J*) in Hz. An Agilent 1100 LC/MSD operating with an electrospray source was used in mass spectrometry experiments. The purity of compounds **1b**–**h** was assessed by RP-HPLC (Agilent 1100 series) and was found to be higher than 95% [40]. A Zorbax Eclipse XDB-C8 column (4.6 × 150 mm, 5 μm) was used in the HPLC analysis with methanol-H_2_O (0.1% formic acid) (80:20) as the mobile phase at a flow rate of 0.5 mL/min. UV detection was achieved at 280 nm. The absorption spectra were recorded with a PerkinElmer Lambda 900 in the indicated solvent. UV-B irradiations were conducted using a Multyrays chamber equipped with 2 GT15T8 Hg UV-B tubes (2 × 15 Watt) in continuous rotation.

### 6.1. Chemistry

#### 6.1.1. General Procedure for the Synthesis of Compounds **1b**–**h**

Compounds **1b**–**h** were prepared by optimizing a previously reported procedure [41]. To a solution, 5-methoxy indanone (1 eq.) in methanol (5 mL/mmol), KOH (1 eq.), and the appropriate aldehyde **4b**–**h** (1 eq.) were added. The resulting mixture was stirred at room temperature under a nitrogen atmosphere for 2–6 h. Subsequently, the solvent was removed under reduced pressure to obtain a solid residue, which was dissolved in ethyl acetate and washed with brine. The organic phase was dried over anhydrous sodium sulfate, filtered, and concentrated under reduced pressure. The residue was purified by flash chromatography with the indicated solvent as the eluent to afford an off-white solid corresponding to the desired compound **1b**–**h** as *E* diastereoisomer. NMR data of the Z diastereoisomers of each compound were derived after UV-B light irradiation of the corresponding *E* diastereoisomer until the PSS.

(*E*)-2-(4-((Dimethylamino)methyl)benzylidene)-5-methoxy-2,3-dihydro-1*H*-inden-1-one (*E*-**1b**).

Compound *E-***1b** (0.058 g, yield 53%, m.p. 122–123 °C) was obtained as a pale yellow solid from aldehyde **4b** (0.06 g, 0.36 mmol) after purification with a mixture of ethyl acetate/methanol (9:1) as the eluent. ^1^H NMR (400 MHz, CD_3_OD): 2.29 (s, 6H), 3.56 (s, 2H), 3.96 (s, 3H), 4.09 (s, 2H), 7.04 (dd, *J* = 8.6, 2.3, 1H), 7.20 (d, *J* = 2.0, 1H), 7.48 (d, *J* = 8.2, 2H), 7.58 (t, *J* = 2.0, 1H), 7.74 (d, *J* = 8.2, 2H), 7.80 (d, *J* = 8.6, 1H). ^13^C NMR (400 MHz, CD_3_OD): 32.0, 43.9, 55.0, 63.2, 109.4, 115.5, 125.5, 129.9, 130.4, 130.6, 132.3, 134.5, 135.4, 139.4, 153.6, 166.1, 193.6. MS(ESI): *m/z* 308.1 [M + H]^+^.

(*Z*)-2-(4-((Dimethylamino)methyl)benzylidene)-5-methoxy-2,3-dihydro-1*H*-inden-1-one (*Z*-**1b**).

^1^H NMR (400 MHz, CD_3_OD): 2.31 (s, 6H), 3.56 (s, 2H), 3.90 (s, 2H), 3.93 (s, 3H), 7.01 (dd, *J* = 8.6, 2.3, 1H), 7.08 (m, 2H), 7.37 (d, *J* = 8.2, 2H), 7.72 (d, *J* = 8.6, 1H), 8.07 (d, *J* = 8.2, 2H).

(*E*)-2-(4-((Dipropylamino)methyl)benzylidene)-5-methoxy-2,3-dihydro-1*H*-inden-1-one (*E*-**1c**).

Compound *E-***1c** (0.12 g, yield 60%, m.p. 73.5–75.5 °C) was obtained as a pale yellow solid from aldehyde **4c** (0.12 g, 0.55 mmol) after purification with a mixture of petroleum ether/ethyl acetate (1:1) as the eluent. ^1^H NMR (400 MHz, CD_3_OD): 0.90 (t, *J* = 7.4, 6H), 1.55 (m, 4H), 2.44 (t, *J* = 7.6, 4H), 3.64 (s, 2H), 3.94 (s, 3H), 4.05 (s, 2H), 7.02 (dd, *J* = 8.5, 2.2, 1H), 7.17 (d, *J* = 2.0, 1H), 7.48 (d, *J* = 8.2, 2H), 7.55 (t, *J* = 1.8, 1H), 7.69 (d, *J* = 8.2, 2H), 7.78 (d, *J* = 8.5, 1H). ^13^C NMR (100 MHz, CD_3_OD): 10.8, 19.5, 32.0, 55.0, 55.7, 58.0, 109.4, 115.5, 125.4, 129.4, 130.3, 130.6, 132.6, 134.0, 135.0, 141.4, 153.6, 166.0, 193.7. MS(ESI): *m/z* 364.2 [M + H]^+^.

(*Z*)-2-(4-((Dipropylamino)methyl)benzylidene)-5-methoxy-2,3-dihydro-1*H*-inden-1-one (*Z*-**1c**).

^1^H NMR (400 MHz, CD_3_OD): 0.91 (t, *J* = 7.4, 6H), 1.55 (m, 4H), 2.46 (t, *J* = 7.6, 4H), 3.65 (s, 2H), 3.90 (s, 2H), 3.93 (s, 3H), 7.01 (dd, *J* = 8.6, 2.2, 1H), 7.08 (m, 2H), 7.38 (d, *J* = 8.2, 2H), 7.72 (d, *J* = 8.7, 1H), 8.06 (d, *J* = 8.2, 2H).

(*E*)-2-(4-((Dibutylamino)methyl)benzylidene)-5-methoxy-2,3-dihydro-1*H*-inden-1-one (*E*-**1d**).

Compound *E-***1d** (0.04 g, yield 64%, m.p. 74.5–75.5 °C) was obtained as a pale yellow solid from aldehyde **4d** (0.04 g, 0.16 mmol) after purification with a mixture of petroleum ether/ethyl acetate (2:1) as the eluent. ^1^H NMR (400 MHz, CD_3_OD): 0.93 (t, *J* = 7.3, 6H), 1.34 (m, 4H), 1.52 (m, 4H), 2.48 (t, *J* = 7.5, 4H), 3.64 (s, 2H), 3.95 (s, 3H), 4.08 (s, 2H), 7.03 (dd, *J* = 8.5, 2.2, 1H), 7.19 (d, *J* = 1.9, 1H), 7.48 (d, *J* = 8.1, 2H), 7.56 (s, 1H), 7.70 (d, *J* = 8.2, 2H), 7.79 (d, *J* = 8.6, 1H). ^13^C NMR (400 MHz, CD_3_OD): 13.0, 20.3, 28.6, 32.0, 53.3, 55.0, 58.0, 109.4, 115.5, 125.4, 129.5, 130.3, 130.67, 132.6, 134.0, 135.0, 143.6, 153.6, 166.0, 193.7. MS(ESI): *m/z* 392.2 [M + H]^+^.

(*Z*)-2-(4-((Dibutylamino)methyl)benzylidene)-5-methoxy-2,3-dihydro-1*H*-inden-1-one (*Z*-**1d**).

^1^H NMR (400 MHz, CD_3_OD): 2.31 (s, 6H), 3.56 (s, 2H), 3.90 (s, 2H), 3.93 (s, 3H), 7.01 (dd, *J* = 8.6, 2.3, 1H), 7.08 (m, 2H), 7.37 (d, *J* = 8.2, 2H), 7.72 (d, *J* = 8.6, 1H), 8.07 (d, *J* = 8.2, 2H).

(*E*)-2-(4-((Butyl(methyl)amino)methyl)benzylidene)-5-methoxy-2,3-dihydro-1*H*-inden-1-one (*E*-**1e**).

Compound *E-***1e** (0.071 g, yield 79%, m.p. 62.4–63.0 °C) was obtained as a pale yellow solid from aldehyde **4e** (0.05 g, 0.26 mmol) after purification with a mixture of petroleum ether/ethyl acetate (1:1) as the eluent. ^1^H NMR (400 MHz, CD_3_OD): 0.95 (t, *J* = 7.4, 3H), 1.35 (m, 2H), 1.56 (m, 2H), 2.24 (s, 3H), 2.43 (t, *J* = 7.8, 2H), 3.58 (s, 2H), 3.95 (s, 3H), 4.06 (s, 2H), 7.03 (dd, *J* = 8.6, 2.2, 1H), 7.18 (d, *J* = 2.0, 1H), 7.47 (d, *J* = 8.2, 2H), 7.55 (t, *J* = 1.9, 1H), 7.71 (d, *J* = 8.2, 2H), 7.78 (d, *J* = 8.6, 1H). ^13^C NMR (100 MHz, CD_3_OD): 12.9, 20.3, 28.8, 32.0, 41.1, 55.0, 56.9, 61.4, 109.4, 115.5, 125.5, 129.8, 130.4, 130.6, 132.4, 134.3, 135.3, 140.0, 153.6, 166.1, 193.6. MS(ESI): *m/z* 350.2 [M + H]^+^.

(*Z*)-2-(4-((Butyl(methyl)amino)methyl)benzylidene)-5-methoxy-2,3-dihydro-1*H*-inden-1-one (*Z*-**1e**).

^1^H NMR (400 MHz, CD_3_OD): 0.95 (t, *J* = 7.4, 3H), 1.36 (m, 2H), 1.57 (m, 2H), 2.25 (s, 3H), 2.44 (t, *J* = 7.8, 2H), 3.59 (s, 2H), 3.90 (s, 2H), 3.93 (s, 3H), 7.01 (dd, *J* = 8.6, 2.3, 1H), 7.08 (m, 2H), 7.37 (d, *J* = 8.2, 2H), 7.72 (d, *J* = 8.6, 1H), 8.07 (d, *J* = 8.2, 2H).

(*E*)-2-(4-((Diisopropylamino)methyl)benzylidene)-5-methoxy-2,3-dihydro-1*H*-inden-1-one (*E*-**1f**).

Compound *E-***1f** (0.022 g, yield 45%, m.p. 74.5–75.5 °C) was obtained as an off-white solid from aldehyde **4f** (0.03 g, 0.14 mmol) after purification with a mixture of petroleum ether/ethyl acetate (8:2) as the eluent. ^1^H NMR (400 MHz, (CD_3_)_2_CO): 1.07 (d, *J* = 6.6, 12H), 3.07 (m, 2H), 3.75 (s, 2H), 3.96 (s, 3H), 4.09 (s, 2H), 7.04 (dd, *J* = 8.5, 2.2, 1H), 7.20 (d, *J* = 1.9, 1H), 7.50 (t, *J* = 2.0, 1H), 7.54 (d, *J* = 8.2, 2H), 7.71 (d, *J* = 8.2, 2H), 7.75 (d, *J* = 8.5, 1H). ^1^H NMR (400 MHz, (CD_3_)_2_SO): 1.01 (d, *J* = 6.6 Hz, 12H), 3.04–2.87 (m, 2H), 3.67 (s, 2H), 3.90 (s, 3H), 4.07 (s, 2H), 7.04 (dd, *J* = 8.5, 2.2 Hz, 1H), 7.19 (d, *J* = 1.9 Hz, 1H), 7.44 (s, 1H), 7.48 (d, *J* = 8.1 Hz, 1H), 7.69 (d, *J* = 8.2 Hz, 1H), 7.72 (t, *J* = 6.0 Hz, 1H). ^13^C NMR (100 MHz, (CD_3_)_2_SO): 21.1, 32.5, 48.1, 48.7, 56.3, 110.7, 115.9, 125.9, 128.6, 130.9, 131.1, 132.1, 133.6, 135.2, 145.7, 153.4, 165.4, 192.1. MS(ESI): *m/z* 364.2 [M + H]^+^.

(*Z*)-2-(4-((Diisopropylamino)methyl)benzylidene)-5-methoxy-2,3-dihydro-1*H*-inden-1-one (*Z*-**1f**).

^1^H NMR (400 MHz, (CD_3_)_2_CO): 1.07 (d, *J* = 6.6, 12H), 3.07 (m, 2H), 3.73 (s, 2H), 3.91 (s, 2H), 3.95 (s, 3H), 6.99–7.06 (m, 2H), 7.10 (d, *J* = 1.9, 1H), 7.44 (d, *J* = 8.2, 2H), 7.71 (d, *J* = 8.6, 1H), 8.19 (d, *J* = 8.2, 2H).

(*E*)-2-(4-((Benzyl(methyl)amino)methyl)benzylidene)-5-methoxy-2,3-dihydro-1*H*-inden-1-one (*E*-**1g**).

Compound *E-***1g** (0.055 g, yield 62%, m.p. 126.5–127.5 °C) was obtained as an off-white solid from aldehyde **4g** (0.06 g, 0.23 mmol) after purification with a mixture of petroleum ether/ethyl acetate (1:1) as the eluent. ^1^H NMR (400 MHz, (CD_3_)_2_CO): 2.19 (s, 3H), 3.58 (s, 2H), 3.60 (s, 2H), 3.96 (s, 3H), 4.10 (s, 2H), 7.04 (dd, *J* = 8.5, 2.2, 1H), 7.20 (d, *J* = 1.9 1H), 7.27 (t, *J* = 7.3, 1H), 7.35 (t, *J* = 7.5, 2H), 7.43 (d, *J* = 7.4, 2H), 7.50 (t, *J* = 2.0, 1H), 7.55 (d, *J* = 8.1, 2H), 7.75 (d, *J* = 8.2, 3H). ^1^H NMR (400 MHz, (CD_3_)_2_SO): 2.11 (s, 3H), 3.53 (s, 2H), 3.56 (s, 2H), 3.90 (s, 3H), 4.08 (s, 2H), 7.04 (dd, *J* = 8.5, 1.9 Hz, 1H), 7.19 (s, 1H), 7.26 (t, *J* = 6.5 Hz, 1H), 7.42–7.30 (m, 4H), 7.45 (s, 1H), 7.49 (d, *J* = 8.0 Hz, 2H), 7.74 (m, 3H). ^13^C NMR (100MHz, (CD_3_)_2_SO): 32.5, 42.2, 56.3, 61.1, 61.5, 110.7, 115.9, 125.9, 127.4, 128.7, 129.1, 129.6, 131.1, 131.8, 133.4, 134.2, 135.7, 139.5, 141.5, 153.4, 165.4, 192.1. MS(ESI): *m/z* 384.2 [M + H]^+^.

(*Z*)-2-(4-((Benzyl(methyl)amino)methyl)benzylidene)-5-methoxy-2,3-dihydro-1*H*-inden-1-one (*Z*-**1g**).

^1^H NMR (400 MHz, (CD_3_)_2_CO): 2.18 (s, 3H), 3.57 (s, 2H), 3.59 (s, 2H), 3.91 (s, 2H), 3.94 (s, 3H), 7.02 (dd, *J* = 8.5, 2.2, 1H), 7.04 (s, 1H), 7.10 (s, 1H), 7.26 (t, *J* = 7.3, 1H), 7.35 (t, *J* = 7.5, 2H), 7.44 (m, 4H), 7.70 (d, *J* = 8.5, 1H), 8.22 (d, *J* = 8.2, 2H).

(*E*)-2-(4-((Benzyl(ethyl)amino)methyl)benzylidene)-5-methoxy-2,3-dihydro-1*H*-inden-1-one (*E*-**1h**).

Compound *E-***1h** (0.06 g, yield 50%, m.p. 118.5–119.5 °C) was obtained as an off-white solid from aldehyde **4h** (0.07 g, 0.28 mmol) after purification with a mixture of petroleum ether/ethyl acetate (8:2) as the eluent. ^1^H NMR (400 MHz, ((CD_3_)_2_CO): 1.10 (t, *J* = 7.1, 3H), 2.53 (q, *J* = 7.1, 2H), 3.62 (s, 2H), 3.64 (s, 2H), 3.96 (s, 3H), 4.09 (s, 2H), 7.04 (dd, *J* = 8.5, 2.1, 1H), 7.19 (d, *J* = 2.0, 1H), 7.25 (t, *J* = 7.3, 1H), 7.34 (t, *J* = 7.5, 2H), 7.44 (d, *J* = 7.4, 2H), 7.49 (s, 1H), 7.55 (d, *J* = 8.1, 2H), 7.73 (d, *J* = 8.1, 2H), 7.75 (d, *J* = 8.2, 1H). ^1^H NMR (400 MHz, ((CD_3_)_2_SO): 1.03 (t, *J* = 7.1 Hz, 3H), 2.45 (q, *J* = 7.1 Hz, 2H), 3.57 (s, 2H), 3.60 (s, 2H), 3.90 (s, 3H), 4.08 (s, 2H), 7.04 (dd, *J* = 8.5, 2.3 Hz, 1H), 7.19 (d, *J* = 2.0 Hz, 1H), 7.25 (ddd, *J* = 8.5, 3.0, 1.5 Hz, 1H), 7.31–7.41 (m, 4H), 7.45 (s, 1H), 7.49 (d, *J* = 8.1 Hz, 2H), 7.73 (d, *J* = 8.4 Hz, 3H). ^13^C NMR (400 MHz, (CD_3_)_2_SO): 12.1, 32.5, 47.0, 56.3, 57.2, 57.5, 110.7, 115.9, 125.9, 127.3, 128.7, 128.9, 129.4, 131.0, 131.1, 131.9, 134.1, 135.6, 140.0, 142.2, 153.4, 165.4, 192.1. MS(ESI): *m/z* 398.2 [M + H]^+^.

(*Z*)-2-(4-((Benzyl(ethyl)amino)methyl)benzylidene)-5-methoxy-2,3-dihydro-1*H*-inden-1-one (*Z*-**1h**).

^1^H NMR (400 MHz, (CD_3_)_2_CO): 1.10 (t, *J* = 7.1, 3H), 2.52 (q, *J* = 7.1, 2H), 3.61 (s, 2H), 3.63 (s, 2H), 3.91 (s, 2H), 3.94 (s, 3H), 6.99–7.04 (m, 1H), 7.10 (d, *J* = 2.0, 1H), 7.24 (t, *J* = 7.3, 1H), 7.34 (t, *J* = 7.5, 2H), 7.45 (m, 3H), 7.70 (d, *J* = 8.5, 1H), 8.21 (d, *J* = 8.2, 2H).

#### 6.1.2. General Procedure for the Synthesis of Compounds **6b**–**h**

To a solution of ester **5** (1 equivalent) in dichloromethane (10 mL/mmol), the appropriate secondary amine (3 equivalents) was added. The mixture was stirred at a refluxed temperature for 4–5 h. Then, the solvent was removed under reduced pressure and the resulting oily residue was diluted with dichloromethane and washed with water. The organic phase was dried over anhydrous sodium sulfate, filtered, and concentrated under reduced pressure. The resulting oily residue was purified by flash chromatography using the indicated solvent as the eluent to obtain the desired tertiary amine derivatives **6b**–**h**.

Methyl 4-((dimethylamino)methyl)benzoate (**6b**) [41]

Compound **6b** was obtained as a yellow oil (0.42 g, yield 98%) by using a 1 M solution of dimethyl amine in THF (6.6 mL, 6.60 mmol) after purification with a mixture of petroleum ether/ethyl acetate (1:1) as the eluent. ^1^H NMR (400 MHz, CD_3_OD): 2.28 (s, 6H), 3.57 (s, 2H), 3.92 (s, 3H), 7.46 (d, *J* = 8.4, 2H), 8.01 (d, *J* = 8.4, 2H). MS(ESI): *m/z* 194.1 [M + H]^+^.

Methyl 4-((dipropylamino)methyl)benzoate (**6c**) [51]

Compound **6c** was obtained as a yellow oil (0.54 g, yield 99%) by using dipropyl amine (0.9 mL, 6.51 mmol) after purification with a mixture of petroleum ether/ethyl acetate (8:2) as the eluent. ^1^H NMR (400 MHz, CD_3_OD): 0.89 (t, *J* = 7.4, 6H), 1.53 (m, 4H), 2.43 (t, *J* = 7.5, 4H), 3.67 (s, 2H), 3.92 (s, 3H), 7.48 (d, *J* = 8.3, 2H), 7.98 (d, *J* = 8.3, 2H). MS (ESI): *m/z* 250.1 [M + H]^+^.

Methyl 4-((dibutylamino)methyl)benzoate (**6d**) [52]

Compound **6d** was obtained as a yellow oil (0.59 g, yield 94%) by using dibutyl amine (1.1 mL, 6.81 mmol) after purification with a mixture of petroleum ether/ethyl acetate (9:1) as the eluent. ^1^H NMR (400 MHz, CD_3_OD): 0.91 (t, *J* = 7.3, 6H), 1.33 (m, 4H), 1.49 (m, 4H), 2.45 (t, *J* = 7.5, 4H), 3.64 (s, 2H), 3.91 (s, 3H), 7.47 (d, *J* = 8.3, 2H), 7.98 (d, *J* = 8.3, 2H). MS(ESI): *m/z* 278.2 [M + H]^+^.

Methyl 4-((butyl(methyl)amino)methyl)benzoate (**6e**).

Compound **6e** was obtained as a yellow oil (0.51 g, yield 99%) by using N-methylbutan-1-amine (0.8 mL, 6.50 mmol) after purification with a mixture of petroleum ether/ethyl acetate (1:1) as the eluent. ^1^H NMR (400 MHz, CD_3_OD): 0.94 (t, *J* = 7.4, 3H), 1.35 (m, 2H), 1.55 (m, 2H), 2.22 (s, 3H), 2.41 (t, *J* = 7.7, 2H), 3.59 (s, 2H), 3.92 (s, 3H), 7.46 (d, *J* = 8.1, 2H), 8.00 (d, *J* = 8.2, 2H). ^13^C NMR (100 MHz, CD_3_OD): 13.0, 20.3, 28.9, 41.1, 51.2, 56.9, 61.4, 128.9, 129.1 (two close peaks), 144.0, 167.0. MS(ESI): *m/z* 236.1 [M + H]^+^.

Methyl 4-((diisopropylamino)methyl)benzoate (**6f**) [53]

Compound **6f** was obtained as a yellow oil (0.33 g, yield 60%) by using diisopropyl amine (0.9 mL, 6.53 mmol) after purification with petroleum ether as the eluent. ^1^H NMR (400 MHz, CD_3_OD): 1.08 (d, *J* = 6.6, 12H), 3.07 (m, 2H), 3.75 (s, 2H), 3.91 (s, 3H), 7.51 (d, *J* = 8.6, 2H), 7.94 (d, *J* = 8.4, 2H). MS(ESI): *m/z* 250.1 [M + H]^+^.

Methyl 4-((benzyl(methyl)amino)methyl)benzoate (**6g**) [54]

Compound **6g** was obtained as a yellow oil (0.58 g, yield 99%) by using N-methyl-1-phenylmethanamine (0.8 mL, 6.54 mmol) after purification with a mixture of petroleum ether/ethyl acetate (9:1) as the eluent. ^1^H NMR (400 MHz, CD_3_OD): 2.19 (s, 3H), 3.56 (s, 2H), 3.60 (s, 2H), 3.91 (s, 3H), 7.27 (m, 1H), 7.35 (m, 4H), 7.50 (d, *J* = 8.4, 2H), 8.00 (d, *J* = 8.3, 2H). MS(ESI): *m/z* 270.1 [M + H]^+^.

Methyl 4-((benzyl(ethyl)amino)methyl)benzoate (**6h**).

Compound **6h** was obtained as a yellow oil (0.61 g, yield 99%) by using N-ethyl-1-phenylmethanamine (1.0 mL, 6.60 mmol) after purification with a mixture of petroleum ether/ethyl acetate (9:1) as the eluent. ^1^H NMR (400 MHz, CD_3_OD): 1.09 (t, *J* = 7.1, 3H), 2.50 (q, *J* = 7.1, 2H), 3.57 (s, 2H), 3.62 (s, 2H), 3.90 (s, 3H), 7.23 (t, *J* = 7.2, 1H), 7.31 (t, *J* = 7.3, 2H), 7.37 (d, *J* = 7.9, 2H), 7.48 (d, *J* = 8.1, 2H), 7.96 (d, *J* = 8.3, 2H). ^13^C NMR (100 MHz, CD_3_OD): 10.8, 47.0, 51.1, 57.1, 57.6, 126.6, 127.9, 128.5, 128.6, 128.7, 129.1, 139.2, 145.7, 167.1. MS(ESI): *m/z* 284.1 [M + H]^+^.

#### 6.1.3. General Procedure for the Synthesis of the Alcohol Derivatives **7b**–**h**

To a solution of the appropriate ester **6b**–**h** (1 equivalent) in dry THF (5 mL/mmol), a 1 M solution of LiAlH_4_ in THF (1 equivalent) was added. The reaction mixture was stirred at room temperature under a nitrogen atmosphere for 12 h and, subsequently, was concentrated under reduced pressure. The oily residue was dissolved in dichloromethane and then washed with water. The organic phase was dried over anhydrous sodium sulfate, filtered, and concentrated under reduced pressure. The resulting oily residue was purified by flash chromatography using the indicated eluent to obtain the desired alcohol derivatives **7b**–**h**.

(4-((Dimethylamino)methyl)phenyl)methanol (**7b**) [55]

Compound **7b** was obtained as a yellow oil (0.37 g, yield 82%) starting from ester **6b** (0.52 g, 2.71 mmol) after purification with ethyl acetate as the eluent. ^1^H NMR (400 MHz, CD_3_OD): 2.26 (s, 6H), 3.49 (s, 2H), 4.63 (s, 2H), 7.34 (m, 4H). MS(ESI): *m/z* 194.1 [M + H]^+^.

(4-((Dipropylamino)methyl)phenyl)methanol (**7c**).

Compound **7c** was obtained as a yellow oil (0.29 g, yield 67%) starting from ester **6c** (0.49 g, 1.98 mmol) after purification with a mixture of petroleum ether/ethyl acetate (8:2) as the eluent. ^1^H NMR (400 MHz, CD_3_OD): 0.88 (t, *J* = 7.4, 6H), 1.53 (m, 4H), 2.41 (t, *J* = 7.6, 4H), 3.59 (s, 2H), 4.60 (s, 2H), 7.32 (m, 4H). ^13^C NMR (100 MHz, CD_3_OD): 10.9, 19.4, 55.5, 57.9, 63.7, 126.6, 129.0, 137.9, 140.1. MS(ESI): *m/z* 222.2 [M + H]^+^.

(4-((Dibutylamino)methyl)phenyl)methanol (**7d**).

Compound **7d** was obtained as a yellow oil (0.16 g, yield 33%) starting from ester **6d** (0.55 g, 1.98 mmol) after purification with a mixture of petroleum ether/ethyl acetate (8:2) as the eluent. ^1^H NMR (400 MHz, CD_3_OD): 0.92 (t, *J* = 7.3, 6H), 1.31 (m, 4H), 1.50 (m, 4H), 2.44 (t, *J* = 7.6, 4H), 3.59 (s, 2H), 4.61 (s, 2H), 7.32 (m, 4H). ^13^C NMR (100 MHz, CD_3_OD): 13.0, 20.3, 28.4, 53.1, 57.9, 63.7, 126.6, 129.0, 137.8, 140.1. MS(ESI): *m/z* 250.2 [M + H]^+^.

(4-((Butyl(methyl)amino)methyl)phenyl)methanol (**7e**).

Compound **7e** was obtained as a yellow oil (0.40 g, yield 83%) starting from ester **6e** (0.55 g, 2.35 mmol) after purification with a mixture of petroleum ether/ethyl acetate (8:2) as the eluent. ^1^H NMR (400 MHz, CD_3_OD): 0.94 (t, *J* = 7.4, 3H), 1.34 (m, 2H), 1.54 (m, 2H), 2.21 (s, 3H), 2.40 (t, *J* = 7.8, 2H), 3.54 (s, 2H), 4.62 (s, 2H), 7.34 (m, 4H). ^13^C NMR (100 MHz, CD_3_OD): 13.0, 20.4, 28.7, 41.0, 56.7, 61.4, 63.6, 126.6, 129.4, 136.5, 140.5. MS(ESI): *m/z* 208.1 [M + H]^+^.

(4-((Diisopropylamino)methyl)phenyl)methanol (**7f**).

Compound **7f** was obtained as a yellow oil (0.09 g, yield 31%) starting from ester **6f** (0.33 g, 1.32 mmol) after purification with a mixture of petroleum ether/ethyl acetate (9:1) as the eluent. ^1^H NMR (400 MHz, CD_3_OD): 1.06 (d, *J* = 6.6, 12H), 3.05 (m, 2H), 3.67 (s, 2H), 4.58 (s, 2H), 7.28 (d, *J* = 8.1, 2H), 7.36 (d, *J* = 8.2, 2H). ^13^C NMR (100 MHz, CD_3_OD): 19.7, 47.7 (overlapped to methanol), 48.4, 63.8, 126.5, 127.3, 139.2, 141.9. MS(ESI): *m/z* 222.1 [M + H]^+^.

(4-((Benzyl(methyl)amino)methyl)phenyl)methanol (**7g**).

Compound **7g** was obtained as a yellow oil (0.38 g, yield 80%) starting from ester **6g** (0.53 g, 1.98 mmol) after purification with a mixture of petroleum ether/ethyl acetate (8:2) as the eluent. ^1^H NMR (400 MHz, CD_3_OD): 2.18 (s, 3H), 3.53 (s, 2H), 3.54 (s, 2H), 4.61 (s, 2H), 7.28–7.38 (m, 9H). ^13^C NMR (100 MHz, CD_3_OD): 41.0, 61.1, 61.2, 63.6, 126.7, 126.7, 127.9, 129.0, 129.1, 137.3, 138.3, 140.4. MS(ESI): *m/z* 242.1 [M + H]^+^.

(4-((Benzyl(ethyl)amino)methyl)phenyl)methanol (**7h**).

Compound **7h** was obtained as a yellow oil (0.49 g, yield 98%) starting from ester **6h** (0.56 g, 1.98 mmol) after purification with a mixture of petroleum ether/ethyl acetate (8:2) as the eluent. ^1^H NMR (400 MHz, CD_3_OD): 1.09 (t, *J* = 7.1, 3H), 2.50 (q, *J* = 7.1, 2H), 3.56 (s, 2H), 3.57 (s, 2H), 4.60 (s, 2H), 7.23 (t, *J* = 7.1, 1H), 7.33 (m, 8H). ^13^C NMR (100 MHz, CD_3_OD): 10.7, 46.6, 57.1, 57.3, 63.7, 126.6, 126.7, 127.8, 128.7, 128.8, 138.4, 139.3, 140.0. MS(ESI): *m/z* 256.2 [M + H]^+^.

#### 6.1.4. General Procedure for the Synthesis of Aldehyde Derivatives **4b**–**h**

To a solution of the appropriate alcohol derivative **7b**–**h** (1 equivalent) in 1,4-dioxane (3 mL/mmol), MnO_2_ (10 equivalents) was added. The reaction mixture was stirred at reflux temperature for 2–4 h and, subsequently, was filtered on celite. The filtrate was concentrated under reduced pressure, and the resulting residue was purified by flash chromatography with the indicated solvent to give the desired aldehyde derivatives **4b**–**h**.

4-((Dimethylamino)methyl)benzaldehyde (**4b**) [55]

Compound **4b** was obtained as a pale yellow oil (0.09 g, yield 24%) starting from alcohol derivative **7b** (0.37 g, 2.22 mmol) and was purified using ethyl acetate as the eluent. ^1^H NMR (400 MHz, CD_3_OD): 2.26 (s, 6H), 3.58 (s, 2H), 7.56 (d, *J* = 8.1, 2H), 7.91 (d, *J* = 8.2, 2H), 10.0 (s, 1H). MS(ESI): *m/z* 164.1 [M + H]^+^.

4-((Dipropylamino)methyl)benzaldehyde (**4c**).

Compound **4c** was obtained as a pale yellow oil (0.15 g, yield 62%) starting from alcohol derivative **7c** (0.25 g, 1.12 mmol) and was purified using a mixture of petroleum ether/ethyl acetate (1:1) as the eluent. ^1^H NMR (400 MHz, CD_3_OD): 0.90 (t, *J* = 7.4, 6H), 1.54 (m, 4H), 2.43 (t, *J* = 7.5, 4H), 3.69 (s, 2H), 7.58 (d, *J* = 8.1, 2H), 7.88 (d, *J* = 8.2, 2H), 9.98 (s, 1H). MS(ESI): *m/z* 220.1 [M + H]^+^.

4-((Dibutylamino)methyl)benzaldehyde (**4d**).

Compound **4d** was obtained as a pale yellow oil (0.04 g, yield 36%) starting from alcohol derivative **7d** (0.12 g, 0.48 mmol) and was purified using a mixture of petroleum ether/ethyl acetate (2:1) as the eluent. ^1^H NMR (400 MHz, CD_3_OD): 0.91 (t, *J* = 7.3, 6H), 1.32 (m, 4H), 1.50 (m, 4H), 2.46 (t, *J* = 7.4, 4H), 3.67 (s, 2H), 7.58 (d, *J* = 8.1, 2H), 7.88 (d, *J* = 8.2, 2H), 9.98 (s, 1H). ^13^C NMR (100 MHz, CD_3_OD): 12.9, 20.2, 28.8, 53.5, 58.2, 129.3 (two close peaks), 135.6, 147.5, 192.5. MS(ESI): *m/z* 248.2 [M + H]^+^.

4-((Butyl(methyl)amino)methyl)benzaldehyde (**4e**).

Compound **4e** was obtained as a pale yellow oil (0.78 g, yield 76%) starting from alcohol derivative **7e** (1.02 g, 5.02 mmol) and was purified using a mixture of petroleum ether/ethyl acetate (8:2) as the eluent. ^1^H NMR (400 MHz, CD_3_OD): 0.94 (t, *J* = 7.4, 3H), 1.36 (m, 2H), 1.55 (m, 2H), 2.23 (s, 3H), 2.42 (t, *J* = 7.6, 2H), 3.61 (s, 2H), 7.57 (d, *J* = 8.0, 2H), 7.90 (d, *J* = 8.2, 2H), 10.00 (s, 1H). ^13^C NMR (100 MHz, CD_3_OD): 12.9, 20.2, 28.8, 41.1, 56.9, 60.1, 129.3, 129.7, 137.7, 151.2, 192.5. MS(ESI): *m/z* 206.1 [M + H]^+^.

4-((Diisopropylamino)methyl)benzaldehyde (**4f**) [56]

Compound **4f** was obtained as a pale yellow oil (0.03 g, yield 34%) starting from alcohol derivative **7f** (0.09 g, 0.41 mmol) and was purified using a mixture of petroleum ether/ethyl acetate (8:2) as the eluent. ^1^H NMR (400 MHz, CD_3_OD): 1.07 (d, *J* = 6.6, 12H), 3.06 (m, 2H), 3.78 (s, 2H), 7.62 (d, *J* = 8.1, 2H), 7.85 (d, *J* = 8.2, 2H), 9.96 (s, 1H). MS(ESI): *m/z* 220.1 [M + H]^+^.

4-((Benzyl(methyl)amino)methyl)benzaldehyde (**4g**) [57]

Compound **4g** was obtained as a pale yellow oil (0.13 g, yield 38%) starting from alcohol derivative **7g** (0.34 g, 1.41 mmol) and was purified using a mixture of petroleum ether/ethyl acetate (7:3) as the eluent. ^1^H NMR (400 MHz, CD_3_OD): 2.21 (s, 3H), 3.57 (s, 2H), 3.63 (s, 2H), 7.27 (t, *J* = 7.0, 1H), 7.31–7.42 (m, 4H), 7.60 (d, *J* = 8.0, 2H), 7.90 (d, *J* = 8.2, 2H), 9.99 (s, 1H). MS(ESI): *m/z* 240.1 [M + H]^+^.

4-((Benzyl(ethyl)amino)methyl)benzaldehyde (**4h**) [57]

Compound **4h** was obtained as a pale yellow oil (0.07 g, yield 55%) starting from alcohol derivative **7h** (0.17 g, 0.87 mmol) and was purified using a mixture of petroleum ether/ethyl acetate (8:2) as the eluent. ^1^H NMR (400 MHz, CD_3_OD): 1.11 (t, *J* = 7.1, 3H), 2.53 (q, *J* = 7.1, 2H), 3.60 (s, 2H), 3.67 (s, 2H), 7.24 (t, *J* = 7.2, 1H), 7.35 (t, *J* = 7.5, 2H), 7.38 (m, 2H), 7.61 (d, *J* = 8.1, 2H), 7.88 (d, *J* = 8.2, 2H), 9.97 (s, 1H). MS(ESI): *m/z* 254.1 [M + H]^+^.

### 6.2. Inhibition of Cholinesterases and Monoamine Oxidases

Human isoforms of ChEs (human recombinant AChE and BChE from human serum) and MAOs (from baculovirus-infected insect cells), purchased from Sigma Aldrich (Milan, Italy), were used for inhibition assays. Experiments were performed in 96-well plates (Greiner Bio-One, Kremsmünster, Austria) on the Infinite M1000 Pro plate reader (Tecan, Cernusco s.N., Italy), using already published protocols [46,58,59,60]. Inhibition data and constants (IC_50_s and K_i_s) were calculated with Prism (version 5.01 for Windows; GraphPad Software, San Diego, CA, USA).

The inhibition of human recombinant AChE or BChE from human serum was determined by applying Ellman’s spectrophotometric method as described in previously reported protocols [46,59]. Donepezil and tacrine were taken as positive controls in the AChE and BChE inhibition assays, respectively. The reported data agree with those previously reported [40]. The AChE activity was determined in an assay solution containing AChE (0.09 U/mL), 5,50-dithiobis(2-nitrobenzoic acid) (i.e., the Ellman’s reagent, 0.33 mM), the test compound (10 μM concentration, or seven scalar concentrations for compounds achieving >60% enzyme inhibition at 10 μM), in 0.1 M PBS pH 8.0. After 20 min incubation at 25 °C, the substrate acetylthiocholine iodide (5 μM) was added, and its hydrolysis rates were monitored for 5.0 min at 412 nm. The BChE inhibitory activity was similarly determined by using BChE (0.09 U/mL) and butyrylthiocholine iodide (5 μM) as the substrate. IC_50_ value, determined by the nonlinear regression method ‘log[inhibitor] vs. response’, or the % inhibition at 10 μM, is expressed as the mean ± SD of three independent measurements, each one performed in duplicate. The IC_50_ values, Michaelis–Menten curve fitting, and inhibition constant (Ki) were calculated by nonlinear regression using Prism software.

In MAOs’ inhibition assays, each test compound, at 10 μM concentration, was preincubated for 20 min at 37 °C with 50 μM kynuramine as the substrate in 0.1 M phosphate-buffered solution (PBS) pH 8.0 made 0.39 osmolar with KCl. After the addition of human recombinant MAO-A (250 U/mg) or MAO B (59 U/mg) and a further 30 min of incubation, NaOH was added, and the fluorescence read at 310/400 nm excitation/emission wavelength. For compounds achieving at least 60% inhibition of MAO at 10 μM concentration, seven scalar concentrations of each inhibitor were tested, and the concentration producing 50% inhibition of the MAO activity (IC50) was calculated by nonlinear regression. IC50 is expressed as mean ± SD of three independent measurements, each one performed in duplicate. Clorgiline and safinamide, retested in this work, were taken as positive controls in the MAO A/B assays. For the kinetic study on the inhibition mechanism of MAO B, three diverse scalar concentrations of the inhibitor and seven concentrations of kynuramine were used [40,61,62].

### 6.3. Molecular Docking Calculations

The three-dimensional (3D) structures of hAChE complexed with donepezil (PDB ID: 4EY7) and hMAO-B in complex with safinamide (PDB ID: 2V5Z) were taken from the Protein Data Bank. The protein preparation wizard available in the Schrödinger Suite (Schrödinger Release 2022-4) was employed to optimize X-ray crystal structures: missing side chains have been reconstructed, and protonation states at pH = 7.4 ± 0.0 have been predicted. Finally, energy minimization on the crystal structures was applied using the OPLS_2005 force field (Banks et al., 2005). Starting from SMILES annotations (Weininger, 1988), 3D structures of *E*-**1h** and *Z*-**1h** isomers have been generated, and their protonation states at pH = 7.4 ± 0.0 were computed by LigPrep tool of Schrödinger Suite using the OPLS_2005 force field. The same steps have been reiterated for *h*AchE and *h*MAO-B cognate ligands (i.e., donepezil and safinamide, respectively). Glide v9.1 (Schrödinger Release 2022-4: Glide, Schrödinger, LLC, New York, NY, USA, 2022) was adopted to perform docking simulations upon both targets applying OPLS_2005 force field. Docking calibration on cognate ligands was carried out in order to find out the most suitable docking protocol for each target: the Root Mean Square Deviation (RMSD) on heavy atoms between the docking pose and the X-ray coordinates of the cognate ligand was computed. In this respect, a cubic grid box of 15 Å for AchE (RMSD = 0.372) and a cubic grid box of 12 Å for MAO-B (RMSD = 0.467), both placed in the center of mass of the X-ray cognate ligands, were used to perform docking simulations. Standard Precision (SP) docking precision and OPLS_2005 force field were applied. The top-ranked docking poses of *E*-**1h** and *Z*-**1h** isomers were chosen to examine their target interactions and to compare them with the cognate ligands. All pictures have been made with Pymol (Schrodinger, LLC. 2010. The PyMOL Molecular Graphics System, Version 2.4.0).

## 7. Conclusions

Investigating the AChE/MAO-B dual inhibitory activity of some newly synthesized donepezil-like 2-benzylideneindan-1-one derivatives, which differ for bulkiness/lipophilicity of the unconjugated tertiary amino head, we gathered new clues about the photoswitchable *E/Z* isomerization controlling inhibitors’ binding to two AD-related target enzymes. Confirming our previous study [40], all the new compounds were obtained exclusively as *E* geometric isomers. Thanks to the photoswitching properties of the 2-benzylideneindan-1-one chromophore, *Z* isomers were generated in photostationary mixtures (75% of *Z* in all the examined cases) by exposure to UV-B radiation. The pure *E*-isomers showed submicromolar IC_50_ values on human AChE, in most cases, with a good selectivity over the BChE isoform. Interestingly, among the donepezil-like molecules synthesized so far, **1h** bearing the N-benzyl(ethyl)amino group at the *para* position of the benzylidene moiety proved to be a potent nanomolar AChE inhibitor, achieving an IC_50_ of 39 nM (i.e., threefold more potent of the parent *E*-**1a**). The irradiation and consequent enrichment in the *Z* isomer (75% *Z* in the PSS) led to a decrease in activity (IC_50_ of PSS-**1h** equals 53 nM), less sharp than that observed with the parent, **1a**. This suggests that the replacement of one ethyl group in *E*-**1a** with a more lipophilic benzyl moiety in *E*-**1h**, due to additional hydrophobic and aromatic interactions with residues into the enzyme binding pockets (e.g., Phe295 as shown by docking calculation), may improve the inhibitory potency of the *E*-**1h** compared to *E*-**1a**, at the same time flattening the differences in IC_50_s between the isomer *E* and the PSS *E/Z* mixture. No other compound showed an increase in inhibitory activity as appreciable as **1h**, whereas the N-Me(*^n^*Bu) analog **1e** showed the highest difference in AChE inhibition potency between the thermal steady state *E*-**1e** (IC_50_ = 0.102 μM) and the photoinduced one PSS-**1e** (IC_50_ = 1.36 μM).

The modifications carried out on the tertiary amino head of the parent compound **1a** seems well-tolerated by MAO-B; indeed, all compounds in the *E* geometry achieved submicromolar IC_50_ values and discrete selectivity over MAO-A. The effect of the *E*-to-*Z* photoinduced transition in MAO-B inhibition was more modest (no diastereoselectivity with **1h**) than that observed in AChE. Molecular docking studies performed with the potent dual inhibitor **1h** helped in highlighting the binding modes of the two diastereoisomers in AChE and MAO-B binding sites.

Considering the in vivo application limits of ultraviolet radiation (i.e., poor tissue penetrability and potential cytotoxicity), the data discussed herein represent the basis for the optimization of a donepezil-like molecule capable of synergistically inhibiting two target enzymes, namely AChE and MAO-B, involved in neurodegenerative diseases, with the added value of the photomodulation of the pharmacological effect, in order to obtain a tool of interest for the personalized medicine of the future. However, the achievement of the final goal deserves further efforts aimed at improving the characteristics of this family of compounds, paying attention to increasing the wavelength absorption and maintaining (or increasing) the inhibitory potency.

## Figures and Tables

**Table 1:** Inhibition data of recombinant human cholinesterases (AChE and BChE) and monoamine oxidases (MAOs A and B) by *E* isomers and *E/Z* mixtures of compounds **1a**–**h**.

Cmpds ^a^	IC_50_ (μM) or % Inhibition at 10 μM ^b^			
	AChE ^c^	BChE ^c^	MAO-A ^c^	MAO-B ^c^
*E*-**1a**	0.105 ± 0.005 ^f^	10.0 ± 0.15 ^f^	(33 ± 3) ^f^	0.260 ± 0.012 ^f^
*E/Z*-**1a**	0.780 ± 0.013 ^f^	(28 ± 6%) ^f^	(45 ± 5) ^f^	1.59 ± 0.04 ^f^
*E*-**1b**	0.155 ± 0.010	n.a.	(33 ± 1)	0.686 ± 0.059
*E/Z*-**1b**	0.915 ± 0.138	(25 ± 10%)	(32 ± 2)	0.839 ± 0.123
*E*-**1c**	0.174 ± 0.017	(28 ± 7%)	(15 ± 1)	0.479 ± 0.081
*E/Z*-**1c**	0.295 ± 0.045	(20 ± 7%)	2.98 ± 0.08	0.743 ± 0.048
*E*-**1d**	0.990 ± 0.050	(28 ± 9%)	(12 ± 4)	0.338 ± 0.036
*E/Z*-**1d**	2.28 ± 0.21	n.a.	(37 ± 2)	0.382 ± 0.006
*E*-**1e**	0.102 ± 0.019	5.00 ± 0.45	(20 ± 5)	0.178 ± 0.086
*E/Z*-**1e**	1.36 ± 0.06	n.a.	(25 ± 5)	0.651 ± 0.061
*E*-**1f**	0.261 ± 0.014	10.0 ± 0.85	(22 ± 2)	0.178 ± 0.086
*E/Z*-**1f**	1.41 ± 0.09	(20 ± 3%)	3.19 ± 0.10	0.241 ± 0.026
*E*-**1g**	0.115 ± 0.004	9.90 ± 0.11	(39 ± 7)	0.292 ± 0.060
*E/Z*-**1g**	0.135 ± 0.062	(40 ± 2%)	(49 ± 1)	0.354 ± 0.070
*E*-**1h**	0.039 ± 0.001	7.00 ± 0.01	(13 ± 6)	0.355 ± 0.134
*E/Z*-**1h**	0.053 ± 0.006	10.0 ± 0.32	(39 ± 5)	0.358 ± 0.008
Donepezil ^d^	0.021 ± 0.005	4.80 ± 1.00		
Tacrine ^d^	0.090 ± 0.005	0.025 ± 0.003		
Clorgiline ^e^			0.0022 ± 0.0002	2.48 ± 0.43
Safinamide ^e^			(18 ± 3)	0.028 ± 0.001

^a^ The diastereomeric *E/Z* mixtures’ ratios (25:75) at the photostationary state after UV-B light irradiation of the *E* methanolic solutions were established for all compounds by ^1^H NMR spectrometry. ^b^ IC_50_ values determined by interpolation of the sigmoidal dose–response curves as obtained by regression with GraphPad Prism software (ver.5.01) of at least seven data points, or percent inhibition in parentheses for samples achieving less than 50% inhibition at 10 μM concentration; n.a. = not active; data are means ± SD of three independent measurements. ^c^ Human cholinesterases and monoamine oxidase isoforms. ^d^ donepezil and tacrine were used as positive controls in AChE and BChE inhibition assays, respectively. ^e^ Clorgiline and safinamide were used as positive controls in MAO-A and MAO-B inhibition assays, respectively. ^f^ Data re-determined in this work, agreeing with those previously reported.

## Data Availability

All data presented in this study are available in the article and in Supplementary Information.

## Supplementary Materials

The following supporting information can be downloaded at: https://www.mdpi.com/article/10.3390/molecules28155857/s1, ^1^H NMR spectra of all the compounds and ^13^C NMR spectra of the newly synthesized compounds. Additional UV-vis spectra.

## Sample Availability

Samples of the compounds presented are available from the authors.
