# Supplementary material for: Investigation on Novel E/Z 2-Benzylideneindan-1-One-Based Photoswitches with AChE and MAO-B Dual Inhibitory Activity
Source: Molecules. 2023 Aug 3;28(15):5857. doi: 10.3390/molecules28155857 (PMC10421270; doi:10.3390/molecules28155857)
Supplement: Supplementary file 1 [file molecules-28-05857-s001.zip › molecules-2496999-supplementary.pdf]

Supplementary data for

Investigation on Novel E/Z 2-Benzylideneindan-1-one-based Photoswitches with AChE and MAO B Dual Inhibitory Activities

Marco Paolino,[a,†] Modesto de Candia,[b,†] Rosa Purgatorio,[b] Marco Catto,[b] Mario Saletti,[a] Anna Rita Tondo,[b] Orazio Nicolotti,[b] Andrea Cappelli,[a] Antonella Brizzi,[a] Claudia Mugnaini,[a] Federico Corelli,[a] and Cosimo D. Altomare[b,*]

[a]Department of Biotechnology, Chemistry and Pharmacy, University of Siena, Via A. Moro 2, I-53100 Siena, Italy;

[b]Department of Pharmacy-Pharmaceutical Sciences, University of Bari Aldo Moro, Via E. Orabona 4, I-70125 Bari, Italy.

* Correspondence: cosimodamiano.altomare@uniba.it

† These authors contributed equally to this work.

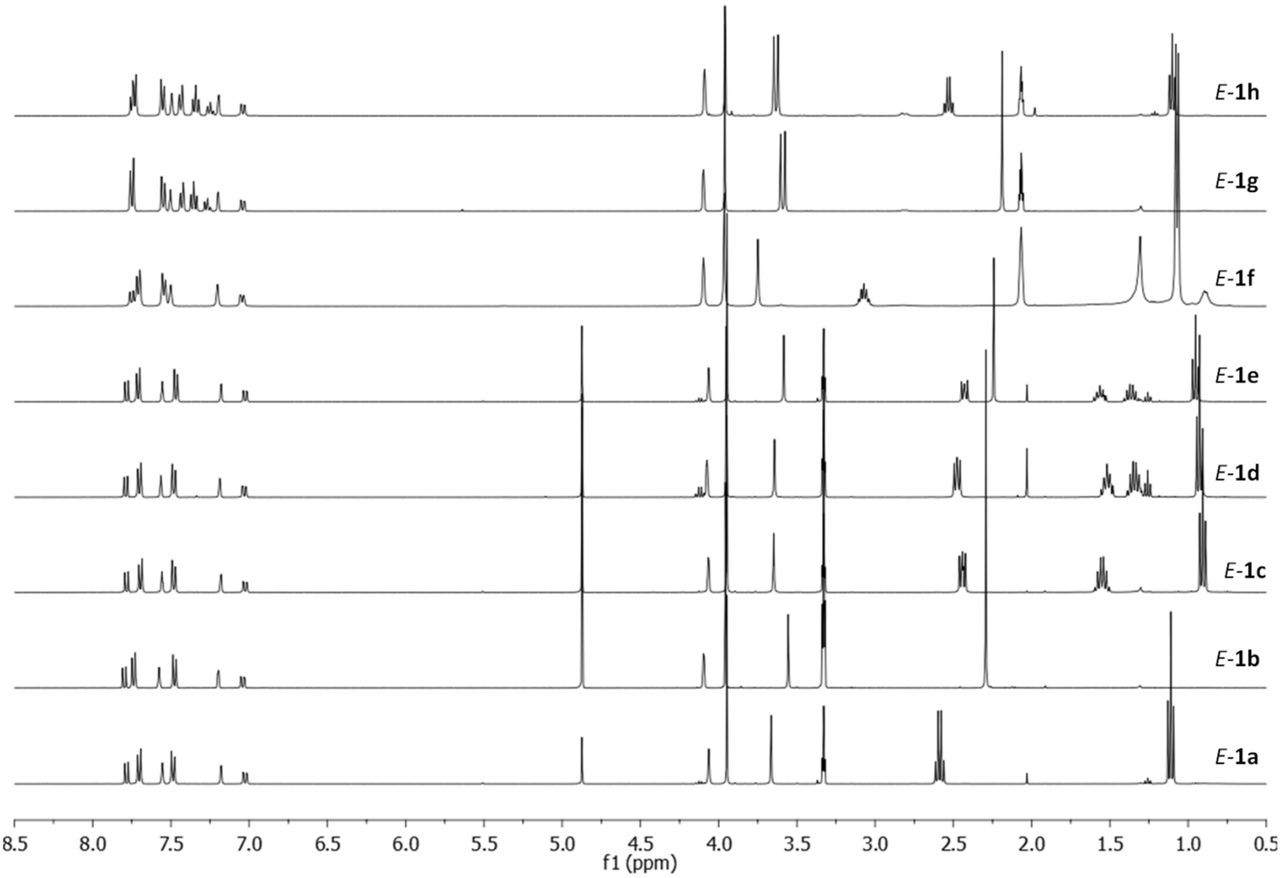

Figure S1. Comparison of ^{1}H NMR spectra (400 MHz) of compounds *E*-**1a-e** in CD_3OD and compounds *E*-**1f-h** in $(CD_3)_2CO$.

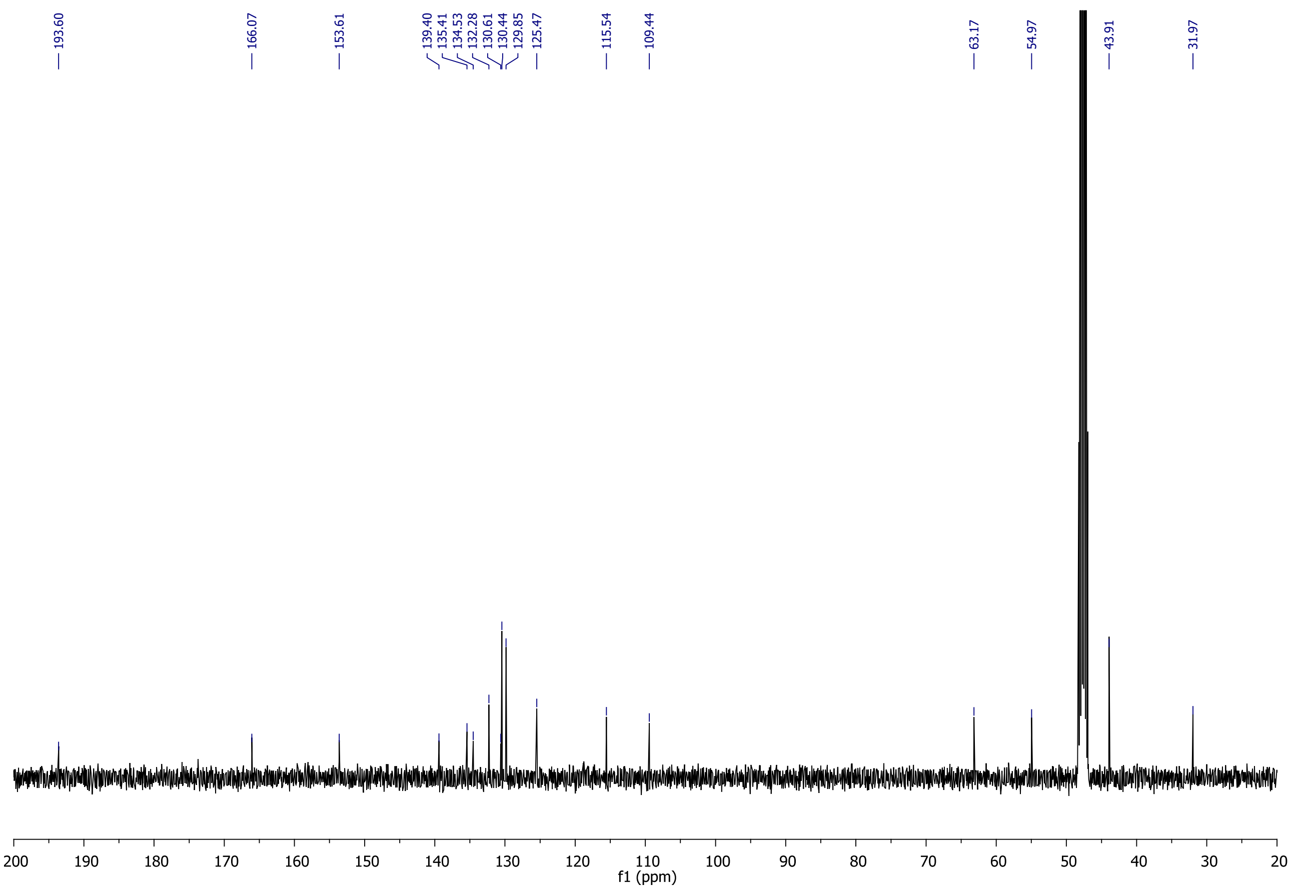

Figure S2. ^{13}C NMR spectrum (100 MHz, CD_3OD) of compound **1b**.

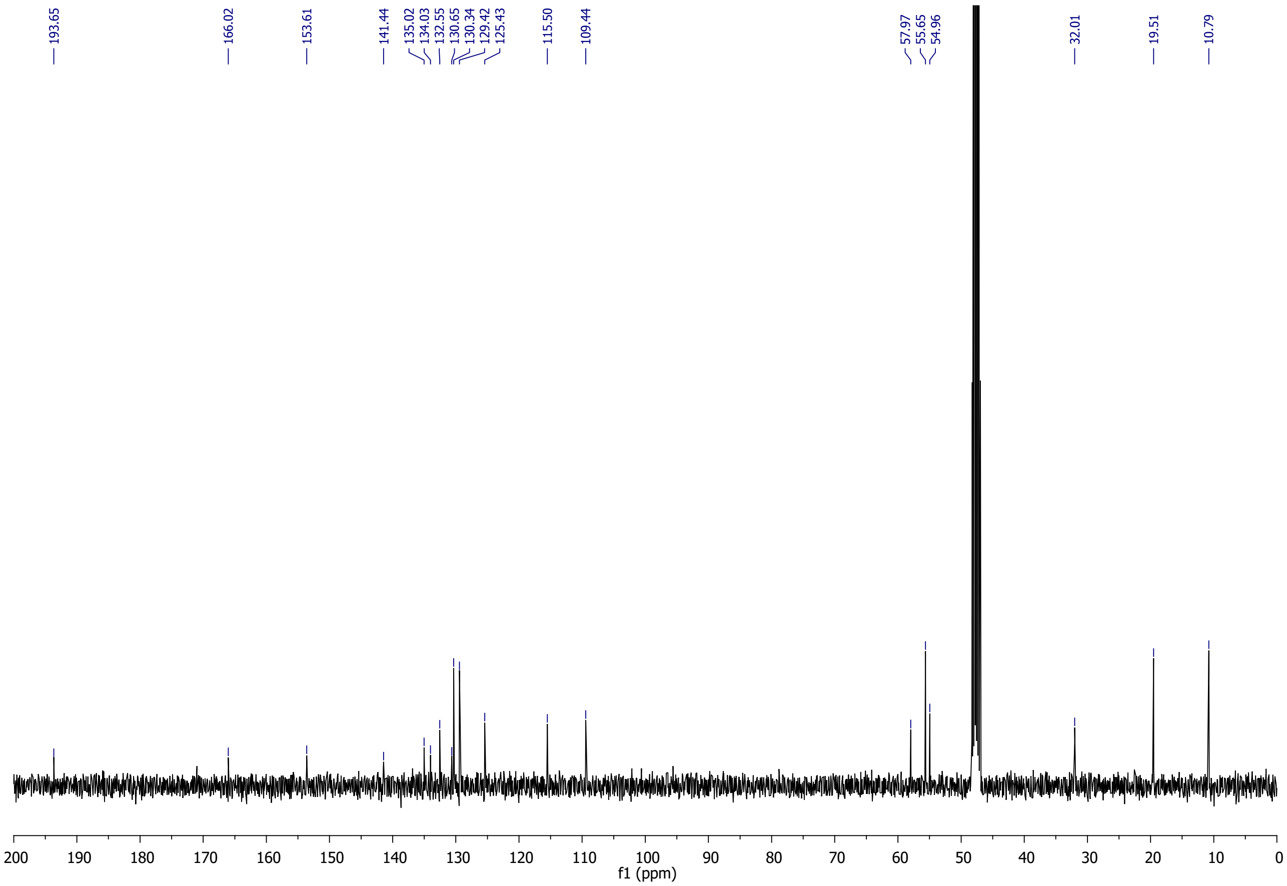

Figure S3. ^{13}C NMR spectrum (100 MHz, CD_3OD) of compound **1c**.

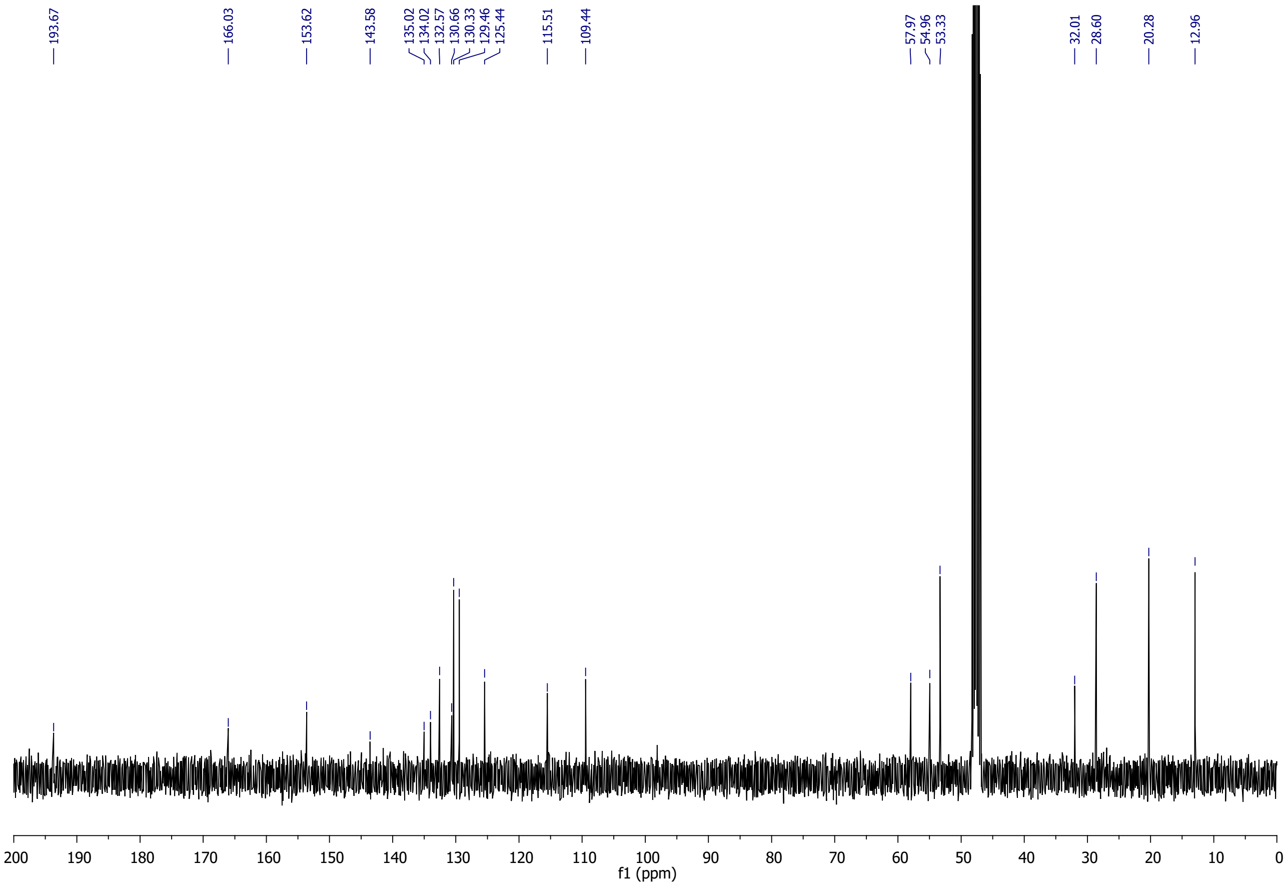

Figure S4. ^{13}C NMR spectrum (100 MHz, CD_3OD) of compound **1d**.

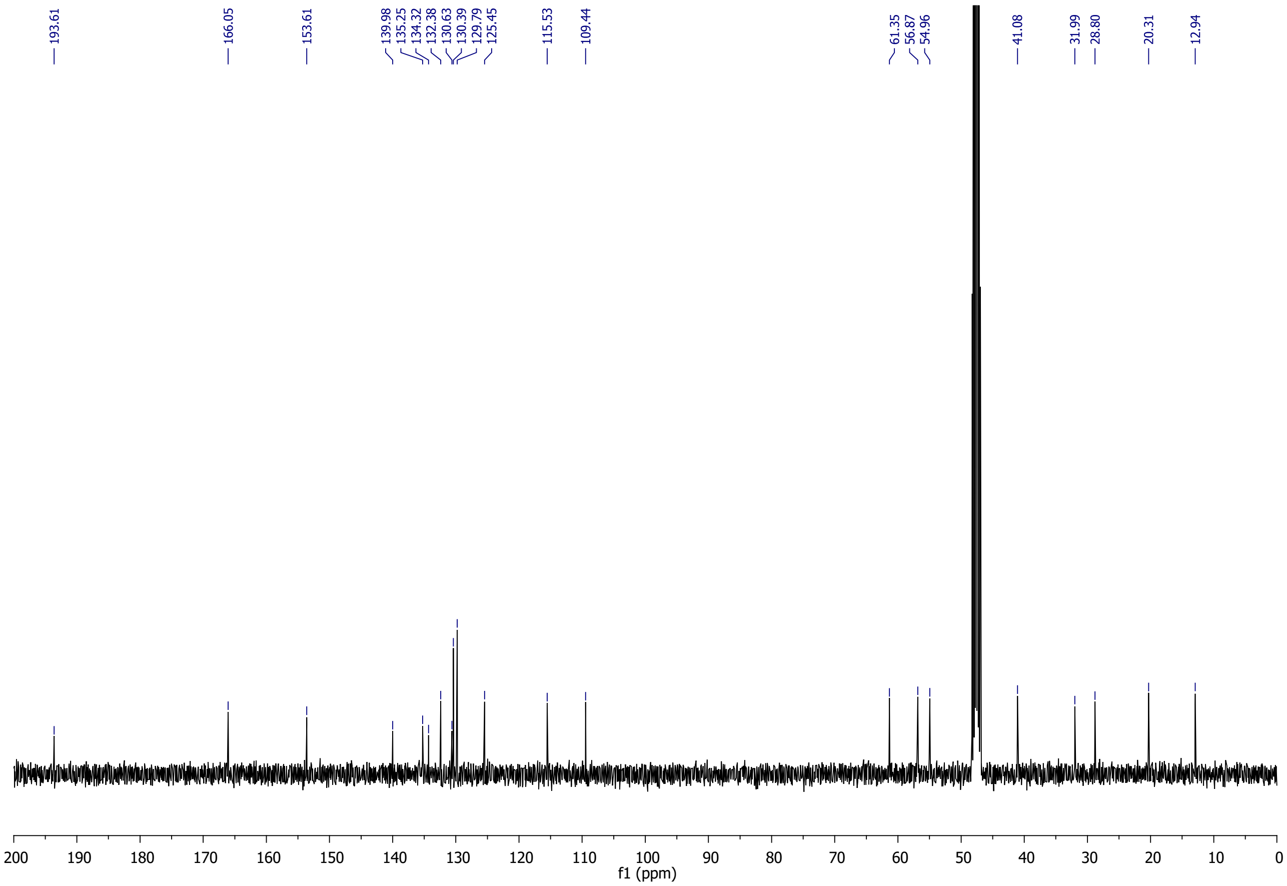

Figure S5. ^{13}C NMR spectrum (100 MHz, CD_3OD) of compound **1e**.

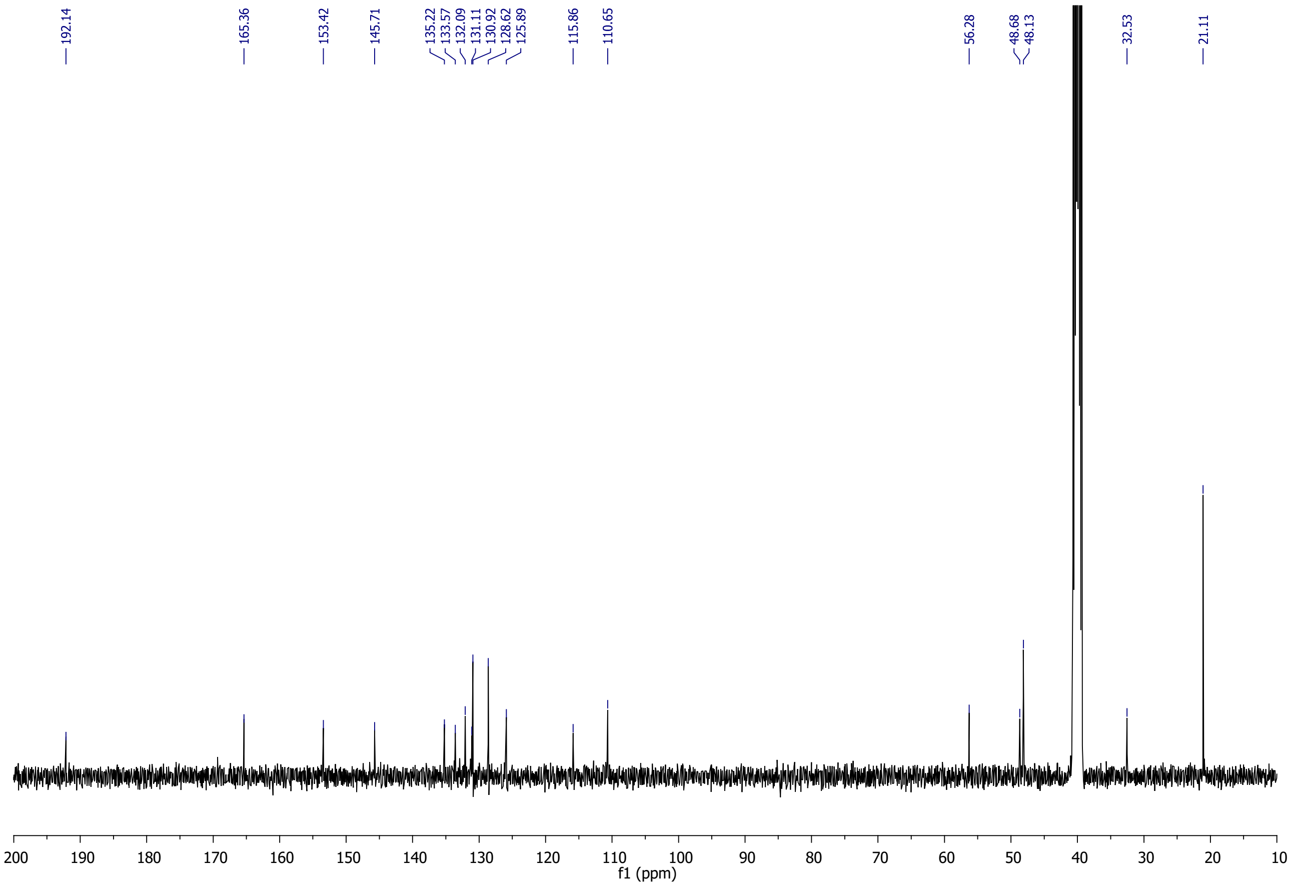

Figure S6. ^{13}C NMR spectrum (100 MHz, $(CD_3)_2SO$) of compound **1f**.

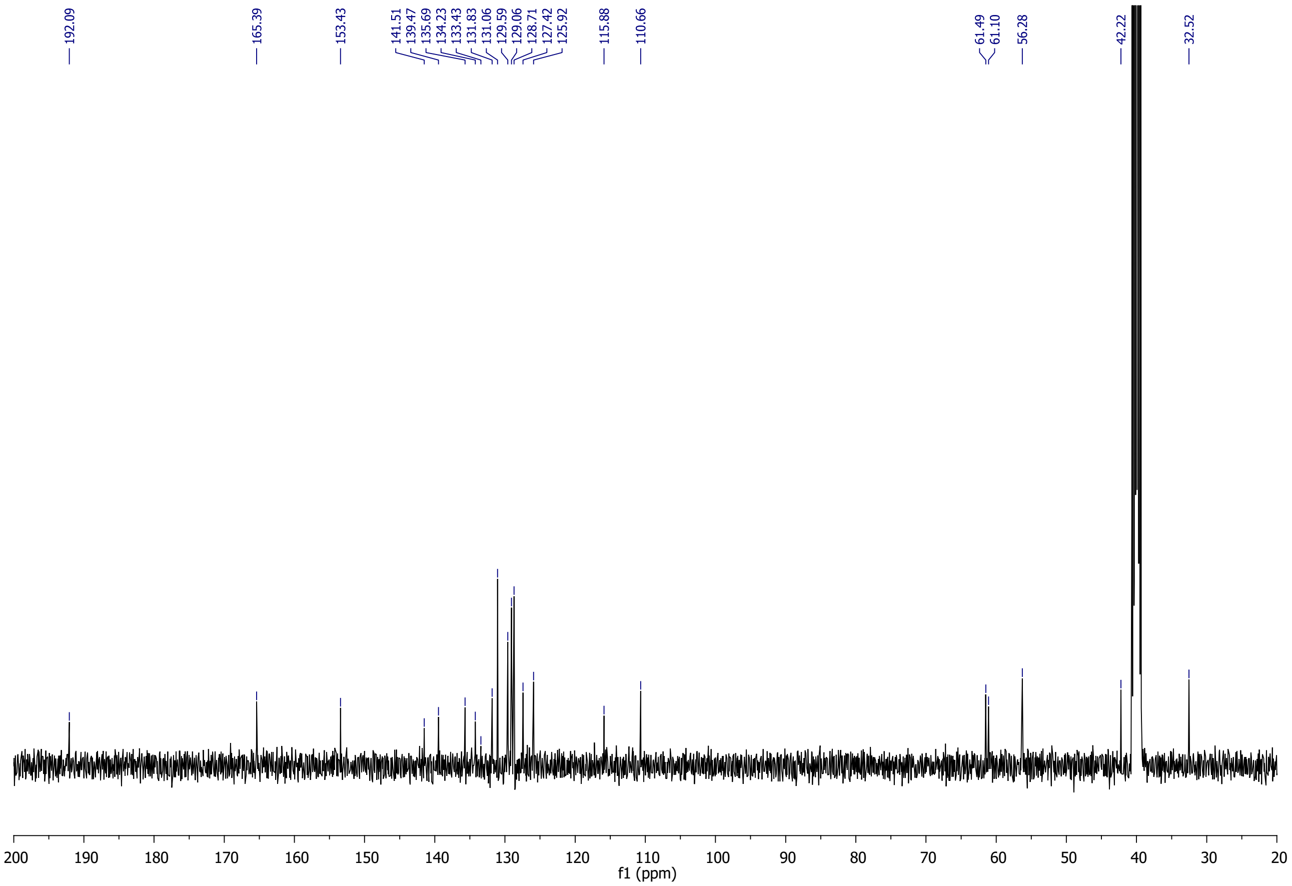

Figure S7. ^{13}C NMR spectrum (100 MHz, $(CD_3)_2SO$) of compound **1g**.

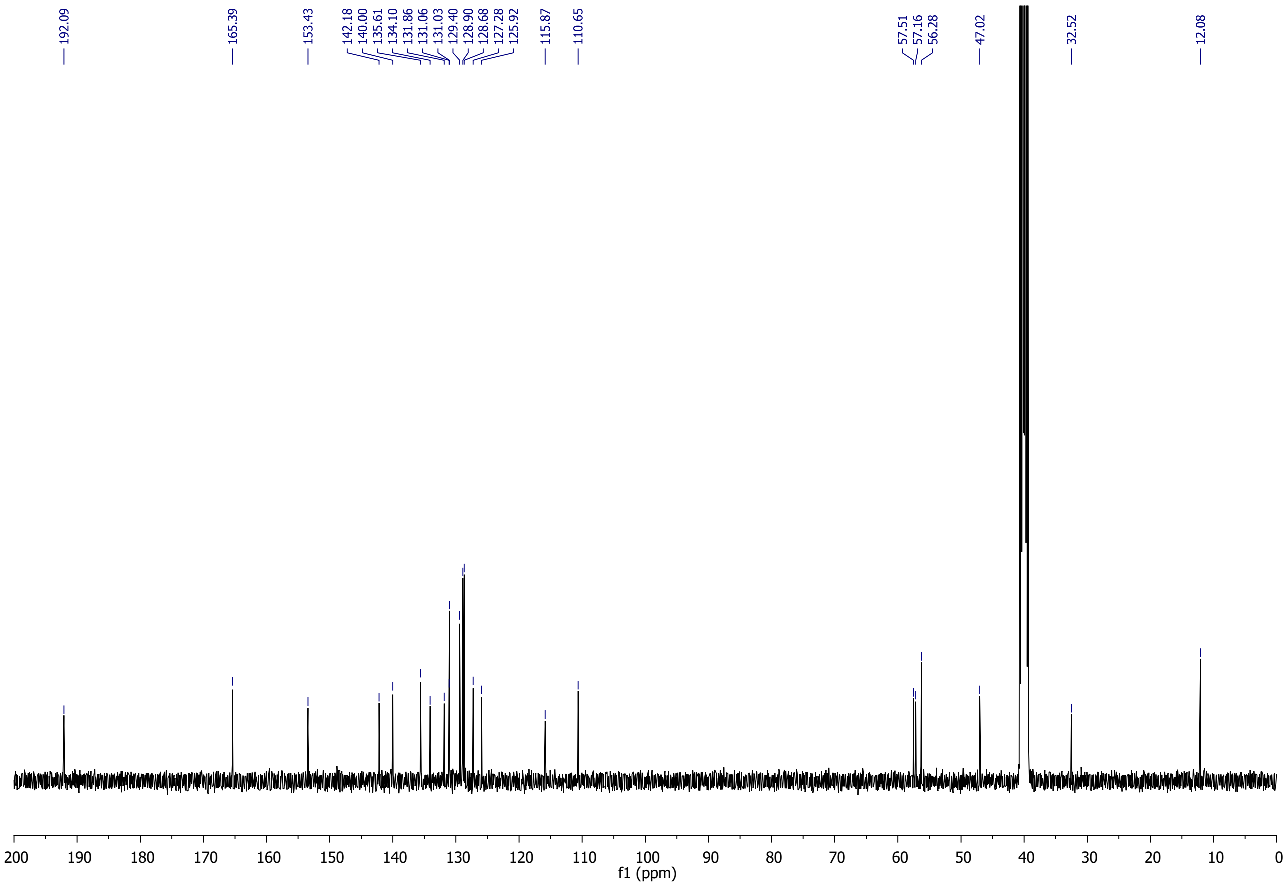

Figure S8. ^{13}C NMR spectrum (100 MHz, $(CD_3)_2SO$) of compound **1h**.

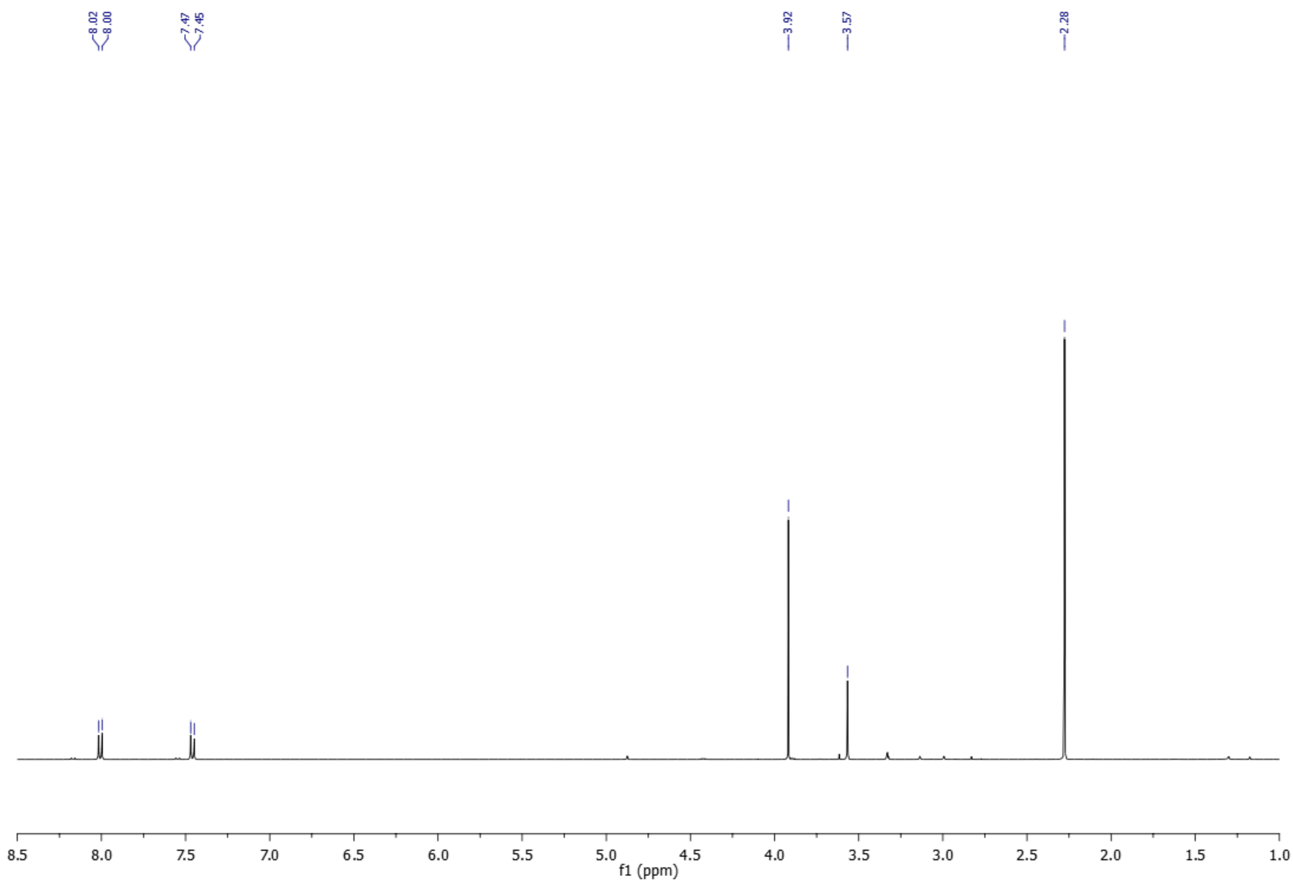

Figure S9. ^{1}H NMR spectrum (400 MHz, CD_3OD) of compound **6b**.

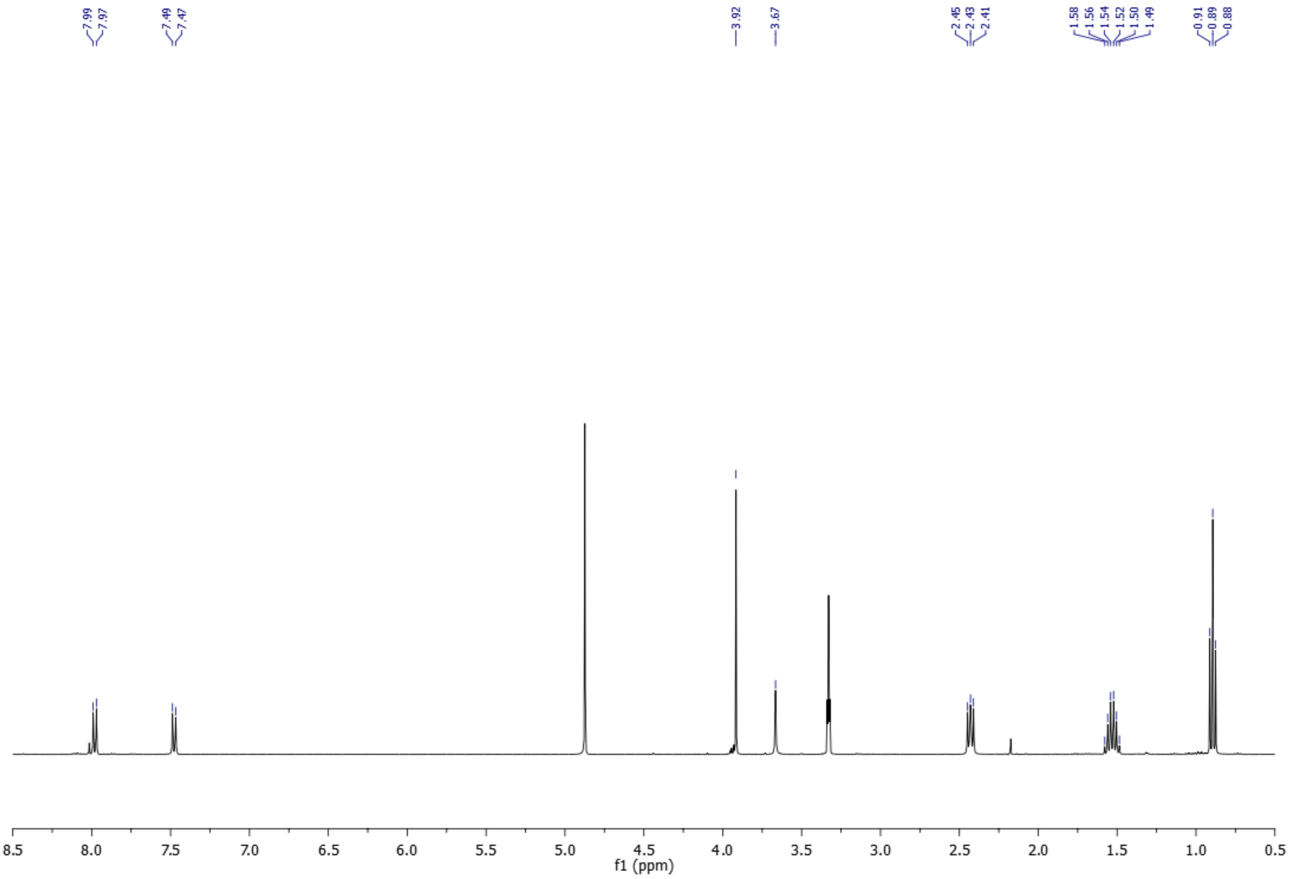

Figure S10. ^{1}H NMR spectrum (400 MHz, CD_3OD) of compound **6c**.

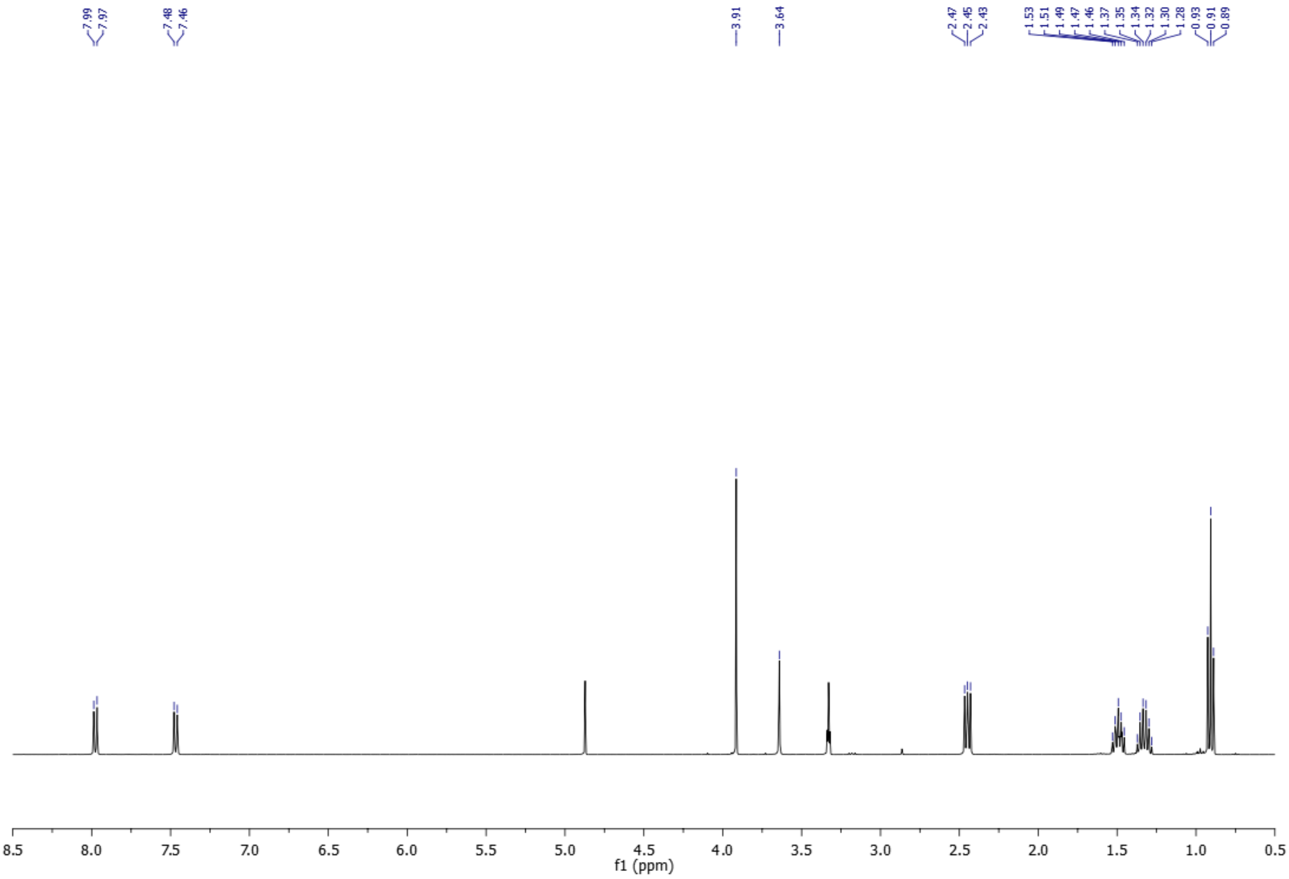

Figure S11. ^{1}H NMR spectrum (400 MHz, CD_3OD) of compound **6d**.

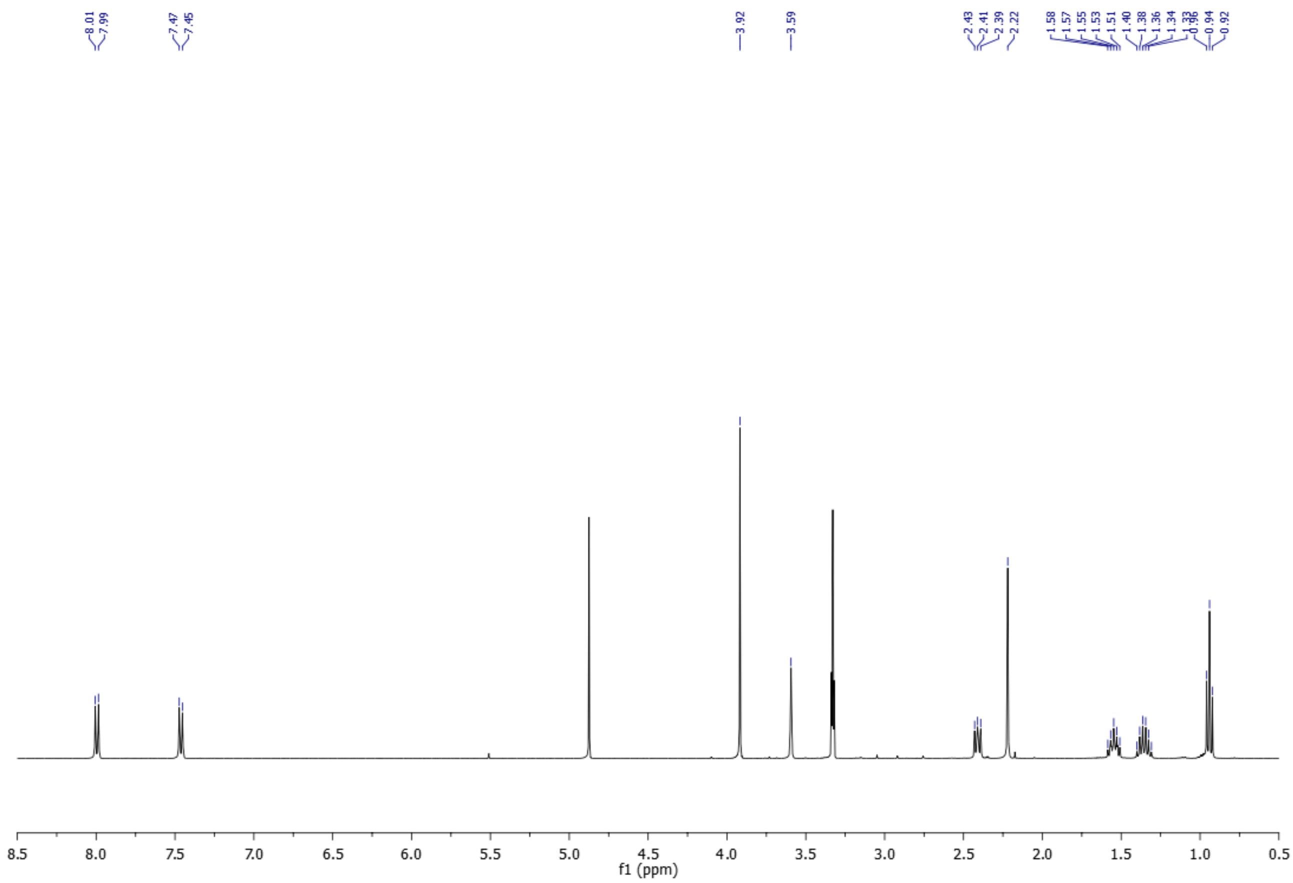

Figure S12. ^{1}H NMR spectrum (400 MHz, CD_3OD) of compound **6e**.

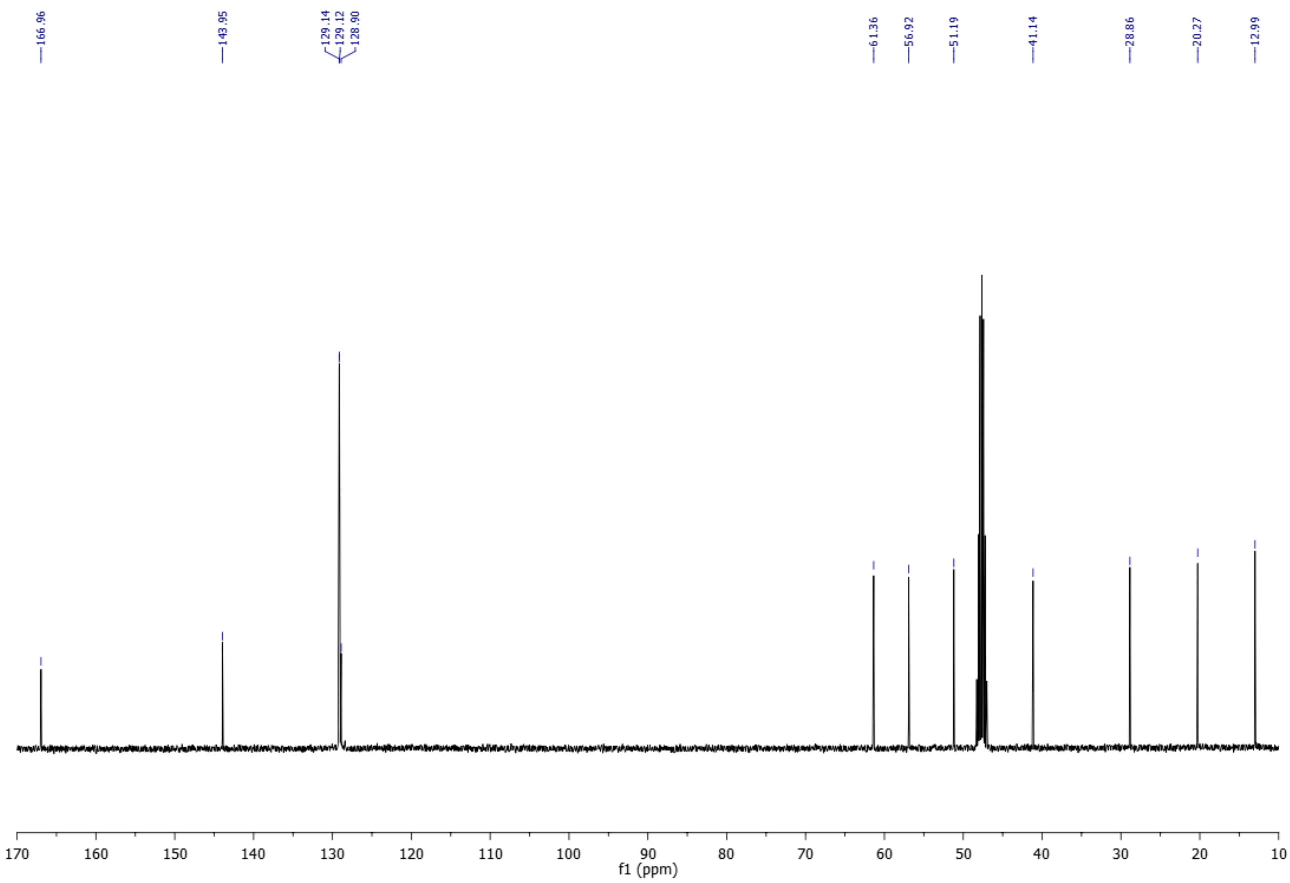

Figure S13. ^{13}C NMR spectrum (100 MHz, CD_3OD) of compound **6e**.

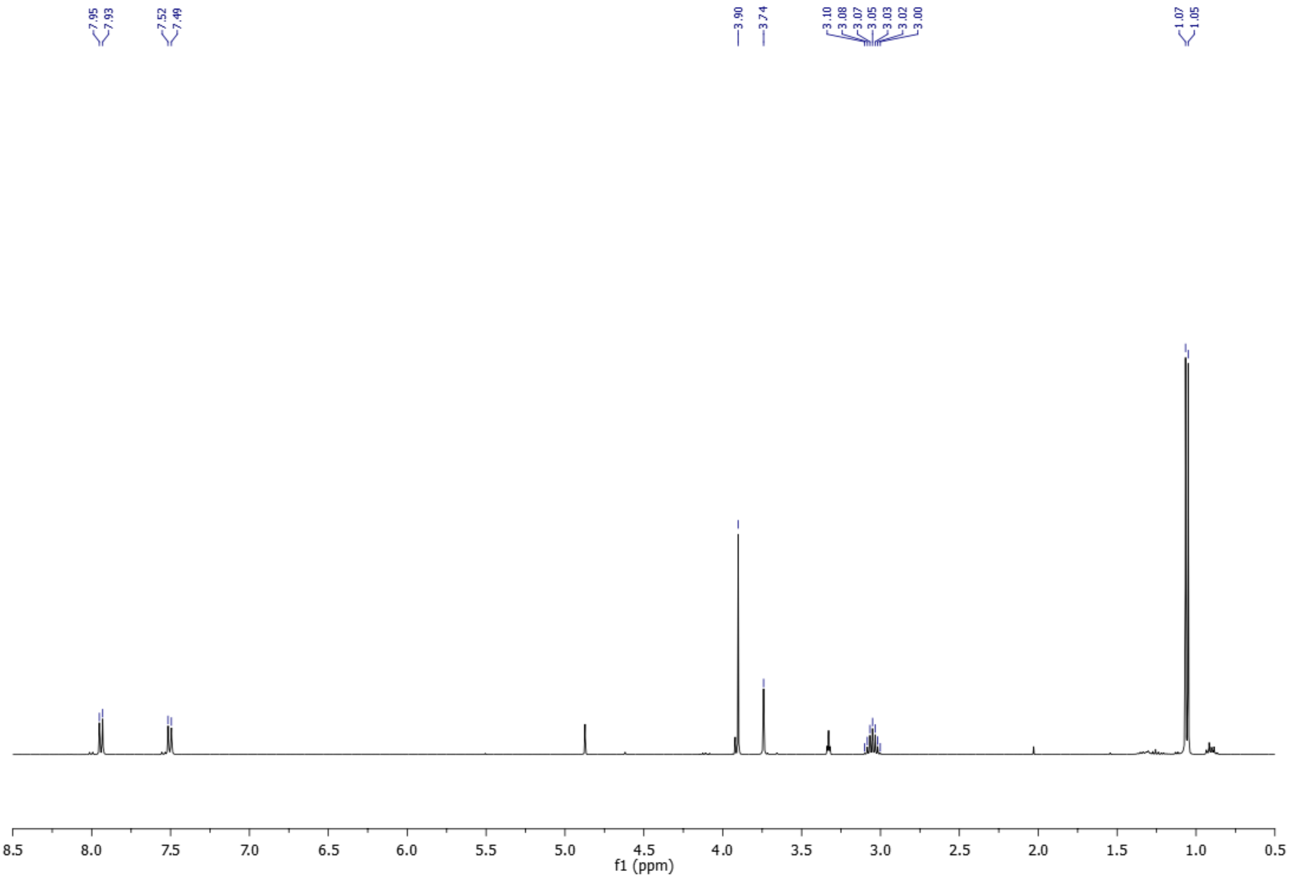

Figure S14. ^{1}H NMR spectrum (400 MHz, CD_3OD) of compound **6f**.

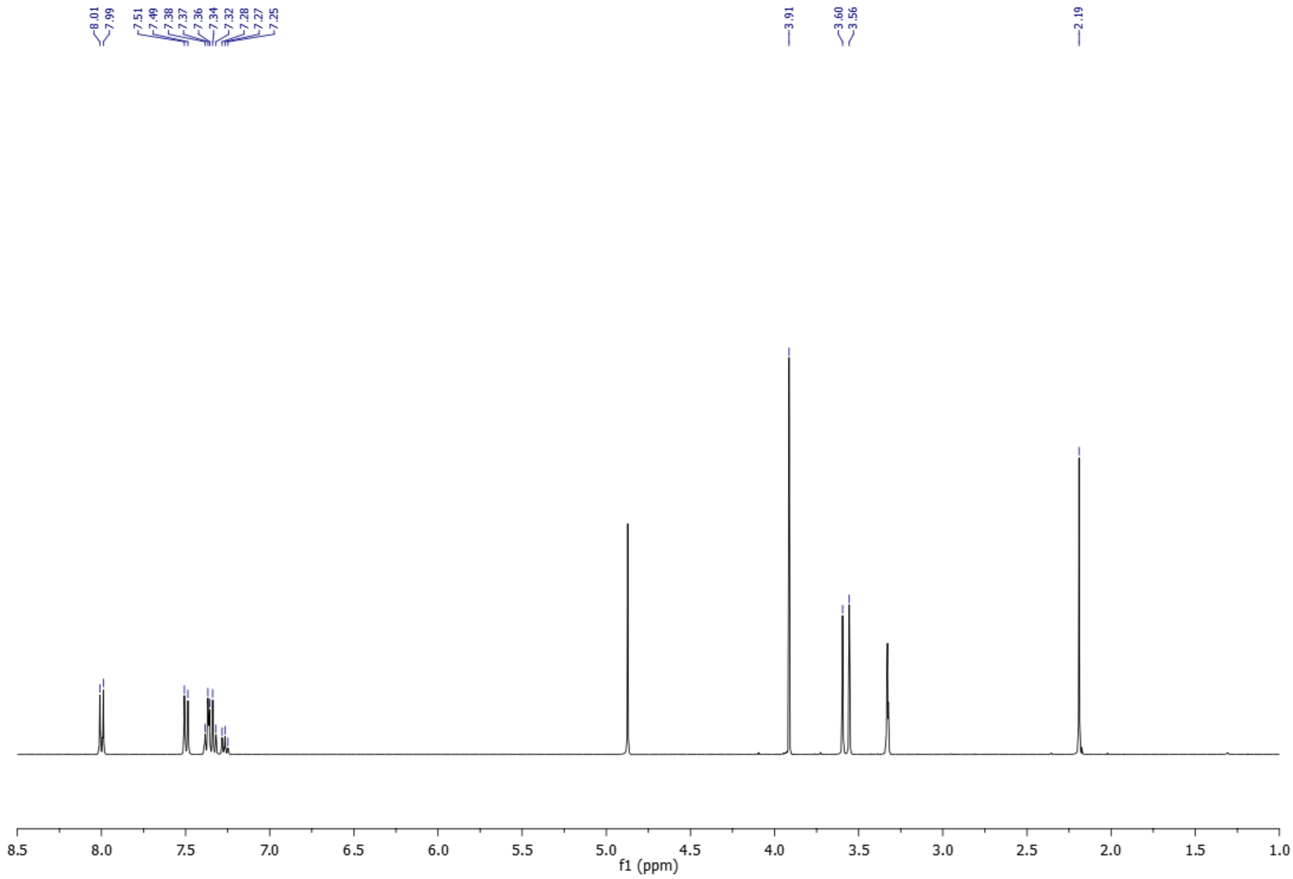

Figure S15. ^{1}H NMR spectrum (400 MHz, CD_3OD) of compound **6g**.

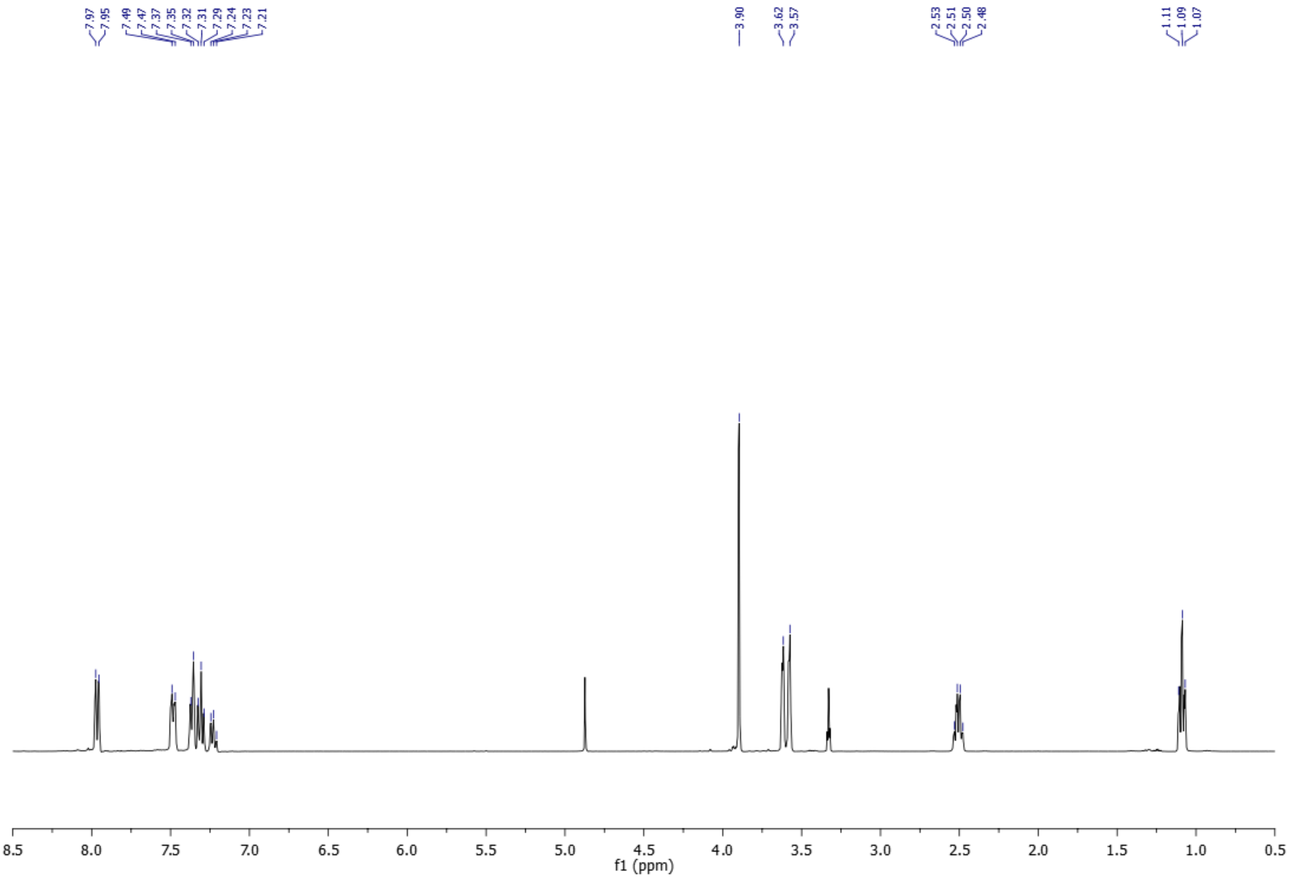

Figure S16. ^{1}H NMR spectrum (400 MHz, CD_3OD) of compound **6h**.

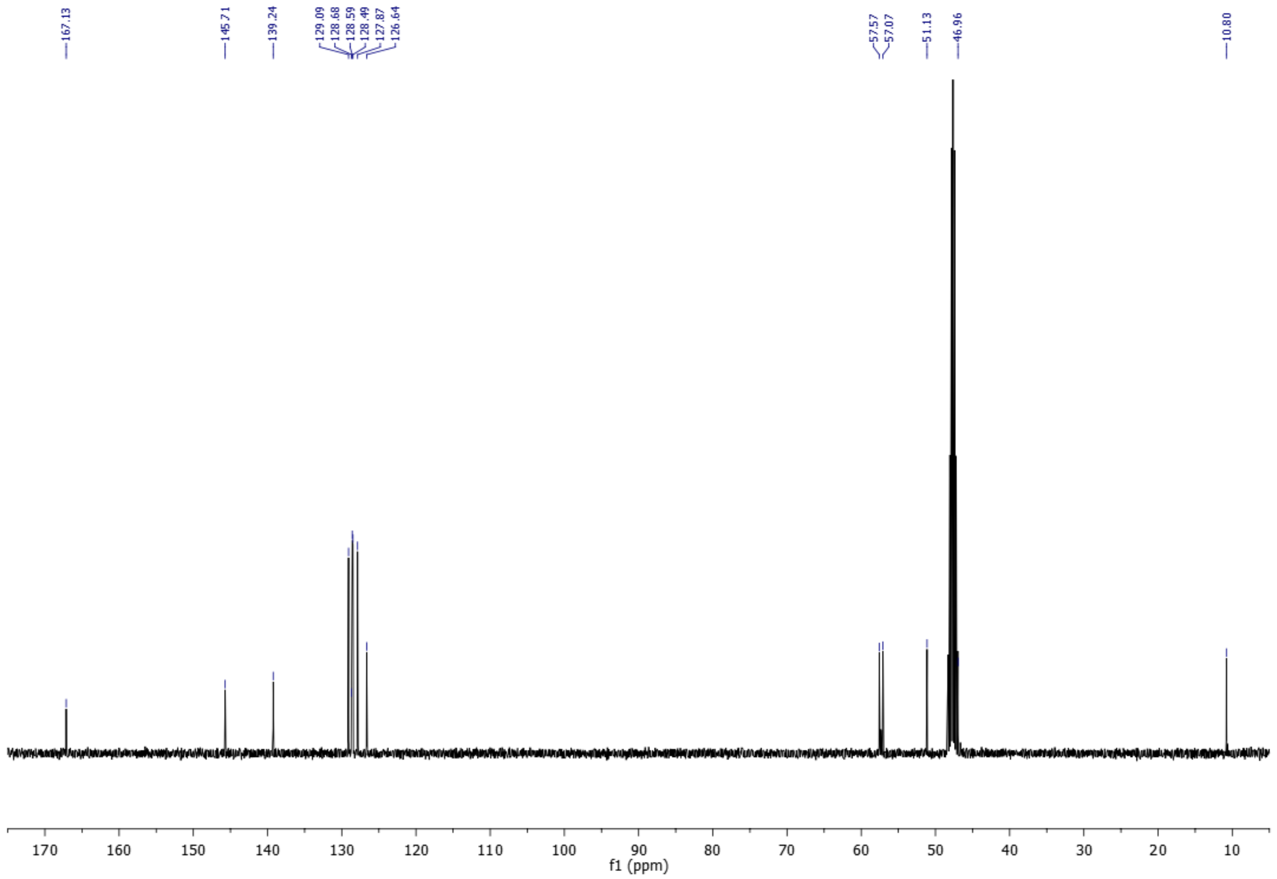

Figure S17. ^{13}C NMR spectrum (100 MHz, CD_3OD) of compound **6h**.

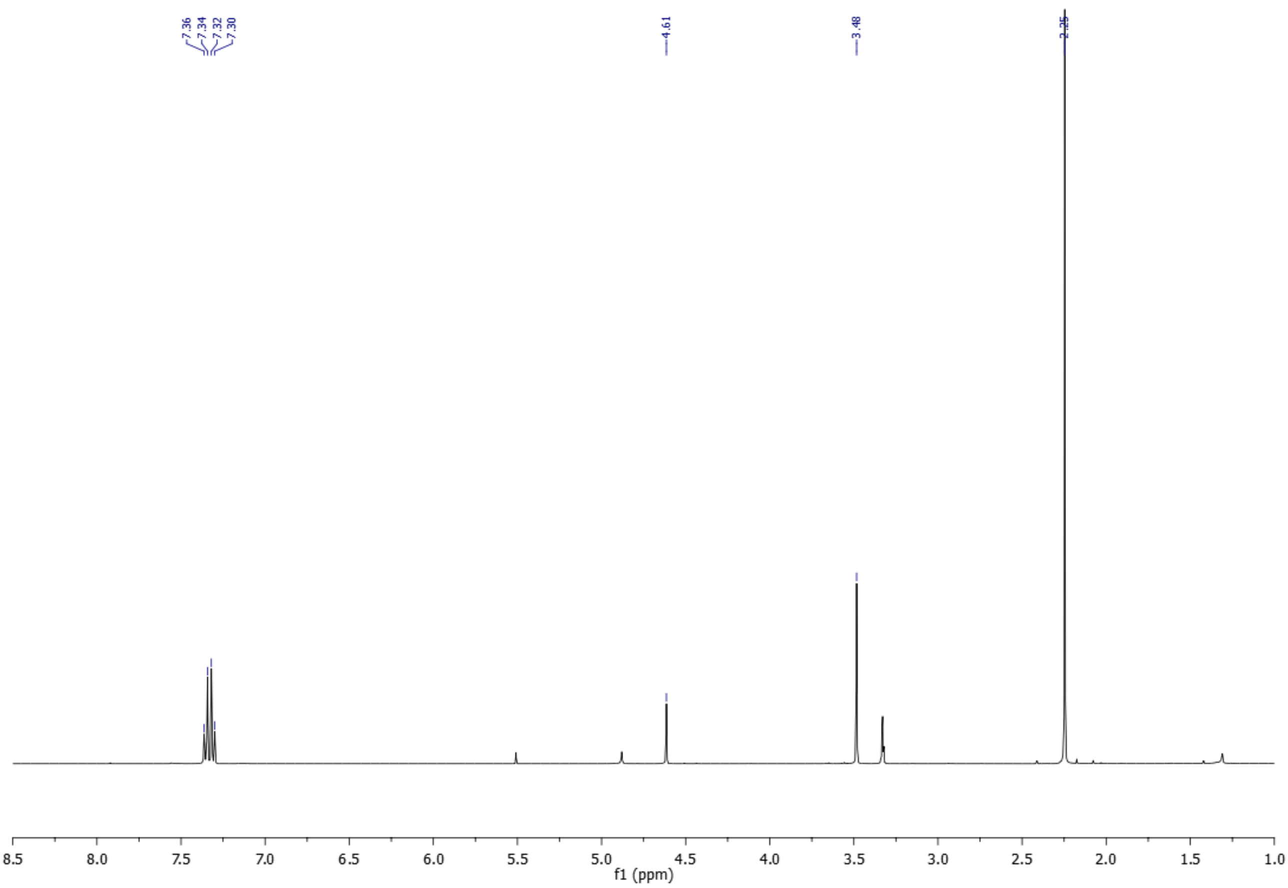

Figure S18. ^{1}H NMR spectrum (400 MHz, CD_3OD) of compound **7b**.

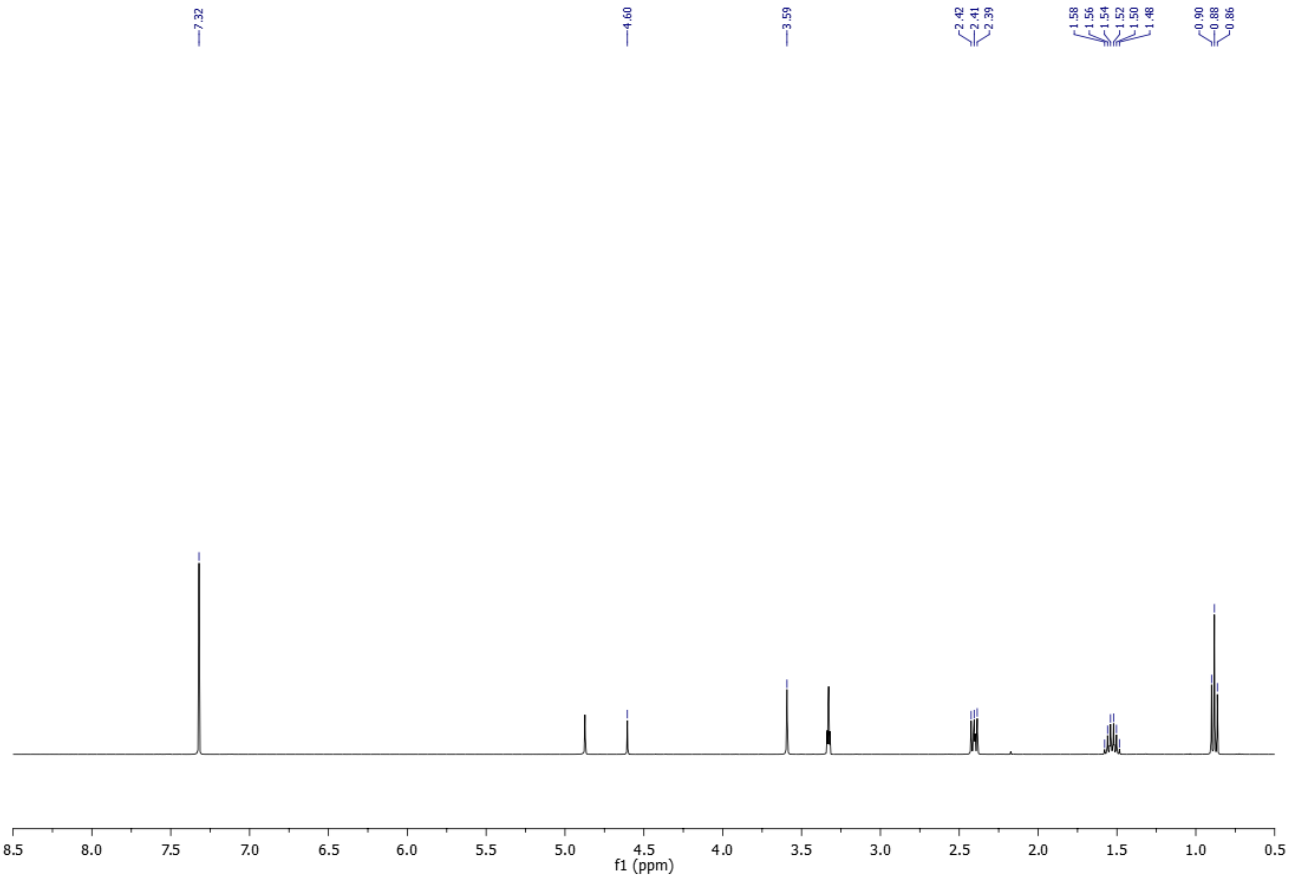

Figure S19. ^{1}H NMR spectrum (400 MHz, CD_3OD) of compound **7c**.

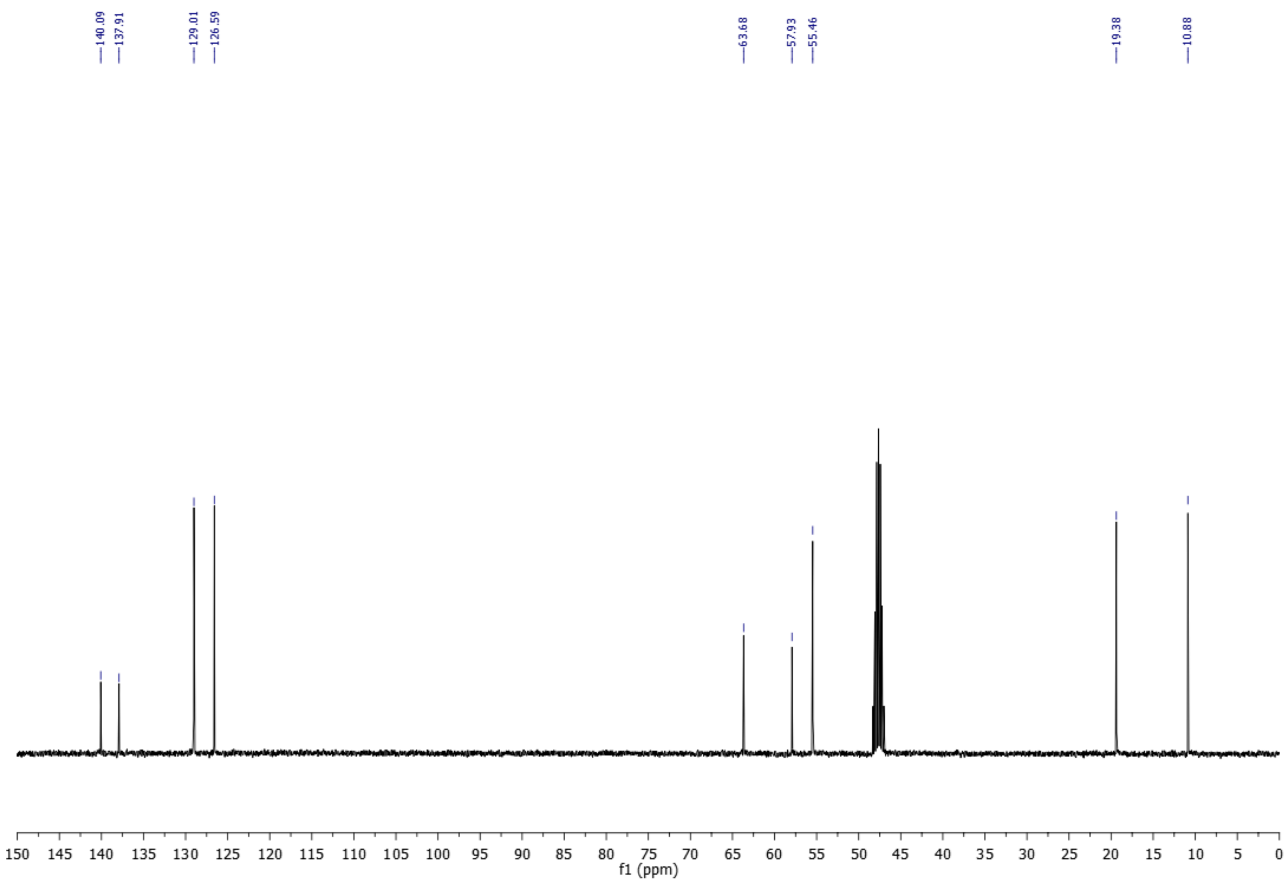

Figure S20. ^{13}C NMR spectrum (100 MHz, CD_3OD) of compound **7c**.

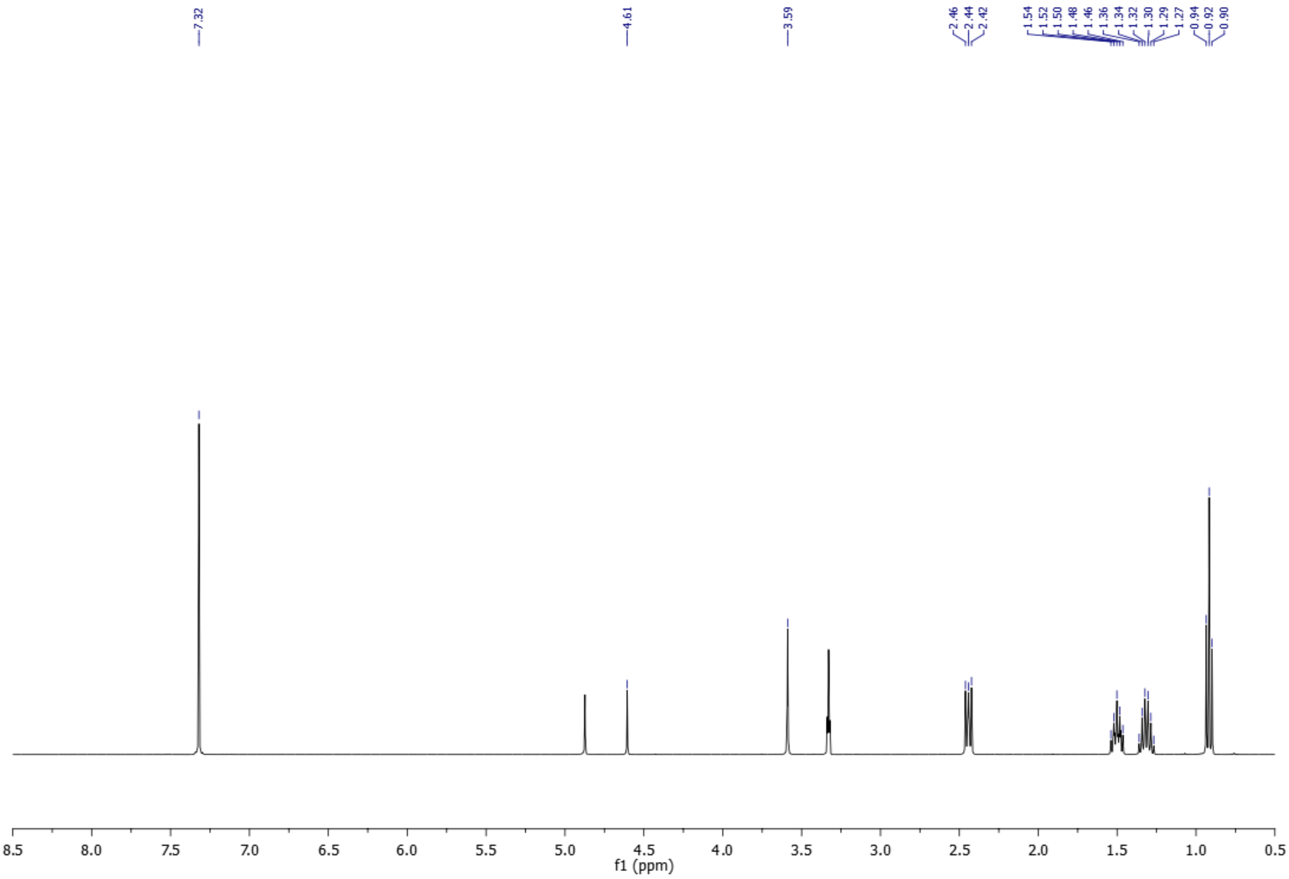

Figure S21. ^{1}H NMR spectrum (400 MHz, CD_3OD) of compound **7d**.

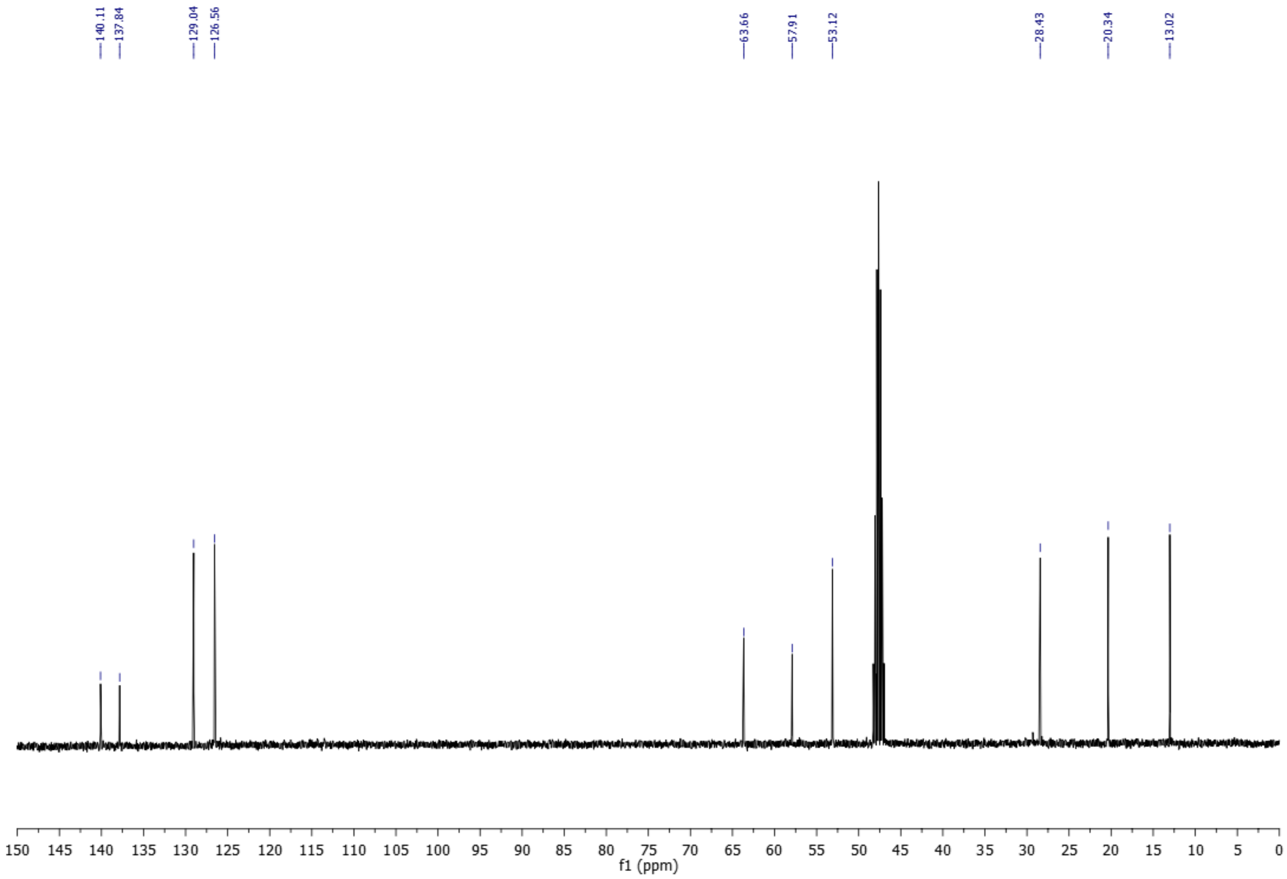

Figure S22. ^{13}C NMR spectrum (100 MHz, CD_3OD) of compound **7d**.

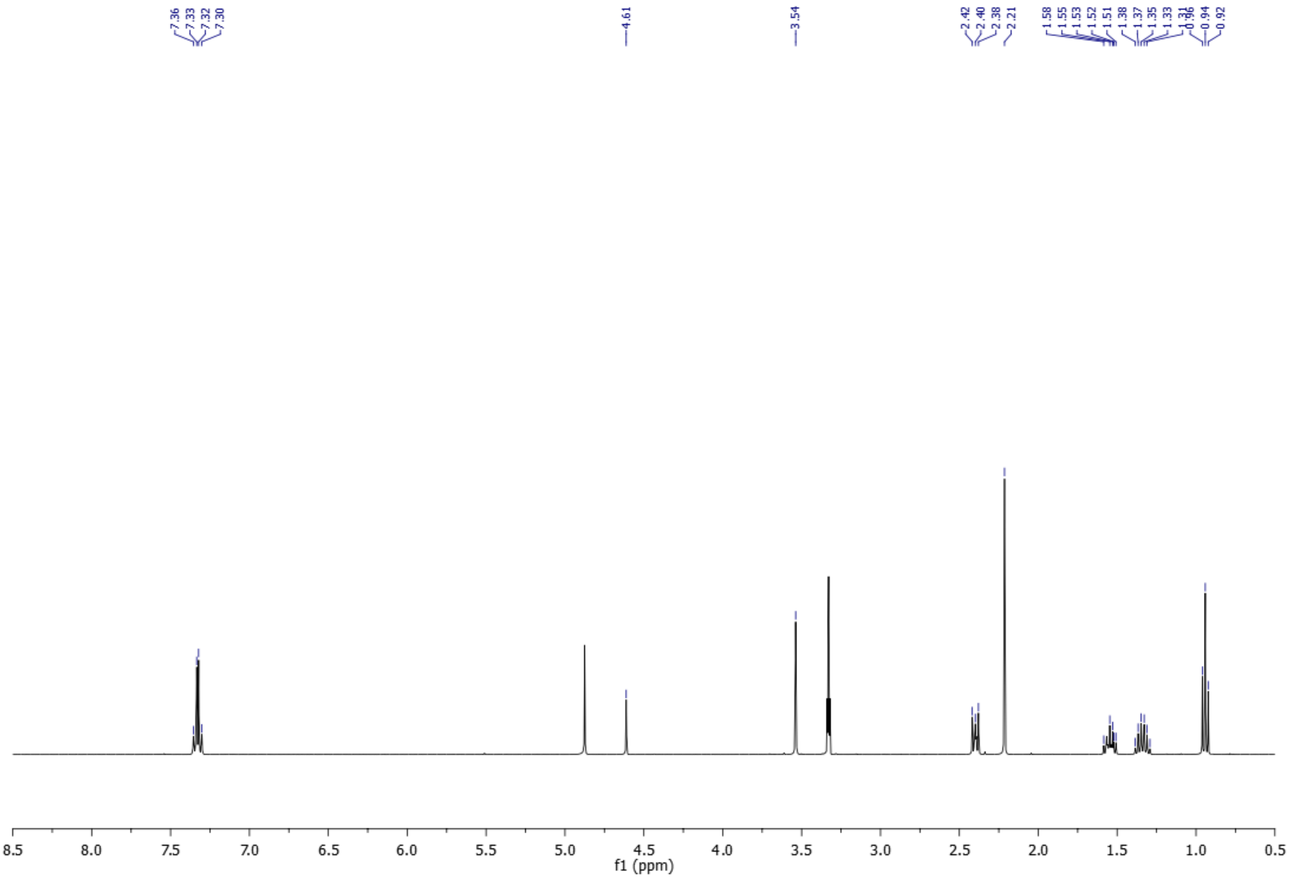

Figure S23. ^{1}H NMR spectrum (400 MHz, CD_3OD) of compound **7e**.

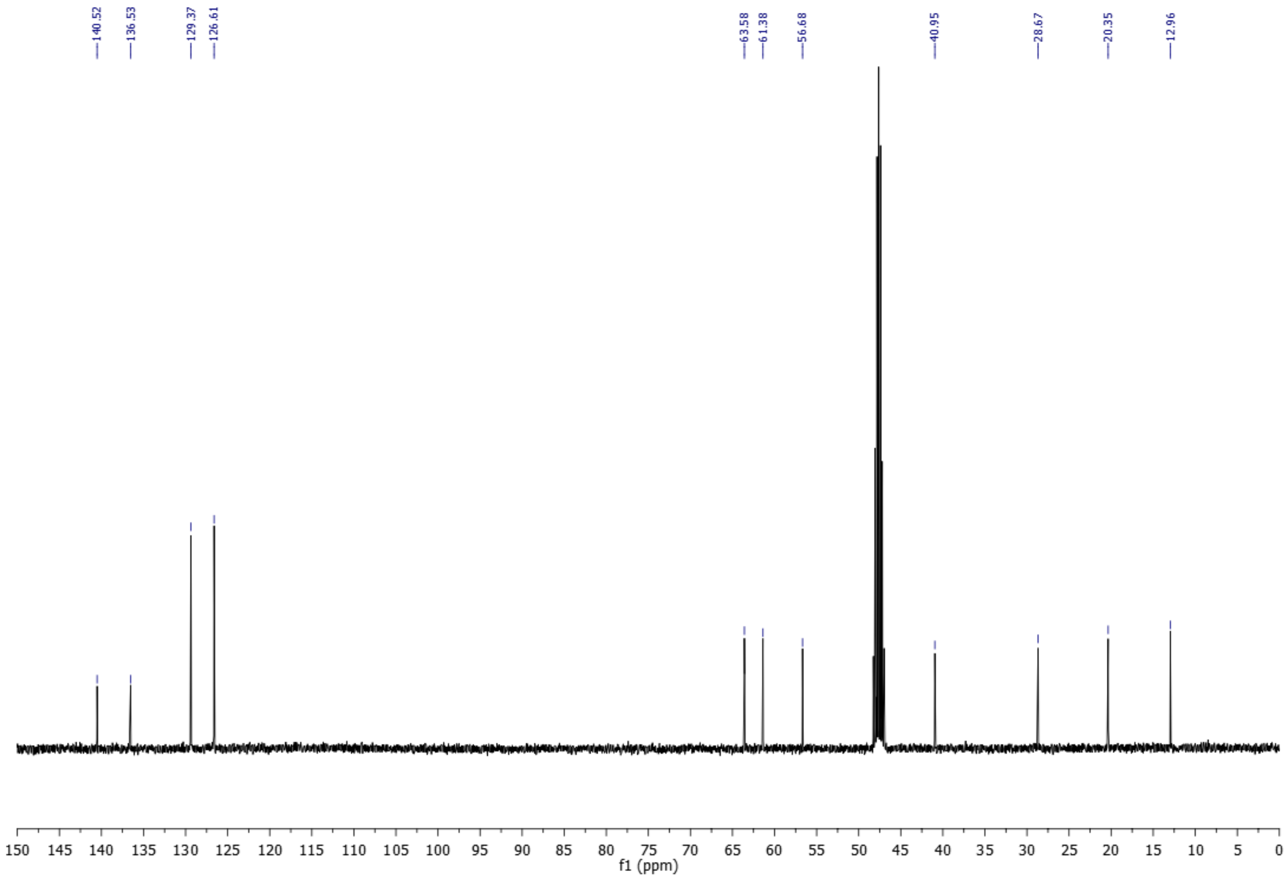

Figure S24. ^{13}C NMR spectrum (100 MHz, CD_3OD) of compound **7e**.

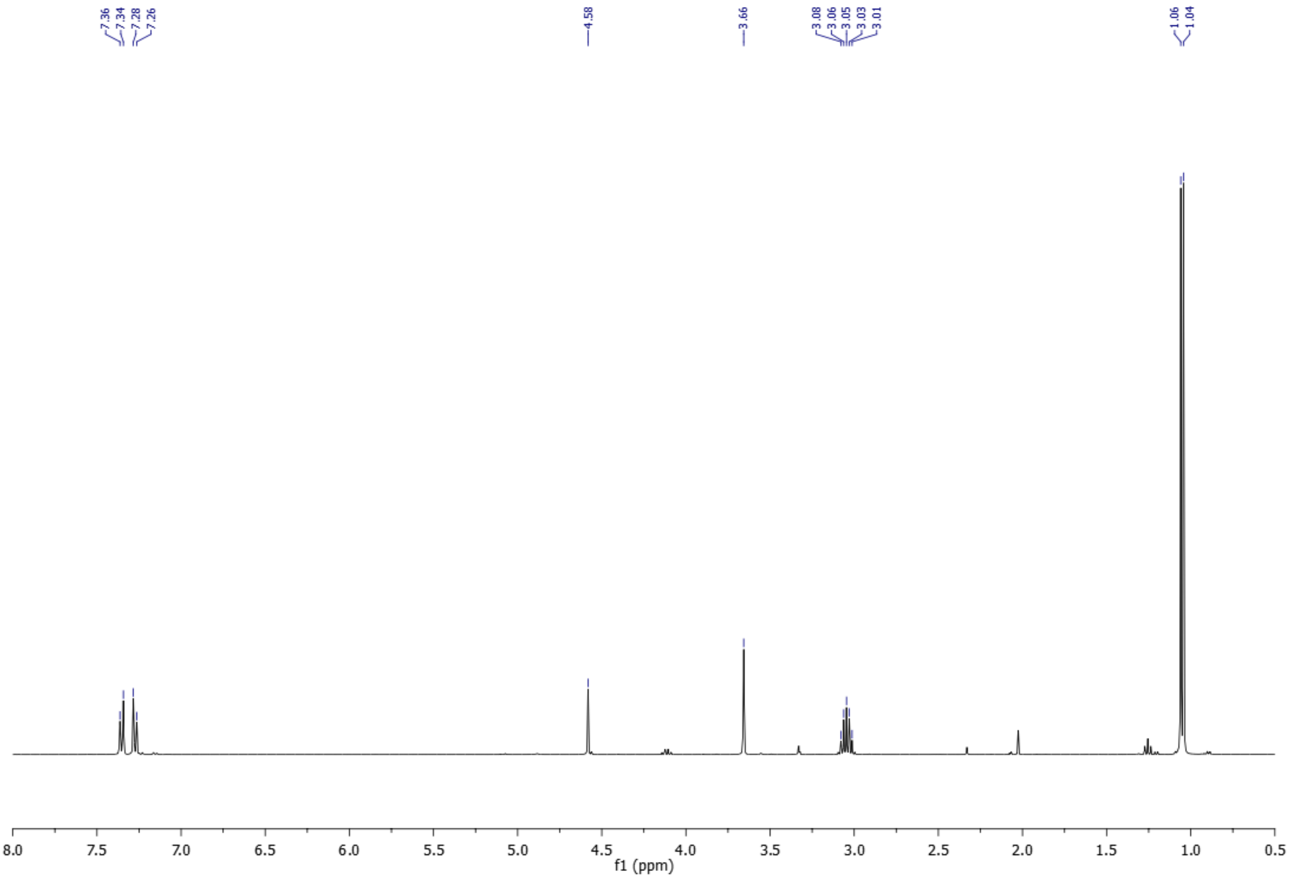

Figure S25. ^{1}H NMR spectrum (400 MHz, CD_3OD) of compound **7f**.

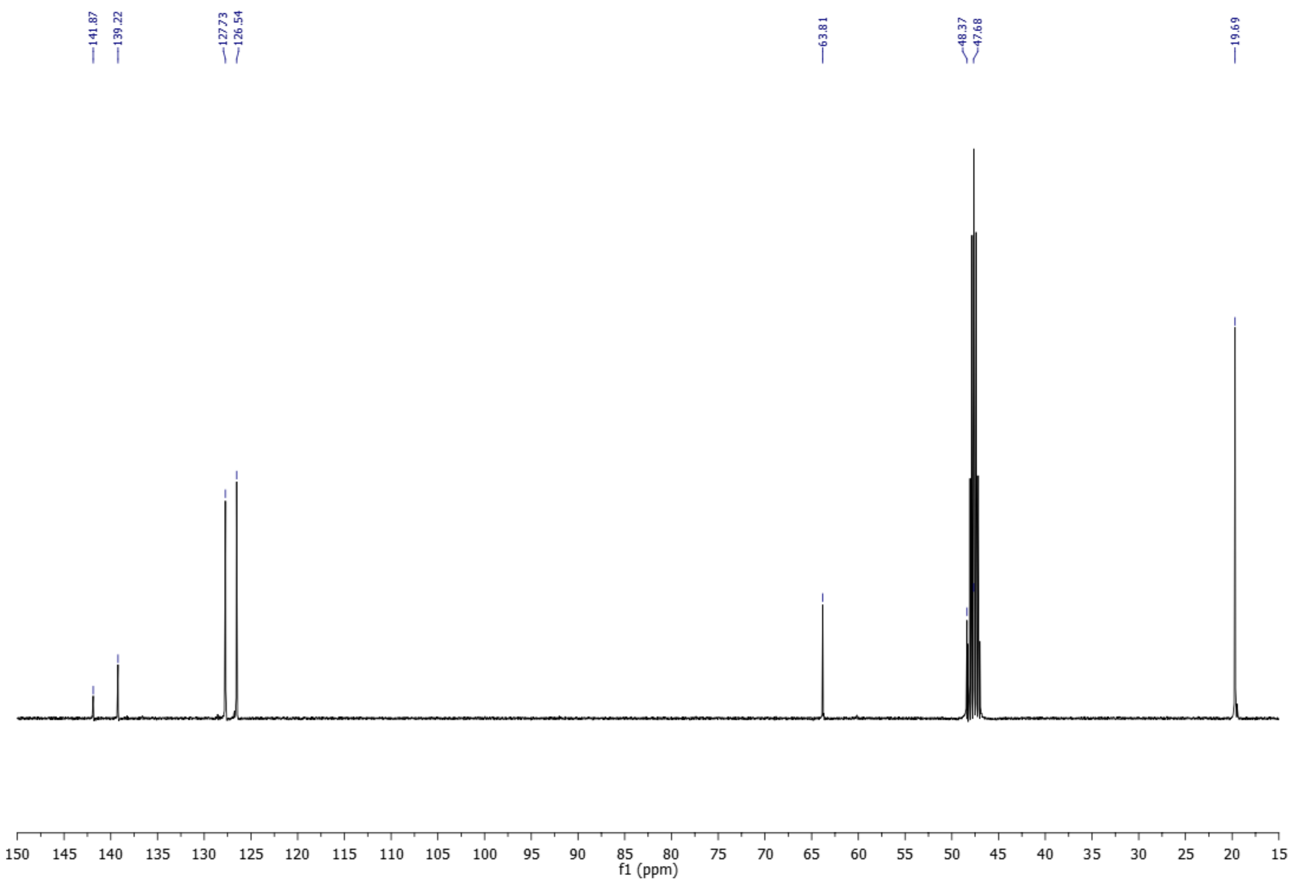

Figure S26. ^{13}C NMR spectrum (100 MHz, CD_3OD) of compound **7f**.

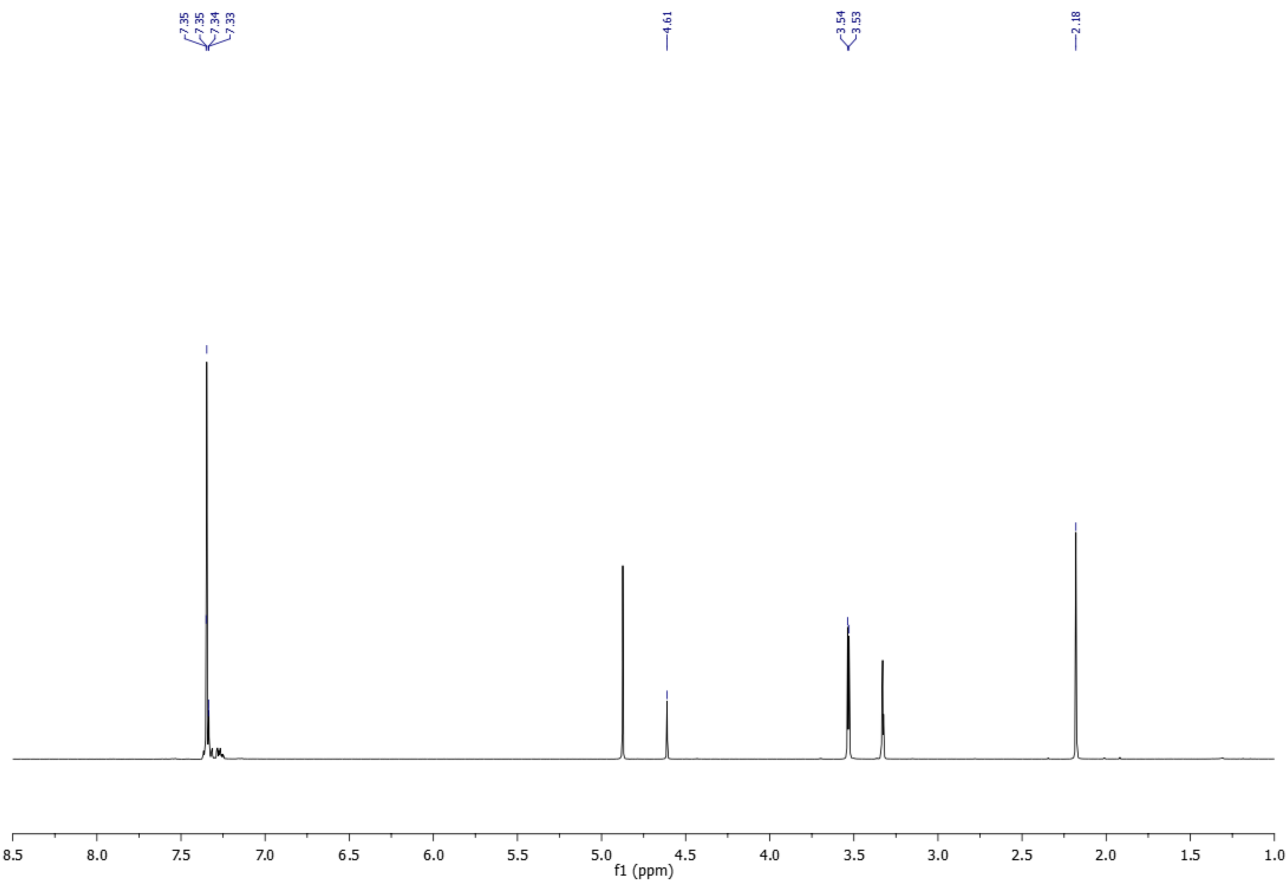

Figure S27. ^{1}H NMR spectrum (400 MHz, CD_3OD) of compound **7g**.

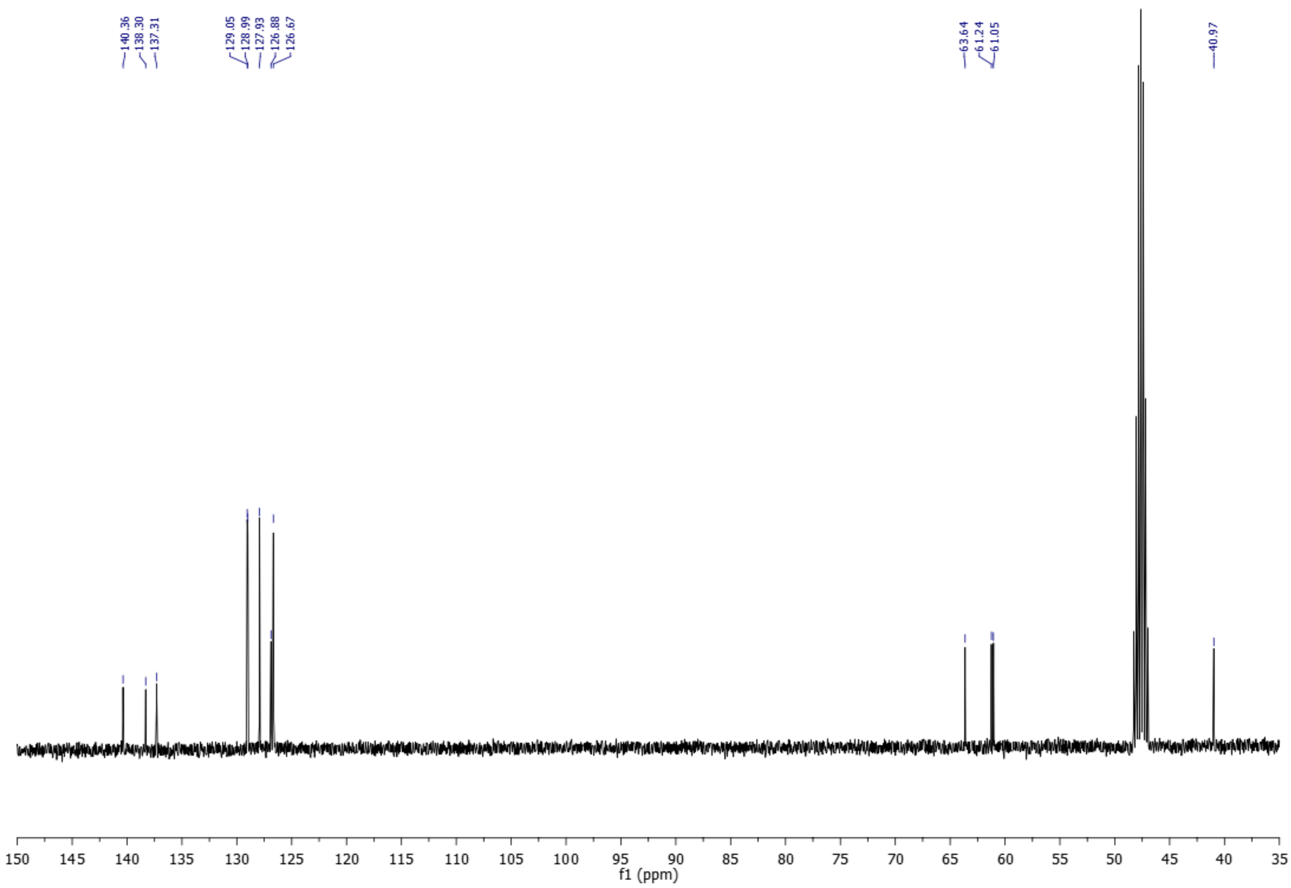

Figure S28. ^{13}C NMR spectrum (100 MHz, CD_3OD) of compound **7g**.

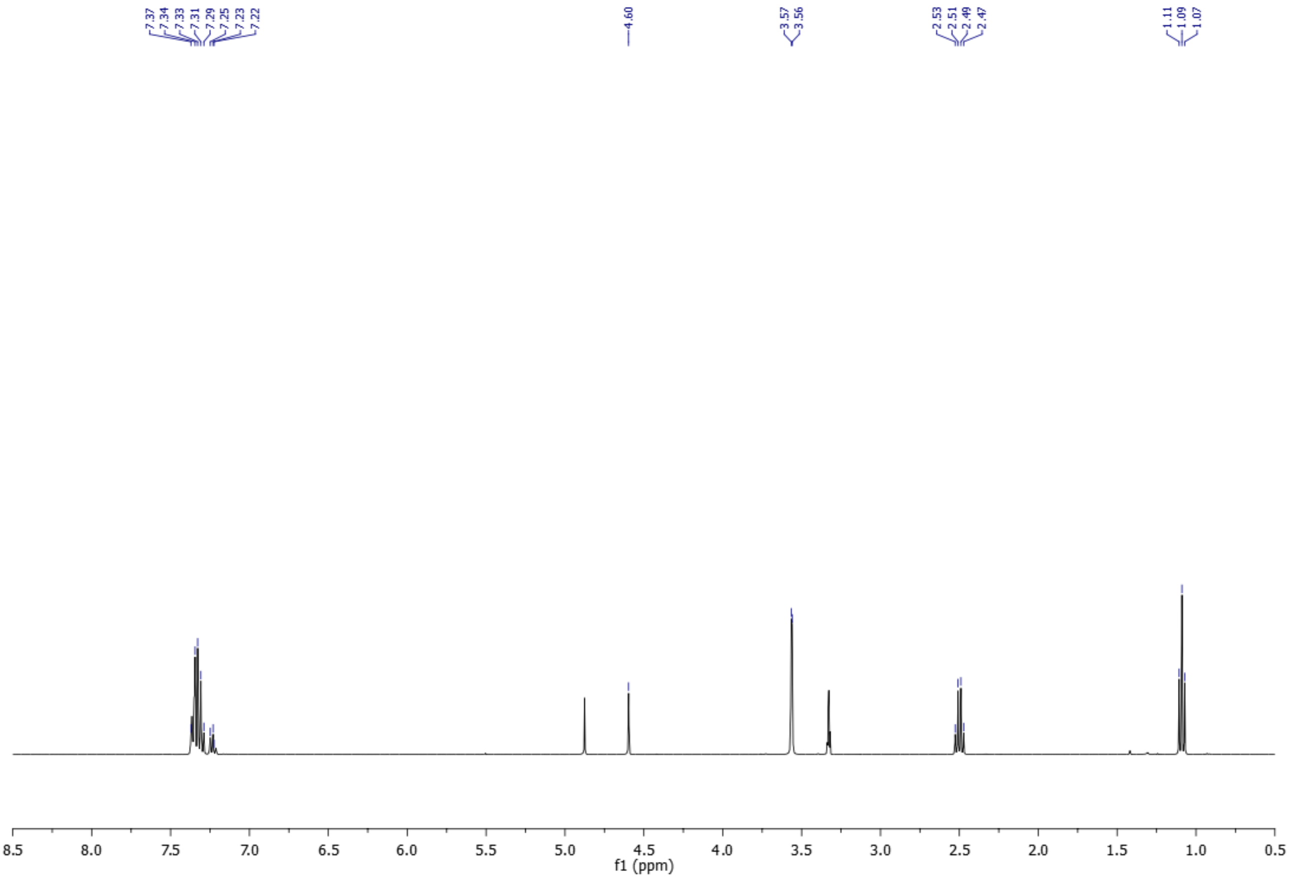

Figure S29. ^{1}H NMR spectrum (400 MHz, CD_3OD) of compound **7h**.

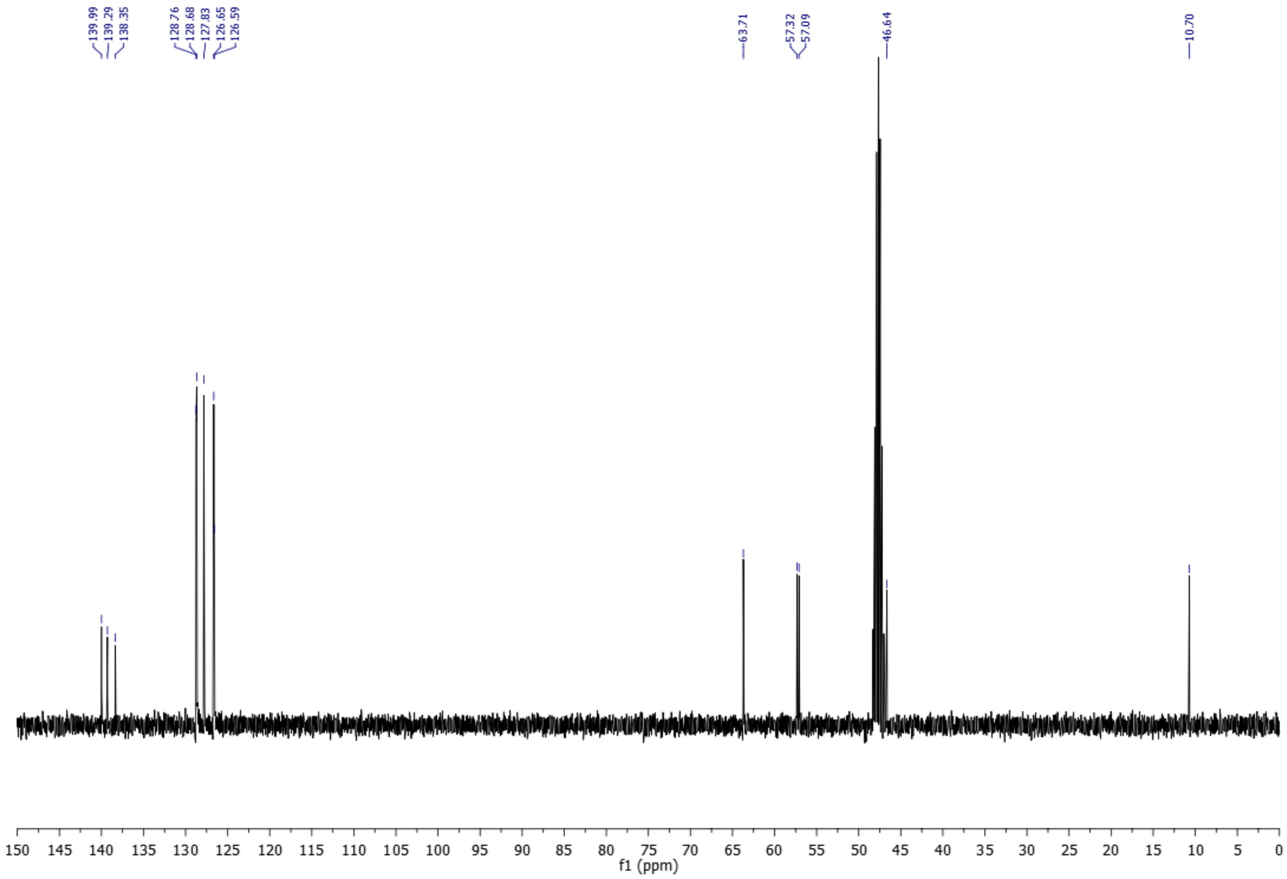

Figure S30. ^{13}C NMR spectrum (100 MHz, CD_3OD) of compound **7h**.

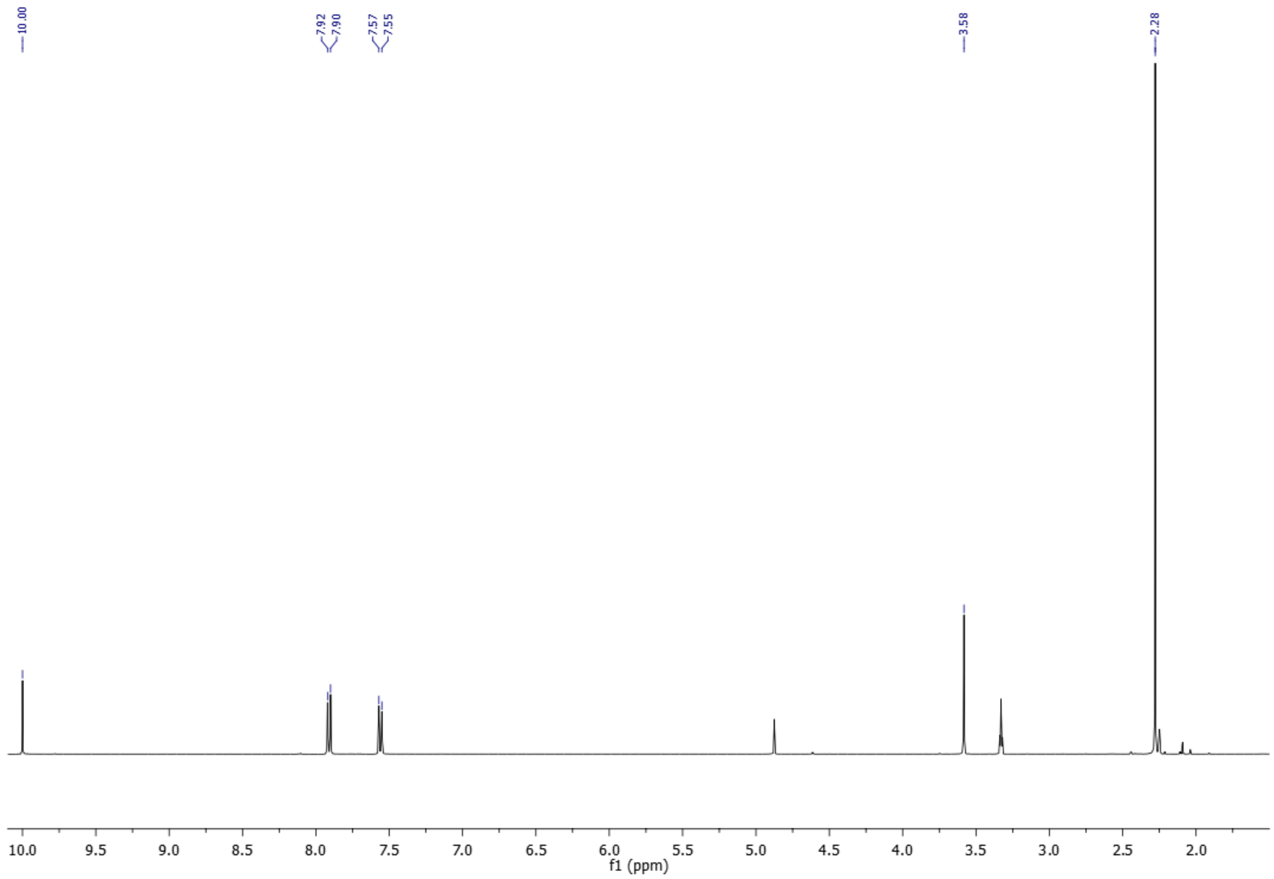

Figure S31. ^{1}H NMR spectrum (400 MHz, CD_3OD) of compound **4b**.

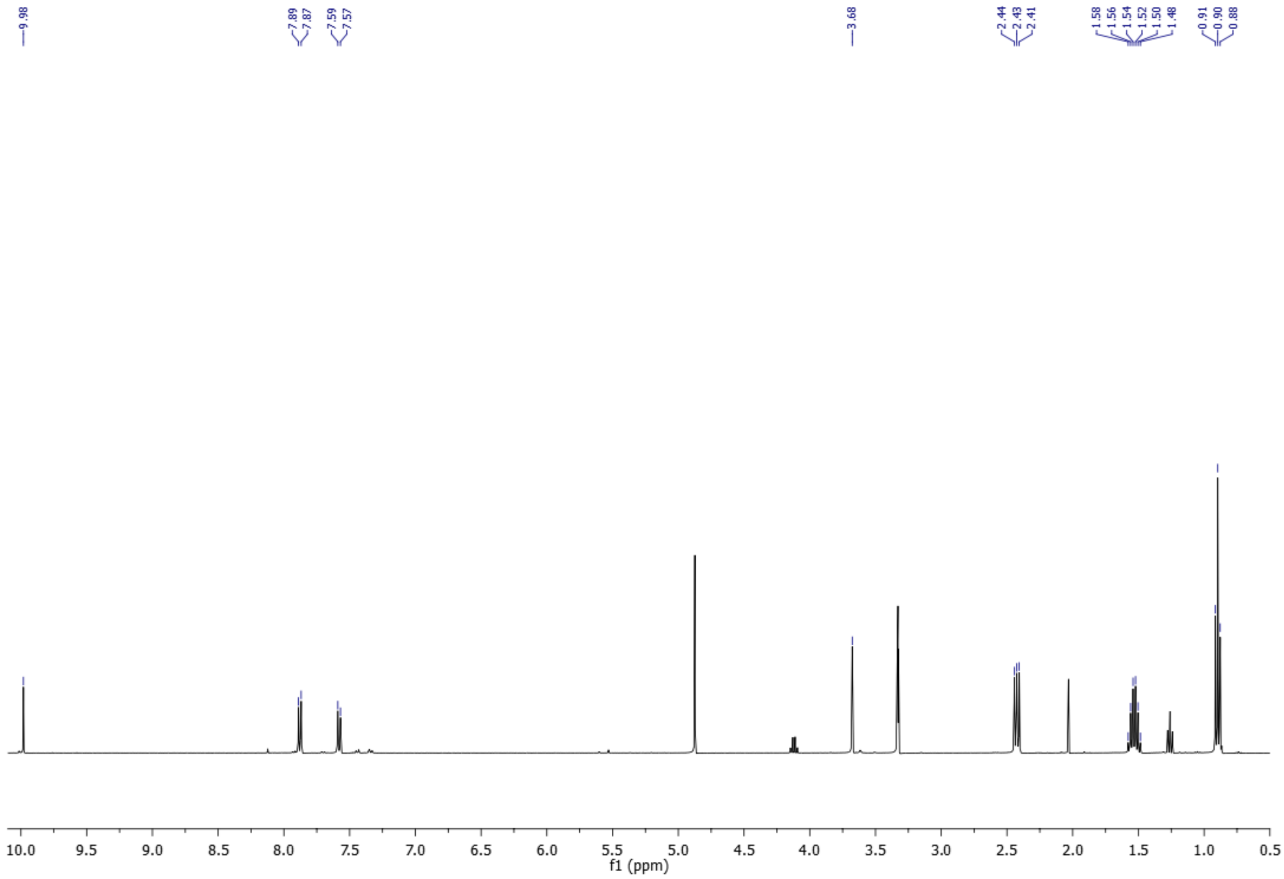

Figure S32. ^{1}H NMR spectrum (400 MHz, CD_3OD) of compound **4c**.

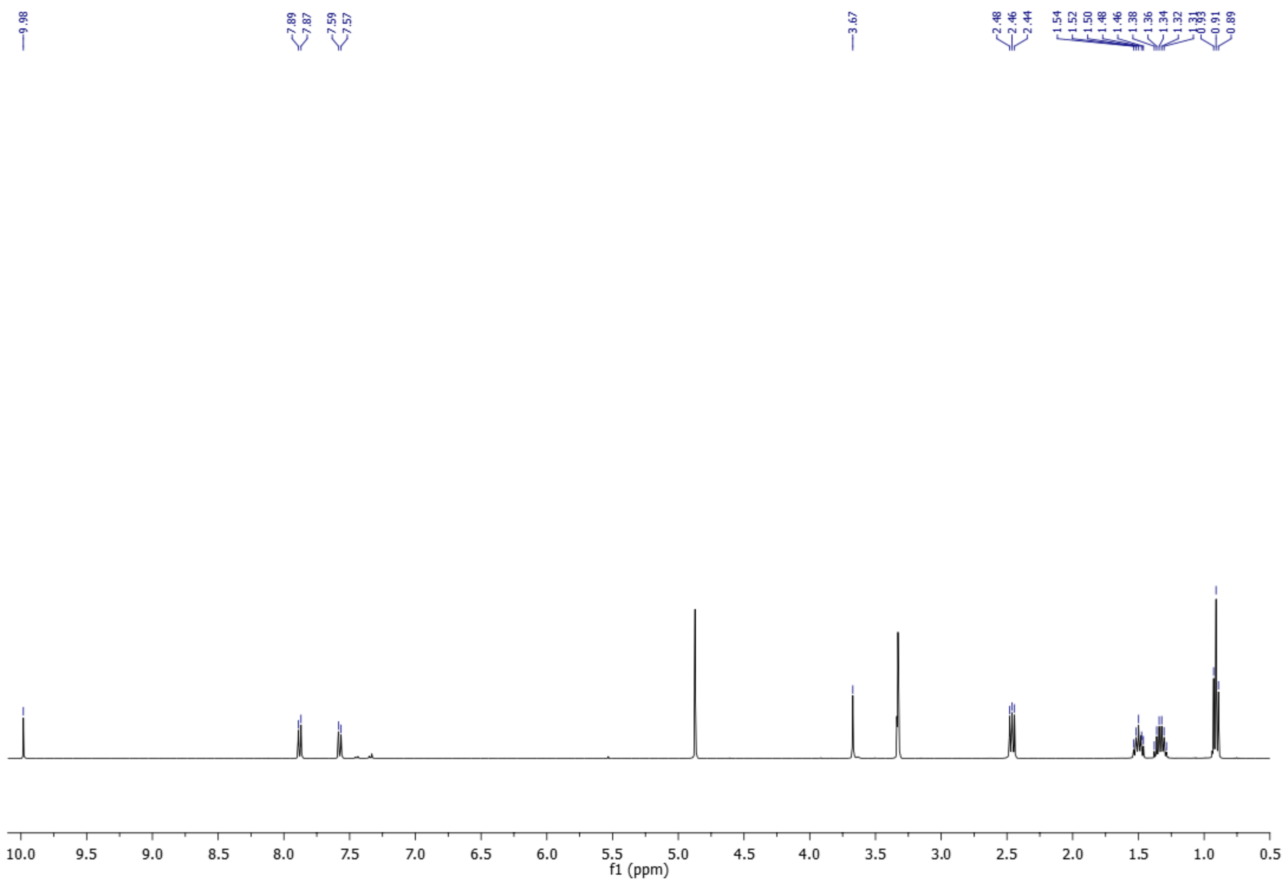

Figure S33. ^{1}H NMR spectrum (400 MHz, CD_3OD) of compound **4d**.

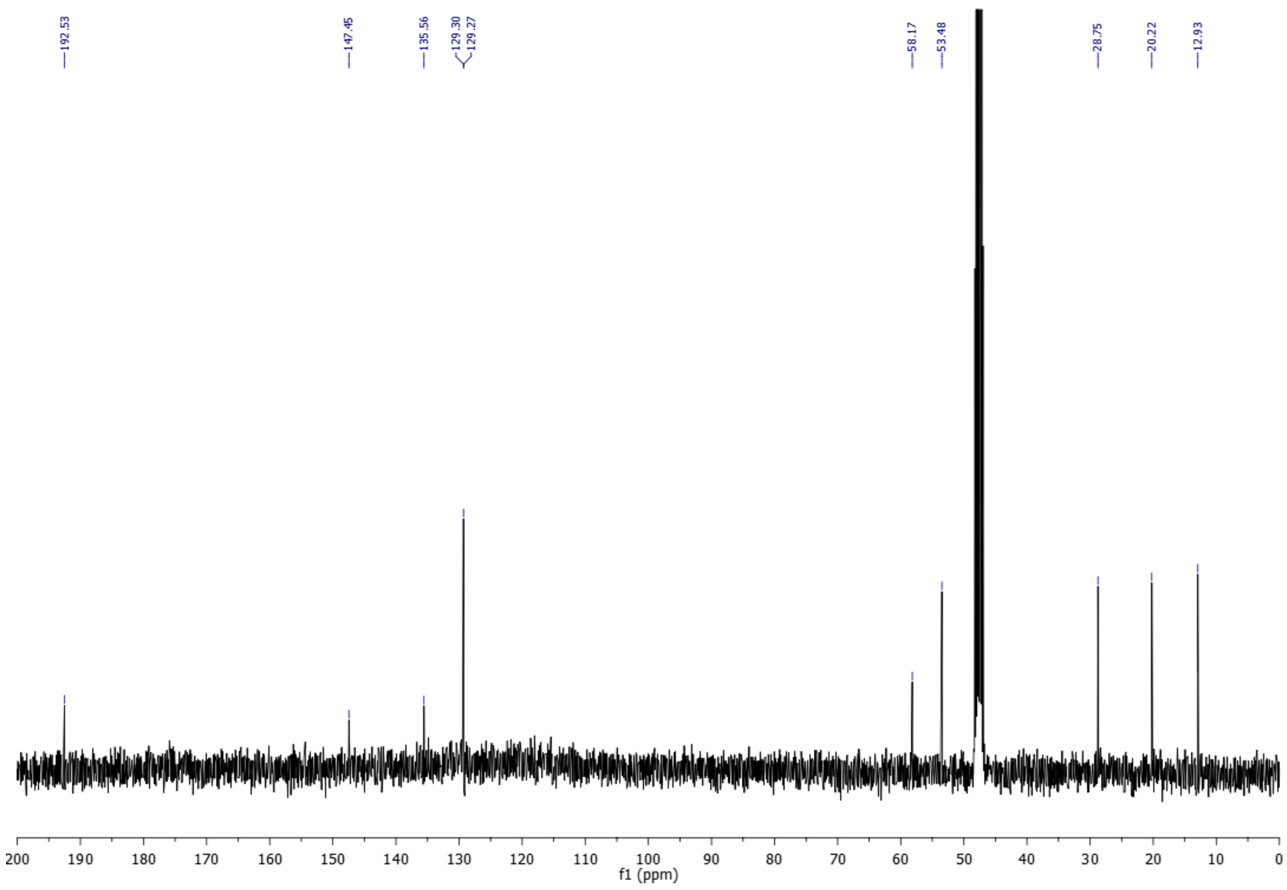

Figure S34. ^{13}C NMR spectrum (100 MHz, CD_3OD) of compound **4d**.

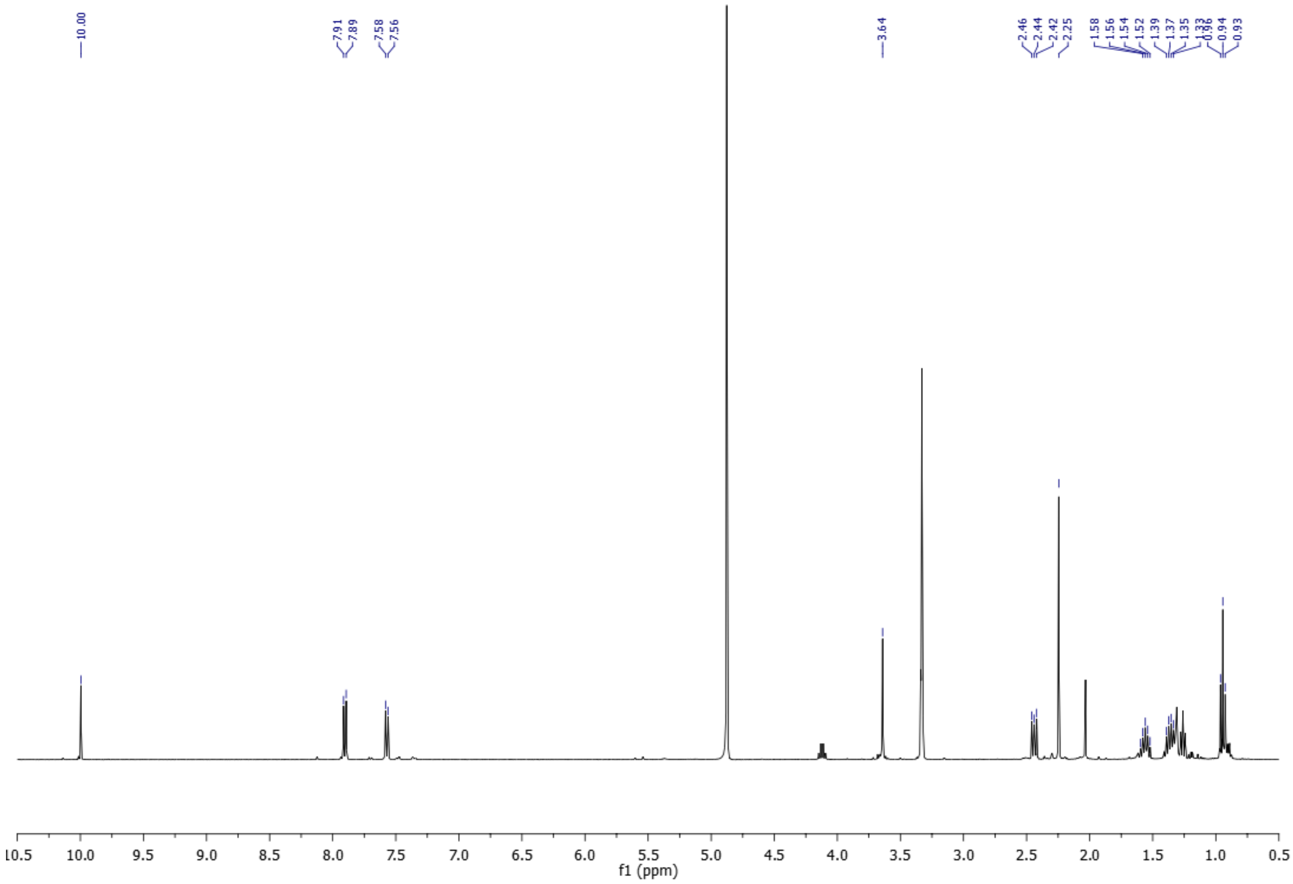

Figure S35. ^{1}H NMR spectrum (400 MHz, CD_3OD) of compound **4e**.

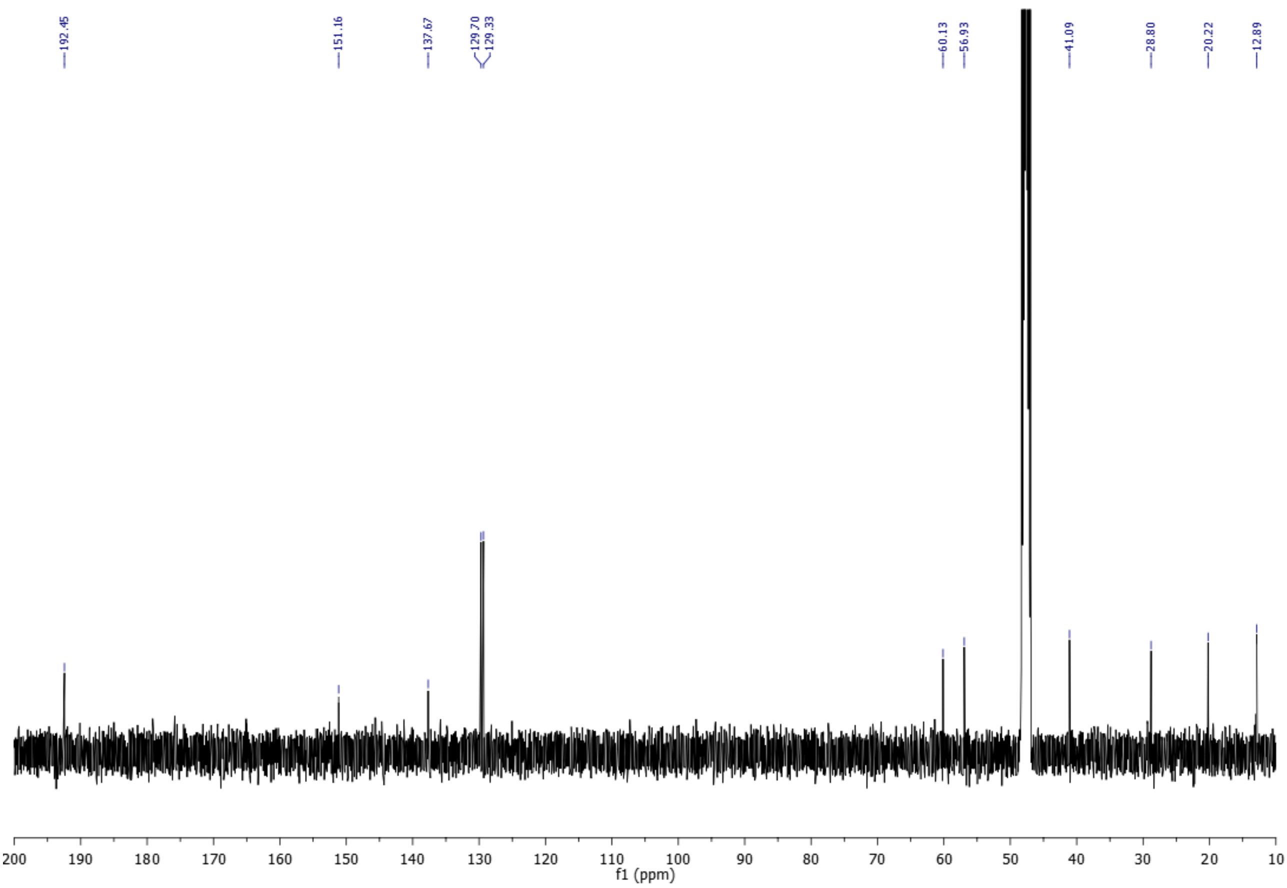

Figure S36. ^{13}C NMR spectrum (100 MHz, CD_3OD) of compound **4e**.

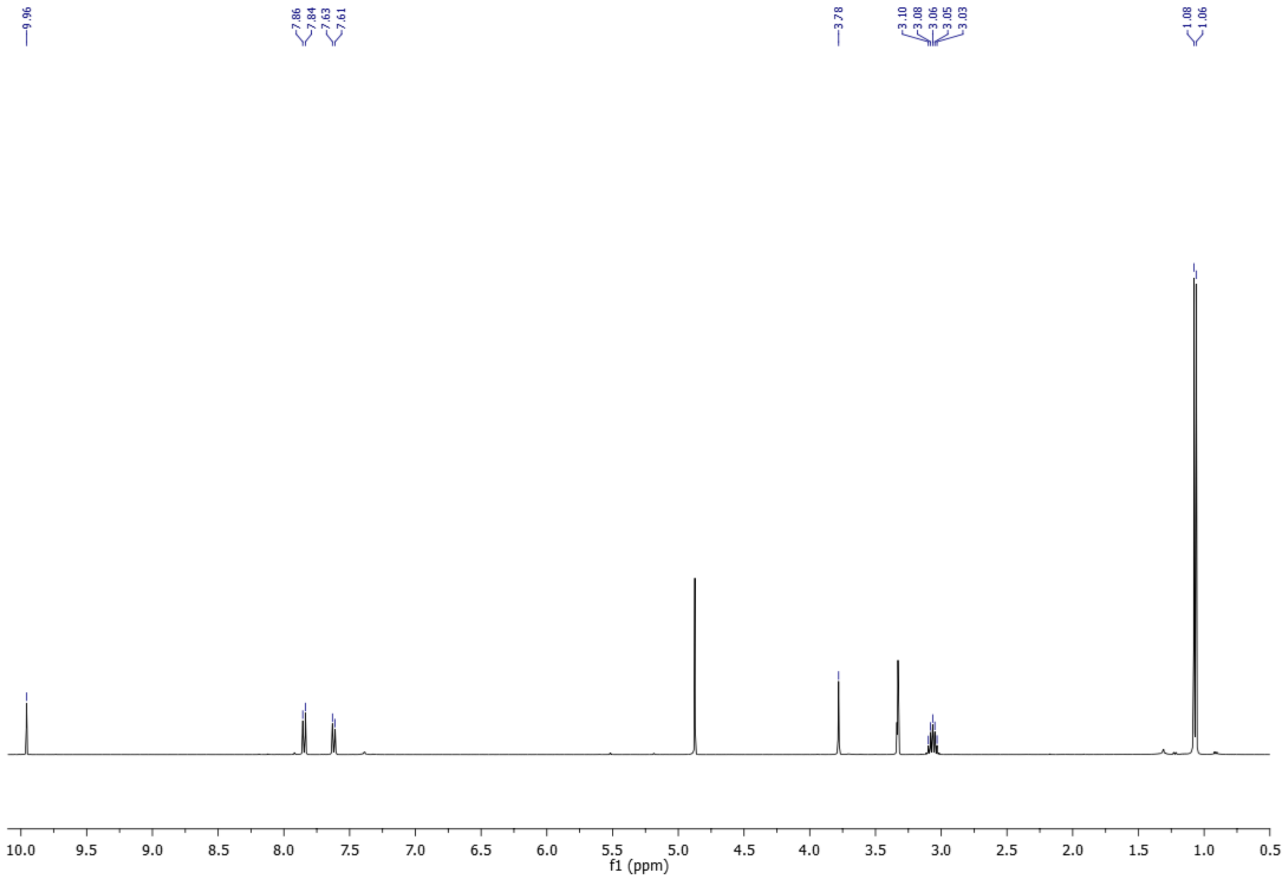

Figure S37. ^{1}H NMR spectrum (400 MHz, CD_3OD) of compound **4f**.

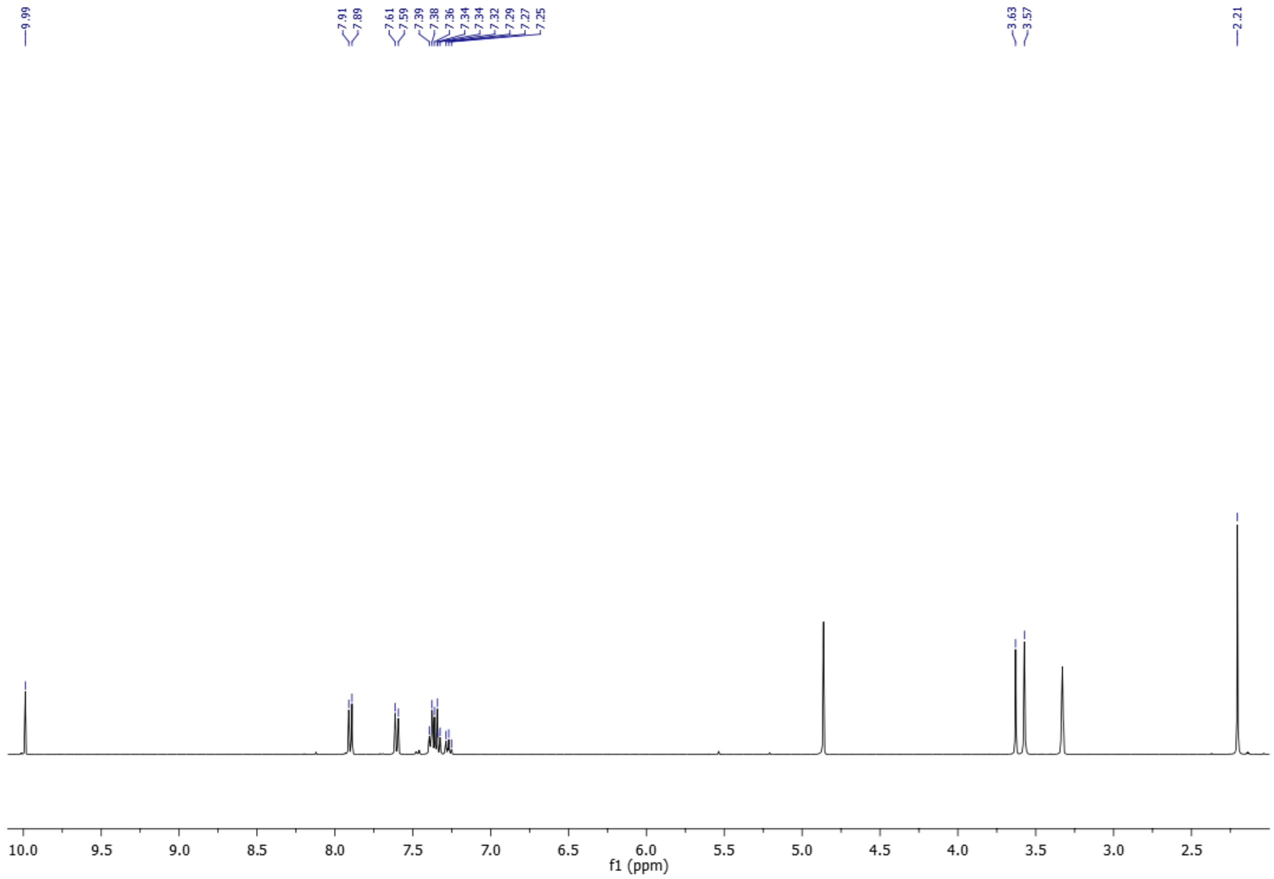

Figure S38. ^{1}H NMR spectrum (400 MHz, CD_3OD) of compound **4g**.

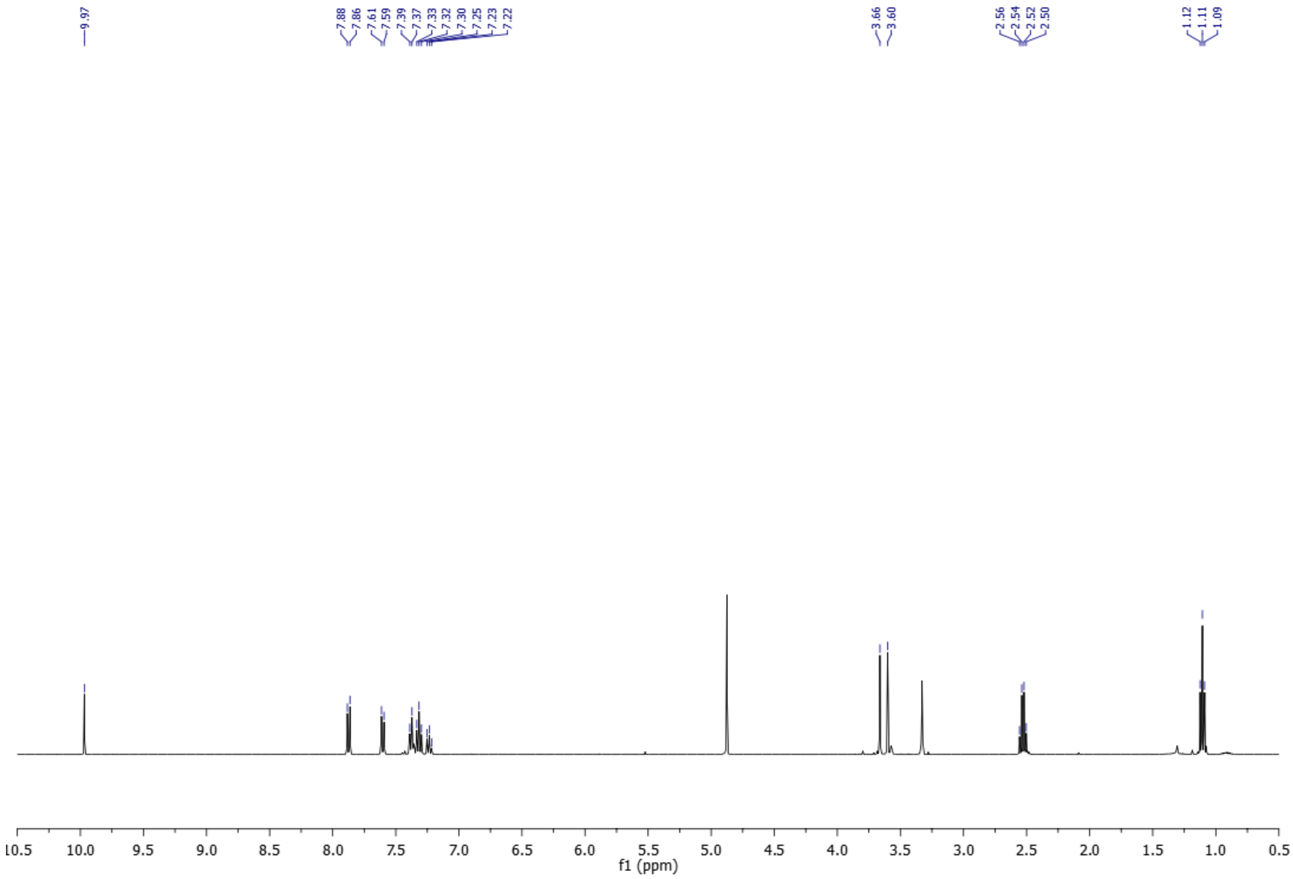

Figure S39. ^{1}H NMR spectrum (400 MHz, CD_3OD) of compound **4h**.

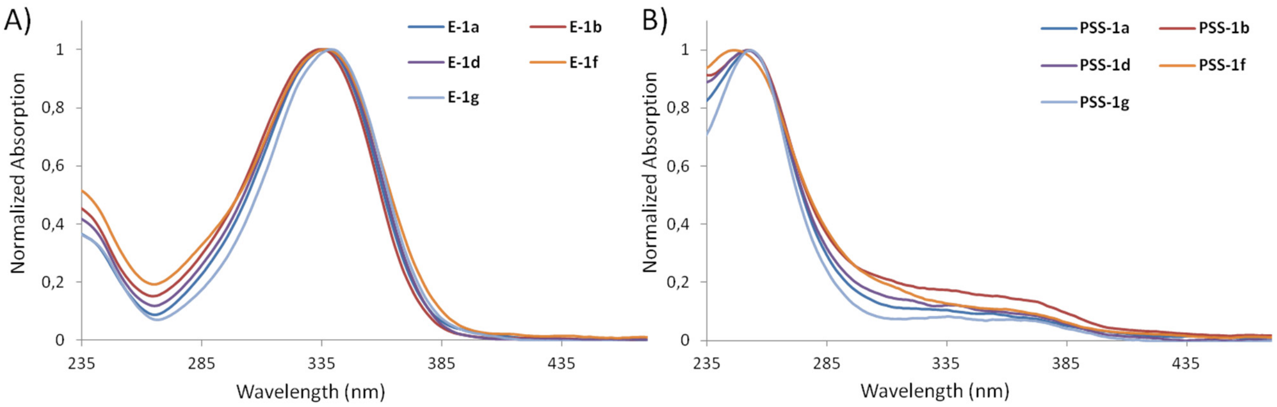

Figure S40. A) Normalized UV-Vis absorption spectra of methanolic solutions of compounds *E*-**1b,d,f,g**. B) Normalized UV-Vis absorption spectra of methanolic solution of compound **1b,d,f,g** at PSS generated by UV-B irradiation.

(a) (b)

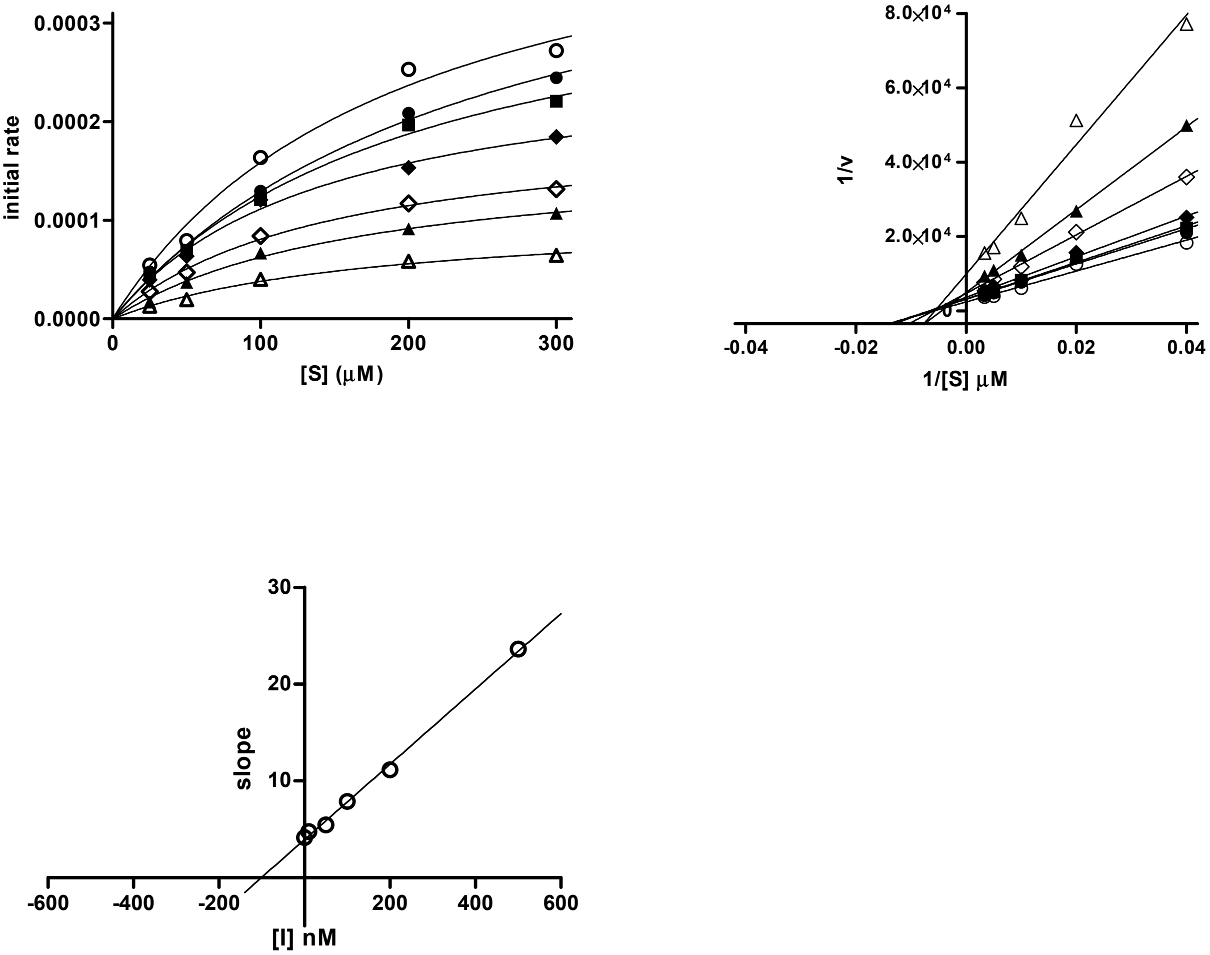

Figure S41. Inhibition kinetics (a) and Lineweaver–Burk plot (b), r^2 = 0.983–0.999 for *h*AChE (0.2 U/mL) and *E*-**1h** (0–500 nM) by using different substrate (acetylthiocholine iodide) concentrations (50–300 μM). The replot (r^2 = 0.997) of the slopes versus [I] determined the K_i (100 nM) as the x-axis intercept: (○) no inhibitor, (●) 10 nM, (■) 25 nM, (◆) 50 nM, (◇) 100 nM, (▲) 200 nM, (△) 500 nM.
